# The *Acinetobacter baumannii* Two-Component System AdeRS Regulates Genes Required for Multidrug Efflux, Biofilm Formation, and Virulence in a Strain-Specific Manner

**DOI:** 10.1128/mBio.00430-16

**Published:** 2016-04-19

**Authors:** Grace E. Richmond, Laura P. Evans, Michele J. Anderson, Matthew E. Wand, Laura C. Bonney, Alasdair Ivens, Kim Lee Chua, Mark A. Webber, J. Mark Sutton, Marnie L. Peterson, Laura J. V. Piddock

**Affiliations:** a Antimicrobial Agents Research Group, Institute of Microbiology and Infection, University of Birmingham, Edgbaston, Birmingham, United Kingdom; b Department of Experimental and Clinical Pharmacology, University of Minnesota, Minneapolis, Minnesota, USA; c Public Health England, Microbiology Services Division, Porton Down, Salisbury, United Kingdom; d Centre for Immunity, Infection and Evolution, University of Edinburgh, Edinburgh, United Kingdom; e Department of Biochemistry, Yong Loo Lin School of Medicine, National University of Singapore, Singapore

## Abstract

The opportunistic pathogen *Acinetobacter baumannii* is able to persist in the environment and is often multidrug resistant (MDR), causing difficulties in the treatment of infections. Here, we show that the two-component system AdeRS, which regulates the production of the AdeABC multidrug resistance efflux pump, is required for the formation of a protective biofilm in an *ex vivo* porcine mucosal model, which mimics a natural infection of the human epithelium. Interestingly, deletion of *adeB* impacted only on the ability of strain AYE to form a biofilm on plastic and only on the virulence of strain Singapore 1 for *Galleria mellonella*. RNA-Seq revealed that loss of AdeRS or AdeB significantly altered the transcriptional landscape, resulting in the changed expression of many genes, notably those associated with antimicrobial resistance and virulence interactions. For example, *A. baumannii* lacking AdeRS displayed decreased expression of *adeABC*, *pil* genes, *com* genes, and a *pgaC*-like gene, whereas loss of AdeB resulted in increased expression of *pil* and *com* genes and decreased expression of ferric acinetobactin transport system genes. These data define the scope of AdeRS-mediated regulation, show that changes in the production of AdeABC mediate important phenotypes controlled by AdeRS, and suggest that AdeABC is a viable target for antimicrobial drug and antibiofilm discovery.

## IMPORTANCE

*Acinetobacter baumannii* is a nosocomial pathogen and is an increasing problem in hospitals worldwide. This organism is often multidrug resistant, can persist in the environment, and forms a biofilm on environmental surfaces and wounds. Overproduction of efflux pumps can allow specific toxic compounds to be pumped out of the cell and can lead to multidrug resistance. This study demonstrates the role of the *A. baumannii* efflux pump AdeB, and its regulator AdeRS, in multidrug resistance, epithelial cell killing, and biofilm formation. Deletion of the genes encoding these systems led to increased susceptibility to antibiotics, decreased biofilm formation on biotic and abiotic surfaces, and decreased virulence. Our data suggest that inhibition of AdeB could prevent biofilm formation or colonization in patients by *A. baumannii* and provides a good target for drug discovery*.*

## INTRODUCTION

*Acinetobacter baumannii* is an opportunistic pathogen that commonly causes nosocomial infections, such as ventilator-associated pneumonia and skin, soft tissue, wound, and bloodstream infections (1). There are several factors that contribute to the environmental persistence of, and infection by, this organism. These include its propensity to resist desiccation and survive in the hospital environment (2, 3) and its ability to form biofilms on both medical devices and biological surfaces (4–7). *A. baumannii* isolates are often resistant to high concentrations of antimicrobial drugs because of both intrinsic and acquired mechanisms, such as increased production of multidrug resistance (MDR) efflux pump proteins (8–10).

Bacterial cells often exist as a biofilm, where cells are attached to a surface and enclosed in an extracellular matrix that can be composed of polysaccharides, extracellular DNA, and protein. Biofilms are significantly more resistant to antimicrobial treatment, host immune responses, desiccation, and UV light, which enables them to persist in harsh environments, including the hospital setting. Growth on medical devices and tissue surfaces can lead to biofilm formation and increase the risk of bloodstream and respiratory infections, as the biofilm acts as a reservoir of bacteria (11). *In vitro* studies have shown that biofilms can survive antibiotic concentrations of up to 1,000 times the MIC for a planktonic culture, and *in vivo*, bacteria that survive antibiotic exposure in a biofilm state can cause recurrence of infection once antibiotic treatment is stopped (12, 13). It is hypothesized that biofilm resistance to antimicrobial therapy is due to multiple factors: the slow penetration of the biofilm by the antibiotic, the heterogeneity of the biofilm creating an altered microenvironment in which antibiotics are less effective, the differentiation of biofilm cells into a protected slow-growing state including a large proportion of “persister cells,” and changed gene expression due to this altered growth state (12–15). Bacteria in a sessile state exhibit proteomic profiles distinct from those of their planktonic counterparts, suggesting that these cells differ functionally and physically (16). Analysis of the transcriptome of *A. baumannii* in planktonic and biofilm states has revealed distinct and specific expression patterns and demonstrated the complexity of the networks regulating biofilm formation (17–20). Increased expression of resistance-nodulation-division (RND) efflux pump genes in biofilm versus planktonic states was also seen (17). Although several genes involved in biofilm pathways have been identified in *A. baumannii* (4–7), relatively little is known about the factors controlling biofilm formation or their contribution to virulence in this pathogen. Expression of factors involved in biofilm formation can vary widely, depending on the model system used (18–21). Therefore, it is essential that clinically relevant model systems be used to evaluate the microbial factors that contribute to infectious biofilm formation.

Bacterial MDR efflux pumps can extrude a broad range of substrates from the cell, conferring resistance to numerous antibiotics, biocides, dyes, and detergents. Increased expression of MDR efflux pump genes such as *Acinetobacter* RND efflux pump genes *adeABC* leads to MDR and is commonly seen in clinical isolates of *A. baumannii* (8, 22). Changes in the expression levels of these MDR efflux pump genes are often due to mutations in the genes encoding the two-component system (TCS) AdeRS, which regulates *adeABC* (22). Mutations in *adeRS* can cause overproduction of AdeABC and lead to MDR (8, 22). Deletion of either *adeR* or *adeS* from isolates overexpressing *adeABC* results in increased susceptibility to antimicrobials and other substrates of this pump (22). Bacterial TCSs have previously been shown to be involved in the regulation of various functions, such as growth, competence, metabolism, adaptation to starvation, osmoregulation, and expression of toxins (23).

In this study, we investigated the effect of deletion of *adeRS* and *adeB* on antimicrobial susceptibility and the abilities of two *A. baumannii* strains to form a biofilm on abiotic and biotic surfaces and cause infection in *Galleria mellonella*. The transcriptomes of *A. baumannii* strains AYE (24) and Singapore 1 (S1) (25) and *adeRS* and *adeB* mutants thereof were determined by RNA sequencing (RNA-Seq). This allowed a distinction to be made between the effects of *adeB* deletion and those of *adeRS* deletion. Multiple genes encoding proteins with potential functions including drug resistance, biofilm formation, and virulence, such as those encoding RND efflux pumps, type IV pili, DNA uptake channels, and acinetobactin transport systems, were differentially expressed in these mutants.

## RESULTS

### Choice of strains for this study.

In this study, we used *A. baumannii* strain AYE as a well-characterized isolate that is MDR because of an Ala94Val mutation in *adeS* that has been previously associated with increased expression of *adeABC* (10, 26). A clinical isolate from Singapore, S1, that was more susceptible to antimicrobials than strain AYE was and had no mutation in *adeS* and therefore did not express a high level of *adeABC* was also studied. Together, these strains represent clones that are internationally successful (strain AYE is sequence type 1 [ST1] of international clone I, and S1 is ST40 and representative of strains causing infections in Southeast Asia) (see Fig. S1 in the supplemental material). Type strain ATCC 19606 (ST52) was included as a control in the *ex vivo* biofilm experiments as a strain that has a well-characterized biofilm phenotype (21, 27). Deletion of *adeRS* was carried out by a markerless deletion method (28). It was hypothesized that the phenotype displayed by AYEΔ*adeRS* might be due to decreased production of the tripartite MDR RND efflux pump AdeABC (as observed by RNA-Seq). To distinguish between the role of AdeRS and that of AdeABC in drug resistance, biofilm formation, and virulence in *A. baumannii*, an AdeB efflux pump deletion mutant of strain AYE and an AdeAB efflux pump deletion mutant of strain S1 were created. Gene deletion was confirmed by PCR and Sanger sequencing.

### Deletion of *adeRS* reduces susceptibility to antimicrobials and dyes and reduces levels of efflux in AYE.

**FIG 1:**
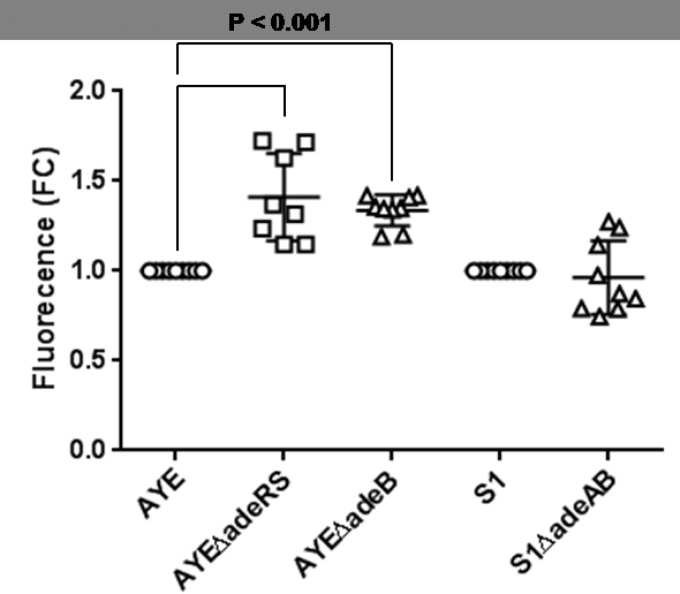
Accumulation of H33342 as determined by fluorescence. Markers show H33342 fluorescence at steady state compared with that of the parental strain in nine individual biological replicates, with horizontal lines representing the mean and vertical lines and whiskers showing the standard error of the mean. FC, fold change.

Growth curves, determined by measuring the optical density (OD) of the culture over time, showed no difference in growth rate between strain AYE and the *adeRS* mutant (see Fig. S2 in the supplemental material). However, deletion of *adeRS* resulted in a change in the drug resistance profile of strain AYE. There was a decrease in the MICs of imipenem, kanamycin, gentamicin, ciprofloxacin, tetracycline, tigecycline, chloramphenicol, and ethidium bromide upon the deletion of *adeRS* from strain AYE such that the phenotype of the mutant was similar to that of an *adeB* mutant of the same strain (Table 1). Although some of these changes were only 2-fold, which is considered to be the margin of error of this method, these changes were consistent in multiple experiments and were confirmed by growth kinetics in the presence of antibiotics (see Fig. S3 in the supplemental material). We have previously showed that accumulation of the dye Hoechst 33342 (H33342) is a good indication of efflux activity in *A. baumannii* (25). Accumulation of H33342 in strain AYEΔ*adeRS* was 40% greater than that in strain AYE (*P* < 0.05), indicating reduced levels of efflux in the mutant (Fig. 1). Similar results were seen with efflux of ethidium bromide (data not shown).

**TABLE 1:** MICs of dyes and antibiotics used in this study

Dye or antibiotic^a^	MIC^b^ (µg/ml) for:				
	AYE	AYEΔ*adeRS*	AYEΔ*adeB*	S1	S1Δ*adeAB*
AMP	>1,024	>1,024	>1,024	16	16
TAZ	1,024	1,024	1,024	1,024	1,024
IMP	1.5	**0.75**	**0.75**	0.125	0.125
MER	0.25	0.25	**0.125**	0.94	0.94
KAN	1,024	**512**	**256**	1	1
GEN	128	**8**	**4**	0.25	0.25
NOR	128	128	128	2	2
CIP	128	**32**	**32**	0.25	0.25
COL	1	1	1	0.5	0.5
TET	256	**128**	**128**	4	4
TIG	1	**0.25**	**0.25**	0.12	0.12
CHL	512	**256**	**256**	128	128
CCCP	32	32	**16**	16	16
PAβN	1,024	1,024	**512**	512	**128**
EtBr^b^	512	**256**	**128**	256	256

a AMP, ampicillin, TAZ, ceftazidime, IMP, imipenem; MER, meropenem; KAN, kanamycin, GEN, gentamicin, NOR, norfloxacin, CIP, ciprofloxacin, COL, colistin, TET, tetracycline, TIG, tigecycline, CHL, chloramphenicol; CCCP, carbonyl cyanide *m*-chlorophenylhydrazone; PAβN, phenylalanine-arginine beta-napthalymide; EtBr, ethidium bromide.

b Values in bold indicate significant differences between the mutant and parental strains.

### Deletion of *adeRS* in AYE results in decreased biofilm formation on mucosal tissue.

**FIG 2:**
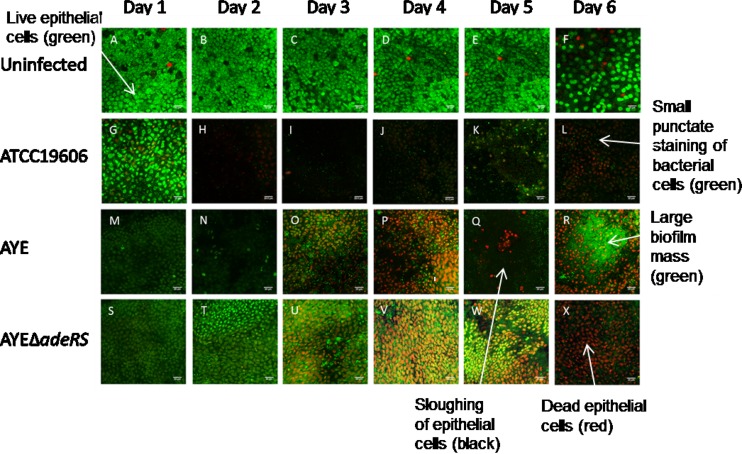
Time course of biofilm development on mucosa, observed by LIVE/DEAD staining and confocal laser microscopy. Uninfected epithelia are live (green) and intact throughout. Red, rounded epithelial cells indicate epithelial cell death. Small, punctate green staining indicates bacterial cells, and large, green-staining masses indicate bacterial biofilm. Black areas depict the exposed extracellular matrix. Arrows indicate examples of live and dead epithelial cells, bacterial cells, epithelial cell sloughing, and biofilm masses. (A to F) Uninfected epithelial cells. (G to L) Growth of strain ATCC 19606 on mucosal tissue. (M to R) Growth of parental strain AYE on mucosal tissue. (S to X) Growth of strain AYEΔ*adeRS* on mucosal tissue.

We used an *ex vivo* porcine vaginal mucosa (PVM) model to mimic a biofilm infection of human mucosa (20, 29, 30). To determine whether strain AYE showed a biofilm phenotype similar to that of a previously characterized biofilm-forming type strain of *A. baumannii* ATCC 19606, which has been shown to form a robust biofilm on both plastic and human skin equivalents (4, 21, 27), attachment of strain AYE and ATCC 19606 cells to PVM was imaged over 6 days by LIVE/DEAD staining and confocal laser scanning microscopy (Fig. 2). Viability of the tissue at 6 days was confirmed by imaging of uninfected tissue over the same time course. Uninfected tissue stained green, indicating live, intact cells with tight cell junctions (Fig. 2A to F). Epithelial cells and bacteria were distinguishable from each other on the basis of the size of the punctate staining, which could be clearly viewed by confocal microscopy. Over the 6-day time course, strains AYE and ATCC 19606 showed similar biofilm phenotypes. At 1 day postinoculation, loss of mucosal integrity was evidenced by rounding of epithelial cells and adherent bacteria (small, bright green, punctate staining) were visible on the tissue (Fig. 2G and M). By 3 days postinoculation, epithelial cell death (red) was observed and the number of bacteria visualized was greater (Fig. 2I and O). Black areas indicated sloughing of cells, as the extracellular matrix does not stain, and some biofilm formation was visible with ATCC 19606. By 6 days postinoculation, a large biofilm mass of both strains was visible, few dead epithelial cells remained, and most of the tissue was covered by biofilm (Fig. 2L and R).

To investigate whether AdeRS is important for biofilm formation on biotic surfaces, the growth of strains AYE and AYEΔ*adeRS* on PVM was measured. There was a rapid increase in the number of adherent cells on PVM up to the 1-day time point, followed by a slow but steady increase between 1 and 6 days (see Fig. S4 in the supplemental material). There was no significant difference between the numbers of adherent cells of the parental and mutant strains on days 1 to 6. However, LIVE/DEAD staining and confocal laser scanning microscopy showed a difference between the impact on the epithelial tissue of strains AYE and AYEΔ*adeRS* (Fig. 2). At 3 days postinoculation, strain AYE-infected tissue displayed epithelial cell death (red) (Fig. 2O), and by 6 days postinoculation, large biofilm masses and epithelial cell sloughing were visible, as described above (Fig. 2R). However, for AYEΔ*adeRS*, although epithelial cell death was evident and the tissue exhibited some epithelial cell sloughing, in contrast to coincubation with AYE, many dead epithelial cells were still visible and a biofilm had not covered the tissue. AYEΔ*adeRS* cells appeared as single attached cells, with no biofilm observed (Fig. 2V to X). These data suggest a critical role for AdeRS in mucosal biofilm infections and host cell cytotoxicity.

### Deletion of *adeRS* in AYE results in no change in biofilm formation on plastic.

**FIG 3:**
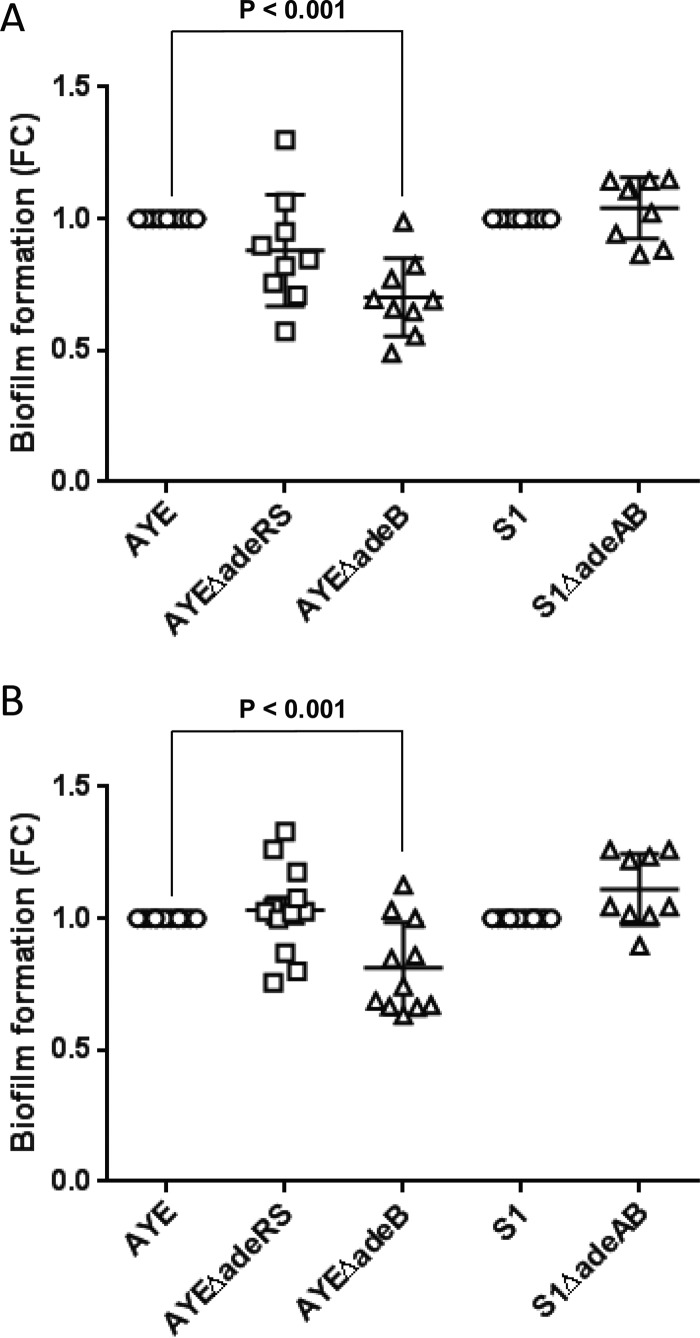
Biofilm formation on plastic pegs at 30°C and 37°C as determined by crystal violet staining. Panels: A, 30°C; B, 37°C. Markers show fold change (FC) in OD_600_ compared with the parental strain in individual biological replicates, with horizontal lines representing the mean and vertical lines and whiskers showing the standard error of the mean.

To investigate whether lack of AdeRS conferred a change in biofilm formation on an abiotic surface, we grew the parental strain AYE and the *adeRS* deletion mutant on plastic pegs for 8 h. At that time point, the biofilm formed by strain AYE was similar to that of previously characterized biofilm-forming strain ATCC 19606 (a range of time frames had been tested to determine the optimum length of time for biofilm growth to be measured accurately; data not shown). Pegs were then washed, and crystal violet staining was used to quantify biofilm mass. In this *in vitro* model, compared with the parental strain, there was no significant change in the biofilm mass produced by the *adeRS* deletion mutant at either 30°C or 37°C (Fig. 3). There was also no detectable change in the amount of pellicle formed in static cultures incubated for 48 h (data not shown).

### AYEΔ*adeRS* is as virulent as AYE in *G. mellonella.*

**FIG 4:**
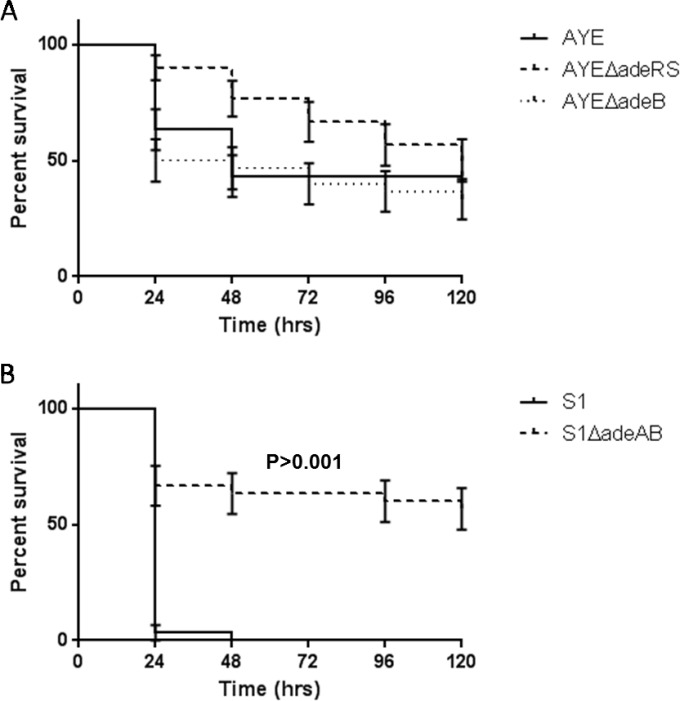
Kaplan-Meier survival curve showing the virulence of individual isolates in *G. mellonella*. (A) *adeRS* and *adeB* mutants of strain AYE. (B) *adeAB* mutant of strain S1. Data show percent survival (*n* = 30) of *G. mellonella* after inoculation with 10^6^ CFU of bacteria. Error bars represent the standard error of the mean.

Previous studies have shown a positive correlation between virulence in the *G. mellonella* infection model and mammalian models (31, 32). Furthermore, *G. mellonella* has been established as a good model system to study *A. baumannii* pathogenesis, as larvae can be maintained at 37°C and have both a cellular and a humoral immune response (33). In the *G. mellonella* model, compared with the parental strain, AYEΔ*adeRS* showed a small but nonsignificant change in virulence at an infectious dose of 10^6^ CFU (Fig. 4).

### Deletion of *adeRS* causes differential expression of functional gene groups in *A. baumannii* AYE.

To understand the role of the TCS AdeRS in the regulation of the expression of genes involved in antimicrobial drug resistance, biofilm formation, and virulence in *A. baumannii* strain AYE, changes in gene expression in the TCS deletion mutant AYEΔ*adeRS* were identified by RNA-Seq (see Tables S1 to S3 in the supplemental material).

**FIG 5:**
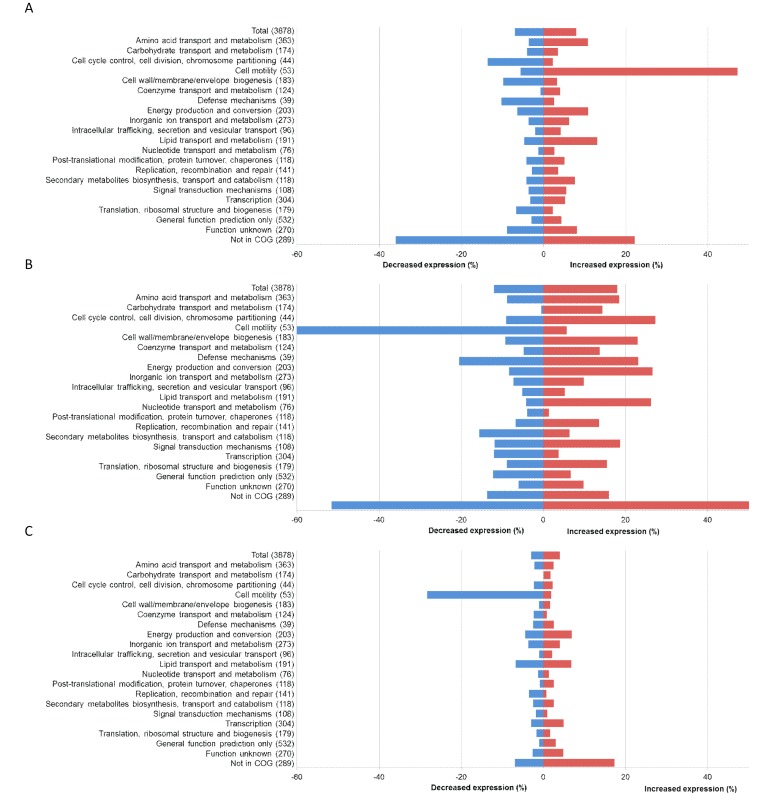
RNA-Seq results displayed by COG functions. Panels: A, AYEΔ*adeRS*; B, AYEΔ*adeB*; C, S1Δ*adeAB*.

Loss of AdeRS had a large impact upon the transcriptome of strain AYE, with the differential expression of 579 genes (308 genes with increased expression and 271 with decreased expression) (*P* < 0.05). Differentially expressed genes were categorized into clusters of orthologous groups (COGs) (34), and correlations with the phenotypic changes seen in AYEΔ*adeRS* were sought (Fig. 5). First, differential expression of genes known to confer antimicrobial resistance was identified. These included the RND efflux pump genes *adeABC*, which showed 128-, 91-, and 28-fold reductions in expression, respectively. One putative MDR RND efflux pump (ABAYE3036) had 1.9-fold increased expression, while the expression of another (ABAYE1796) was decreased by 1.5-fold. Genes encoding products with known and potential virulence functions, such as pili (35) and acinetobactin transport systems (36), had significant (*P* < 0.05) changes in expression levels in AYEΔ*adeRS.* For instance, there was increased expression of genes that encode motility, such as competence genes *comB*, *comC*, *comF*, *comL*, *comM*, *comN comO*, and *comQ* (2.5- to 9.8-fold). These genes putatively encode DNA uptake channels, and deletion of *comEC* has been shown to reduce DNA uptake, motility, and virulence in *G. mellonella* (37). There was also increased expression of *pilB*, *pilC*, *pilD*, *pilG*, *pilH*, *pilI*, *pilJ*, *pilT*, *pilU*, and *pilZ* (1.7- to 8-fold). These genes encode type IV pili, which participate in processes such as natural transformation and twitching motility (37, 38). Type IV pili have also been associated with the ability of *A. baumannii* to form a biofilm on plastic (39). Alterations in the expression of other biofilm genes included the decreased expression of a *pgaC*-like gene (2-fold) that putatively encodes a protein involved in the synthesis of cell-associated poly-β-(1-6)-*N*-acetylglucosamine, which is required for biofilm formation on abiotic surfaces (7), and the decreased expression of five putative biofilm genes, ABAYE0792, -1395, -1397, -1470, and -1473 (1.7- to 2.5-fold). In addition, multiple putative transport protein, outer membrane protein, and transcriptional regulator genes that lacked a comprehensive annotation showed differential expression.

Type IV pili are required for motility in *A. baumannii*, and inactivation of *pilT* has previously been shown to reduce twitching motility (37, 38). To determine whether the increased expression of motility genes correlated with altered motility, twitching motility and swarming experiments were carried out with 1% Mueller-Hinton agar and 0.3% Luria-Bertani (LB) agar, respectively (40). Strain AYE displayed twitching motility, typical of international clone I, but did not display swarming motility, like the majority of *A. baumannii* strains. However, no altered twitching motility or swarming phenotype was detected in strain AYEΔ*adeRS*.

### Deletion of *adeB* reduced efflux in AYE and S1 but only increased drug susceptibility in AYE.

Growth curves, determined by measuring the OD of the culture over time, showed no difference in growth rate between either strains AYE and AYEΔ*adeB* or S1 and S1Δ*adeAB* (see Fig. S1 in the supplemental material). To determine whether deletion of *adeB* conferred a change in the drug resistance profile of strain AYE, the MICs of commonly used antibiotics, dyes, and biocides for strains AYE, AYEΔ*adeB*, S1, and S1Δ*adeAB* were determined. There was a decrease in the MICs of meropenem, imipenem, kanamycin, gentamicin, ciprofloxacin, tetracycline, tigecycline, chloramphenicol, and ethidium bromide upon the deletion of *adeB* from strain AYE (Table 1). All 2-fold changes were confirmed by growth kinetics in the presence of antibiotics (see Fig. S3 in the supplemental material). AYEΔ*adeB* had altered susceptibility to many of the same drugs as AYEΔ*adeRS*, but the MICs of kanamycin, gentamicin, and ethidium bromide were lower after the deletion of *adeB*. Deletion of *adeAB* from S1 only resulted in a decrease in the MIC of phenylalanine-arginine β-naphthylamide (PAβN; an inhibitor of AcrB and MexB, homologues of AdeB), although the MICs of most drugs for S1 were much lower than those for strain AYE.

Compared with the steady-state accumulation level of the dye H33342 in the parental strain, that in AYEΔ*adeB* was increased by 33% (*P* ≤ 0.05) (Fig. 1), indicating reduced levels of efflux in this efflux pump gene deletion mutant. There was no difference between the accumulation of H33342 in S1Δ*adeAB* and that in its parental strain, S1; this was consistent with the minimal changes in MICs (Fig. 1).

### Deletion of *adeB* resulted in decreased biofilm formation of AYE on plastic.

To investigate whether AdeABC contributes to *A. baumannii* biofilm formation, we grew parental strains AYE and S1 and their isogenic efflux pump mutants on plastic pegs for 8 h and quantified the biofilm masses as described previously. Compared with the biofilm mass produced by the respective parental strains in this *in vitro* model, there was a significant decrease (*P* < 0.05) in that produced by AYEΔ*adeB* at both 30°C and 37°C (Fig. 3). However, there was no change in biofilm formation by S1Δ*adeAB* at either temperature (Fig. 3). There was also no change in the amount of pellicle formed in static cultures of AYEΔ*adeB* and S1Δ*adeAB* incubated for 48 h (data not shown).

### Deletion of *adeB* from AYE and *adeAB* from S1 results in decreased biofilm formation on mucosal tissue.

**FIG 6:**
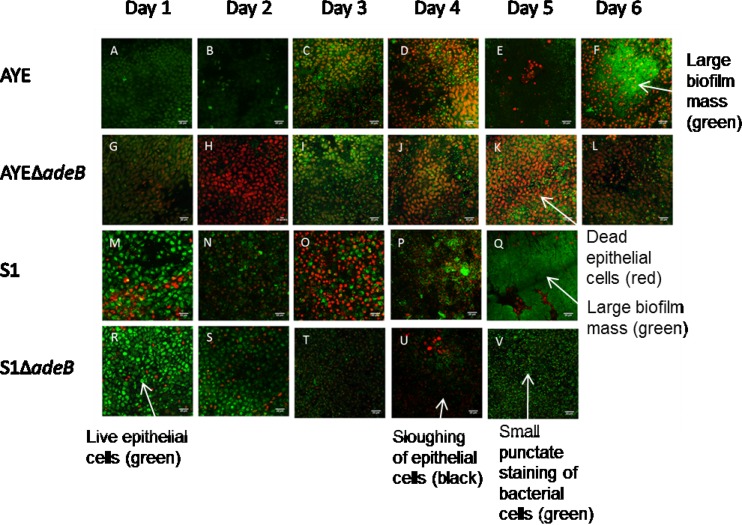
Time course of biofilm development on mucosa observed by LIVE/DEAD staining and confocal laser microscopy. Uninfected epithelia are live (green) and intact throughout. Red, rounded epithelial cells indicate epithelial cell death. Small, punctate green staining indicates bacterial cells, and large, green-staining masses indicate bacterial biofilm. Black areas depict the exposed extracellular matrix. Arrows indicate examples of live and dead epithelial cells, bacterial cells, epithelial cell sloughing, and biofilm masses. (A to F) Growth of parental strain AYE on mucosal tissue. (G to L) Growth of strain AYEΔ*adeB* on mucosal tissue. (M to Q) Growth of parental strain S1 on mucosal tissue. (R to V) Growth of strain S1Δ*adeAB* on mucosal tissue. Images are representative of at least three repeated experiments.

To determine the role of AdeABC in biofilm formation on biotic surfaces, the growth of strains AYE and S1 and their isogenic efflux pump mutants on PVM was measured as described previously. S1 showed growth similar to that of strain AYE in terms of both adherent cell counts (see Fig. S4 in the supplemental material) and biofilm imaging (Fig. 6). However, S1 was able to form a robust biofilm more rapidly than strain AYE and a thick biofilm mat could be seen by day 5 (Fig. 6Q), at which point the experiment was stopped. There was no significant difference in the number of adherent cells on the tissue between either strains AYE and AYEΔ*adeB* or S1 and S1Δ*adeAB* (see Fig. S4). However, compared with the respective parental strains, both efflux pump mutants showed a defect in biofilm formation when imaged by confocal microscopy. On days 5 and 6, S1 and AYE, respectively, formed large biofilm masses with extensive epithelial cell sloughing (Fig. 6F and Q), whereas although the mutants were able to cause epithelial cell death, only individual bacterial cells were observed to be attached to the mucosal tissue and less sloughing was evident (Fig. 6L and V). These data suggest that the AdeABC efflux pump plays a key role in biofilm formation on mucosal tissue and host cell cytotoxicity and that the decreased production of this MDR efflux pump may be responsible for the similar phenotype seen in strain AYEΔ*adeRS*.

### Deletion of *adeAB* from S1 results in attenuated virulence in *G. mellonella.*

S1Δ*adeAB* displayed attenuated virulence in the *G. mellonella* model, compared with that of S1. After infection with S1, all larvae were dead by day 2, whereas 60% were still alive on day 5 after infection with the mutant (Fig. 4). Strain AYE was less virulent than S1 in *G. mellonella*, and there was no significant difference in the killing of larvae by strains AYE and AYEΔ*adeB* (Fig. 4)*.* This suggests a strain-specific role for AdeABC in the virulence of *A. baumannii*.

### Deletion of *adeB* causes differential gene expression in *A. baumannii* AYE and S1.

Compared with strain AYE, AYEΔ*adeB* had increased expression of 693 genes and decreased expression of 477 genes (*P* < 0.05). Compared with S1, S1Δ*adeAB* had increased expression of 164 genes and decreased expression of 119 genes (*P* < 0.05). There were 108 genes with changed expression in both *adeB* mutants. Genes with increased expression in AYEΔ*adeB* included those encoding products with potential antibiotic resistance functions such as a tetracycline resistance protein, TetA (1.4-fold); membrane fusion protein (MFP) AdeT (1.4-fold), which is associated with MDR by active efflux in *A. baumannii* (41); a putative porin protein associated with imipenem resistance (ABAYE0924) (4.3-fold); and two putative MDR efflux systems (ABAYE1777, ABAYE3036) (1.4- and 3-fold). There was decreased expression of genes encoding a putative tetracycline resistance protein (ABAYE2235) (1.7-fold) and the lipid phosphoethanolamine transferase EptA (2.5-fold), which is associated with colistin resistance (42–44). Six β-lactamase genes (ABAYE0825, -1940, -2122, -2456, -3619, and -3623) also had altered expression (2.2- to 50-fold) in AYEΔ*adeB*. The only gene related to drug resistance that displayed differential expression, albeit only a small increase, in S1Δ*adeAB* was that for a putative MDR efflux system (ABAYE3036) (1.1-fold) that had increased expression in all three deletion mutants. Similar to strain AYEΔ*adeRS*, genes encoding products with known and potential virulence functions such as pili (35) and acinetobactin transport systems (36) also had significant changes in expression levels in *adeB* deletion mutants. The ferric acinetobactin transport system operon *bauDCEBA*, which encodes proteins required for persistence and virulence in the host (36, 45), had decreased expression in AYEΔ*adeB* (1.9- to 3.7-fold). The most striking change observed in both efflux pump mutants was in cell motility genes. The competence (*com*) genes which, as previously mentioned, are associated with DNA uptake, motility, and virulence, had significantly decreased expression in AYEΔ*adeB* and S1Δ*adeAB* (1.7- to 10-fold), in contrast to the significant increase in the expression of these genes in strain AYEΔ*adeRS* (Fig. 5). Likewise, there was a significant decrease in the expression of the type IV pilus genes, which are involved in natural transformation, twitching motility, and biofilm formation. One other putative biofilm-associated gene (ABAYE0792) showed increased expression (3-fold) in AYEΔ*adeB*, and no further genes with an annotated biofilm function were changed in S1Δ*adeAB*. However, multiple putative transcriptional-regulator-encoding genes (*araC*, *lysR*, and *tetR* family) and outer membrane protein-encoding genes that lacked a comprehensive annotation had differential expression in both mutants.

## DISCUSSION

We have shown that deletion of the TCS AdeRS is associated with decreased antibiotic resistance, epithelial cell killing, and biofilm formation on mucosal tissue. We hypothesized that AdeRS regulates the expression of genes, including the RND MDR efflux system genes *adeABC*, that are required for these functions. In strain AYE, deletion of *adeRS* led to significant changes in the global transcriptional landscape, with the changed expression of 579 genes, including 128-, 91-, and 28-fold changes in a*deA*, *adeB*, and *adeC* expression, respectively. We show here that, in this strain, deletion of *adeB* produces a phenotype similar to that caused by the deletion of *adeRS*, consistent with the hypothesis that the phenotype of AdeRS mutants is due to the decreased expression of this RND MDR efflux pump. However, deletion of *adeB* from an unrelated strain, S1, resulted in reduced epithelial cell killing and decreased biofilm formation on PVM but, in contrast to deletion in strain AYE, had little effect on antibiotic resistance and resulted in a reduction in virulence in *G. mellonella*. This suggests that AdeABC has a strain-specific role in *A. baumannii* and may perform different functions in different strains.

AdeABC has a well-defined role in resistance to antimicrobials in *A. baumannii* BM4587 (8, 46, 47). In our study, deletion of *adeRS* or *adeB* from strain AYE resulted in a decrease in the MICs of several antibiotics that have been previously described as substrates of this pump (46, 47). This indicates that the downregulation of *adeABC* in strain AYEΔ*adeRS* is responsible for the change in antibiotic susceptibility in this strain. Changes in MICs were more pronounced upon the deletion of *adeB* than upon the deletion of *adeRS*, which suggests that although deletion of *adeRS* results in an up to 128-fold decrease in the expression of *adeABC*, the efflux pump is still transcribed, albeit at a low level. We hypothesize that this is also responsible for the difference in the amounts of biofilm formed by these strains on plastic. While deletion of *adeB* from strain AYE resulted in a significant decrease in biofilm formation at both 30°C and 37°C, deletion of *adeRS* had no detectable effect. We hypothesize that low levels of expression of *adeABC* in strain AYEΔ*adeRS* are sufficient to maintain biofilm function, whereas inactivation of the pump in AYEΔ*adeB* significantly reduces the ability to form a biofilm.

Deletion of *adeAB* from a strain with a different background, clinical isolate S1, also resulted in a phenotype different from that seen in the strain AYE mutant: increased susceptibility to PAβN only. We hypothesize that the limited impact upon susceptibility to antimicrobial drugs after inactivation of AdeAB in S1 is a result of little change in the expression of *adeABC* between S1 and its mutant. Although *adeB* was expressed in S1, the level of expression was 16-fold lower in S1 than in strain AYE, as compared by RNA-Seq. This may explain the modest impact of the deletion of *adeAB* from S1 upon this strain’s susceptibility to antibiotics and why it is more susceptible to antibiotics than strain AYE is. Deletion of *adeAB* from S1 also had no significant effect on biofilm formation on plastic; this may also be due to the small impact of deleting a gene expressed at a low level.

In our study, only for AYEΔ*adeB* was a significant biofilm defect observed in the *in vitro* abiotic model. A similar observation was previously shown in an *adeB* deletion mutant of drug-susceptible *A. baumannii* BM4587 (47). However, all three of the mutants tested in our study showed reduced biofilm formation on mucosal tissue. The same phenotype was observed in AYEΔ*adeRS* and AYEΔ*adeB*, with a significant decrease in epithelial cell killing and biofilm formation seen in both. We hypothesize that the downregulation of AdeABC in the former is responsible for this biofilm defect. This suggests that regulation of *adeABC* is an important function of AdeRS in the ability of *A. baumannii* to form a biofilm on biotic surfaces. Although there was no change in the number of adherent cells on the mucosal tissue upon the deletion of *adeRS* or *adeB*, there was a clear difference in the structure of the biofilm when imaged by LIVE/DEAD staining and confocal laser scanning microscopy. This is a phenomenon that has been previously observed in *Staphylococcus aureus* (20). This suggests that while AdeB does not affect the initial attachment to tissue, cells lacking this efflux pump are unable to form a mature biofilm on mucosal tissue. Similar observations have been made in *Salmonella* efflux pump mutants, which are able to adhere to surfaces but are not able to produce a mature biofilm (48). PVM is made up of stratified squamous epithelium, similar in structure to human mucosal surfaces (29). Furthermore, the growth characteristics of *A. baumannii* on the PVM were similar to those observed in a three-dimensional human skin equivalent model (26). Therefore, differences in the ability of *A. baumannii* mutants to form a biofilm in the PVM model may have implications for respiratory and wound infections.

Despite a change in the expression of known (and putative) genes associated with virulence in *A. baumannii*, such as the acinetobactin iron acquisition system and pilin genes, deletion of *adeRS* or *adeB* from strain AYE had no effect on virulence in *G. mellonella*. It is possible that AYE does not express the *adeABC* efflux genes *in vivo*. In contrast, deletion of *adeAB* from S1 greatly reduced virulence. The MFP-encoding gene *adeA* is also partially deleted from strain S1, which may account for this difference. Previous studies with other species have shown that some MFPs, such as AcrA, can form a complex with multiple different RND components, such as AcrB and AcrF (49, 50). The presence of AdeA in strain AYE may allow it to interact with other proteins and so compensate for the lack of AdeB and ameliorate any impact upon virulence in this model. However, to our knowledge, so far, no other *A. baumannii* efflux pumps have been shown to play a role in virulence. Therefore, our data indicate a strain-specific role for the AdeABC efflux pump in virulence. A correlation between pathogenicity in *G. mellonella* and in humans has previously been observed with *A. baumannii* (33). Therefore, our data suggest that for some *A. baumannii* strains, such as S1, AdeABC may be required for infection of humans. The finding that the major RND MDR efflux pump in *A. baumannii* can also play a fundamental role in its ability to infect its host further underscores a role for MDR efflux pumps in the basic biology of pathogenic bacteria.

Using transcriptome profiling, we have demonstrated that deletion of AdeRS results in altered transcription patterns compared to those of the parental strain, thus highlighting its wider role in gene regulation in *A. baumannii*. Deletion of AdeRS modulates the expression of many genes, notably those associated with MDR, epithelial cell killing, and biofilm formation. Our data suggest that downregulation of the AdeABC efflux pump genes in the AYE *adeRS* deletion mutant is responsible for the increase in susceptibility to antimicrobials and the decrease in biofilm formation observed in this strain. This is the first time, to our knowledge, that this TCS has been associated with the regulation of biofilm formation in *A. baumannii*. Furthermore, we have shown a role for the AdeABC efflux pump in biofilm formation on plastic and mucosal tissue in an *ex vivo* model. These data suggest that inhibition of MDR efflux pumps may be a useful strategy to help prevent or treat colonization in patients by *A. baumannii*. Finally, we demonstrate that two AdeB efflux pump deletion mutations in different strains produced different virulence phenotypes in a *G. mellonella* model, highlighting the strain variability of *A. baumannii*. This suggests that broad conclusions about the roles of specific genes and proteins in the species should not be drawn from the study of single strains.

## MATERIALS AND METHODS

### Strains, media, and chemicals.

*A. baumannii* strain AYE is an MDR isolate that carries a mutation in *adeS* and is a member of epidemic clone II. *A. baumannii* S1 (DR00539/06) is a more drug-sensitive clinical isolate provided by the Network for Antimicrobial Resistance Surveillance (Singapore) and is representative of isolates causing infections in Singaporean hospitals (51). Strains AYEΔ*adeRS*, AYEΔ*adeB*, and S1Δ*adeAB* are unmarked deletion mutants created by a previously described method for generating markerless deletions in *A. baumannii* (28). Briefly, DNA fragments upstream and downstream of the genes of interest were amplified and ligated into a tellurite-resistant (*sacB*^+^ *xylE*^+^) suicide vector, pMo130-Tel^r^. A two-step selection process was then used to select for deletion mutants. Isolates S2 (DR01138/06), R2 (TTSH6013 654325/06), and R3 (DM01800/06) are representative Singapore clinical isolates used to compare the levels of *adeB* expression (28). Strain ATCC 19606 (obtained from the American Type Culture Collection, Manassas, VA) was used throughout as a reference strain with a previously characterized biofilm phenotype (4). All strains were routinely cultured on LB agar or tryptic soy agar II containing 5% sheep blood and in LB or Todd-Hewitt broth. All chemicals were from Sigma-Aldrich (Poole, Dorset, United Kingdom).

### Measurement of bacterial growth.

Bacterial strains were grown with aeration in LB broth at 37°C overnight. A total of 200 µl of culture was added to each well of a clear 96-well microtiter tray (MTT) at an inoculum density of 10^4^ CFU/ml. OD at 600 nm (OD_600_) was measured over 16 h in a BMG FLUOstar OPTIMA (BMG, United Kingdom) at 37°C. The difference in the final OD_600_ at 16 h was calculated, and values returning a *P* value of <0.05 from a Student *t* test were taken as significant.

### RNA-Seq.

RNA was extracted from a minimum of three biological replicates of each strain with a Qiagen RNAprotect Bacteria Reagent and RNeasy minikit and DNase treated with an Ambion TURBO DNA-*free* kit. RNA libraries were prepared and sequenced with an Illumina MiSeq or HiSeq at ARK Genomics, Roslin Institute, University of Edinburgh, University of Birmingham, or BGI Genomics, Hong Kong. Raw sequences were assessed for quality with FASTQC and processed. Alignments with a strain AYE reference genome (52) were performed with bowtie2. A bed file of the gene loci was generated from the gff annotation, and bedtools was used to count tags overlapping the regions of interest. Raw tag counts per sample were scale normalized to the sample by using the lowest number of tags within each data set. Counts were converted to log_2_ and quantile normalized within each series for comparisons within each data set. Pairwise comparisons of the normalized tag counts were performed by linear modeling (Bioconductor limma package). A raw *P* cutoff value of 0.05 was used to produce a list of changed genes that could be verified by phenotypic testing. No fold change cutoff was used.

### Measurement of efflux activity.

Efflux activity was determined by measuring the accumulation of H33342. The H33342 (bis-benzimide) assay was carried out as described previously (53). Steady-state fluorescence values were taken and converted to fold changes compared to the parental strain. The differences between parent and mutant strains were calculated, and values returning a *P* value of <0.05 from a Student *t* test were taken as significant.

### Determination of antimicrobial susceptibility.

The MICs of ampicillin, ceftazidime, kanamycin, gentamicin, norfloxacin, ciprofloxacin, colistin, tetracycline, tigecycline, chloramphenicol, carbonyl cyanide *m*-chlorophenylhydrazone, PAβN, and ethidium bromide were determined by the agar doubling dilution method according to British Society for Antimicrobial Chemotherapy standard methodology (54). MICs of imipenem and meropenem were determined by E test (bioMérieux, Hampshire, United Kingdom). Clinically relevant resistance was defined as a MIC above the recommended breakpoint according to the EUCAST guidelines (55). All experiments were carried out in triplicate.

### Measurement of biofilm formation on plastic.

Biofilm formation on plastic was measured with an adapted version of a previously described method (56). Wells of an MTT were inoculated with 100 µl of diluted culture, and a sterile, 96-well polystyrene PCR plate was placed into the MTT. Plates were incubated for 8 h at either 30°C or 37°C. Pegs were washed and stained with 1% crystal violet for 15 min. Dye was solubilized in 70% ethanol, and OD_600_ was measured with a BMG FLUOstar Optima. OD_600_ was converted to fold change compared to the parental strain in order to compare results between experiments. The differences between parent and mutant strains were calculated, and values returning a *P* value of <0.05 from a Student *t* test were taken as significant.

### Measurement of biofilm formation on PVM tissue.

Biofilm formation on mucosal tissue was measured as described previously (29). Briefly, specimens of normal PVM were excised from animals at slaughter and washed in RPMI 1640 medium (Invitrogen, Carlsbad, CA) supplemented with 10% fetal calf serum (Invitrogen), penicillin (50 IU/ml; MP Biomedicals, Solon, OH), streptomycin (50 µg/ml; MP Biomedicals), and amphotericin B (2.5 μg/ml; HyClone, Logan, UT); 5-mm tissue explants were cut; and excess muscle was trimmed away. Tissue explants were washed in serum- and antibiotic-free medium three times. Explants were then placed mucosal side up on a 0.4-µm cell culture insert (BD Bioscience, San Jose, CA, USA) in six-well plates containing fresh serum- and antibiotic-free RPMI 1640 medium. Explants were inoculated with 10^5^ CFU of bacteria and incubated at 37°C. Adherent and planktonic cells were counted at 24, 48, 72, 96, 120, and 144 h. The difference in the number of adherent cells between parent and mutant strains was calculated, and values returning a *P* value of <0.05 from a Student *t* test were taken as significant. For imaging, explants were stained with a FilmTracer LIVEDEAD Biofilm Viability kit (Invitrogen, Carlsbad, CA) according to the manufacturer’s instructions. The LIVE/DEAD stain consists of SYTO9, which stains live cells green, and propidium iodide (PI), which stains dead cells red. PI penetrates only cells with damaged membranes, and once it is in close proximity to SYTO9, it quenches the green signal, so only the red is visible. This allows live versus dead cells to be detected. These dyes are nonspecific nucleic acid dyes that do not discern between prokaryotic and eukaryotic cells (29, 57–59). After staining, specimens were gently washed three times in Hanks’ balanced salt solution and transferred to glass slides. A coverslip with a 1-mm spacer (Electron Microscopy Sciences, Hatfield, PA) was then applied, and specimens were imaged on an Olympus FluoView 1000 BX2 (Olympus America Corporation, Center Valley, PA) with a 60× oil immersion objective. Images were captured and processed with FluoView software (Olympus America Corporation, Center Valley, PA).

### Measurement of motility.

Swarming and twitching motility was measured as described previously (40). Briefly, a single colony was used to inoculate petri dishes containing 0.3% LB agar or 1% Mueller-Hinton agar. Plates were incubated overnight at 37°C and examined for growth.

### Measurement of virulence in *G. mellonella.*

Survival in *G. mellonella* was assayed as previously described (60). Bacteria were injected into *G. mellonella* larvae in an inoculum of 10^6^ CFU. Larvae were incubated statically at 37°C in petri dishes, and the number of dead larvae was scored periodically. All experiments were carried out in triplicate, and statistical analysis was carried out with GraphPad Prism 5 to produce Kaplan-Meier survival curves. The statistical significance of differences between parent and mutant strain survival curves was calculated with a log rank test. *P* values of <0.05 were taken as significant.

### Ethics statement.

No animals were used in this study. Porcine tissue is not subject to regulation, as it is a by-product of the slaughter of animals for food.

### Microarray data accession numbers.

The RNA-Seq data obtained in this study were submitted to ArrayExpress (accession no. E-MTAB-4047, E-MTAB-4049, and E-MTAB-4071).

## SUPPLEMENTAL MATERIAL

Figure S1  Relationships among 615 *A. baumannii* isolates based on multilocus sequence typing data (Pasteur scheme), as calculated by the BURST algorithm. Circles indicate major international groups responsible for the majority of diseases. Arrows indicate the strains used in this study. ATCC 19606 represents a singleton ST (ST52) and is not in this network. Download Figure S1, PDF file, 0.3 MB

Figure S2  Growth at 37°C, as determined by OD_600_. Download Figure S2, PDF file, 0.01 MB

Figure S3  A representative example of confirmation of MIC changes for AYEΔ*adeRS* and AYEΔ*adeB* by measurement of growth kinetics in the presence of tetracycline, as determined by OD_600_. Download Figure S3, PDF file, 0.2 MB

Figure S4  Adherent bacterial growth on mucosa, as determined by bacterial cell counts. (A) *adeRS* and *adeB* mutants of strain AYE. (B) *adeAB* mutant of S1. Download Figure S4, PDF file, 0.2 MB

Table S1  Gene expression data from the complete transcriptome analysis of *A. baumannii* AYE by RNA-Seq, showing differentially expressed genes (P < 0.05) in AYEΔ*adeRS* compared with AYE. Table S1, PDF file, 0.6 MB

Table S2  Gene expression data from the complete transcriptome analysis of *A. baumannii* AYE by RNA-Seq, showing differentially expressed genes (P < 0.05) in AYEΔ*adeB* compared with AYE. Table S2, PDF file, 1.1 MB

Table S3  Gene expression data from the complete transcriptome analysis of *A. baumannii* S1 by RNA-Seq, showing differentially expressed genes (P < 0.05) in AYEΔ*adeAB* compared with S1. Table S3, PDF file, 0.4 MB
