# Erratum for Richmond et al., The *Acinetobacter baumannii* Two-Component System AdeRS Regulates Genes Required for Multidrug Efflux, Biofilm Formation, and Virulence in a Strain-Specific Manner

**DOI:** 10.1128/mBio.00852-16

**Published:** 2016-06-21

**Authors:** Grace E. Richmond, Laura P. Evans, Michele J. Anderson, Matthew E. Wand, Laura C. Bonney, Alasdair Ivens, Kim Lee Chua, Mark A. Webber, J. Mark Sutton, Marnie L. Peterson, Laura J. V. Piddock

**Affiliations:** a Antimicrobial Agents Research Group, Institute of Microbiology and Infection, University of Birmingham, Edgbaston, Birmingham, United Kingdom; b Department of Experimental and Clinical Pharmacology, University of Minnesota, Minneapolis, Minnesota, USA; c Public Health England, Microbiology Services Division, Porton Down, Salisbury, United Kingdom; d Centre for Immunity, Infection and Evolution, University of Edinburgh, Edinburgh, United Kingdom; e Department of Biochemistry, Yong Loo Lin School of Medicine, National University of Singapore, Singapore

## ERRATUM

Volume 7, no. 2, doi:10.1128/mBio.00430-16, 2016. After careful review, it has come to our attention that the text in the abstract of our paper is at variance with one sentence in the article. During writing, revision, and editing, the sense was changed. The revised abstract below shows the correct text. We thank Maria Ramirez for bringing this to our attention.

The opportunistic pathogen *Acinetobacter baumannii* is able to persist in the environment and is often multidrug resistant (MDR), causing difficulties in the treatment of infections. Here, we show that the two-component system AdeRS, which regulates the production of the AdeABC multidrug resistance efflux pump, is required for the formation of a protective biofilm in an *ex vivo* porcine mucosal model, which mimics a natural infection of the human epithelium. Interestingly, deletion of *adeB* impacted on the ability to form a biofilm on plastic for strain AYE only and on the virulence in *Galleria mellonella* for Singapore strain 1 only. RNA-Seq revealed that loss of AdeRS or AdeB significantly altered the transcriptional landscape, resulting in the changed expression of many genes, notably those associated with antimicrobial resistance and virulence interactions. For example, *A. baumannii* lacking AdeRS displayed decreased expression of *adeABC* and a *pgaC*-like gene and increased expression of *pil* and *com* genes, whereas loss of AdeB resulted in decreased expression of *pil*, *com*, and ferric acinetobactin transport system genes. These data define the scope of AdeRS-mediated regulation, show that changes in the production of AdeABC mediate important phenotypes controlled by AdeRS, and suggest that AdeABC is a viable target for antimicrobial drug and anti-biofilm discovery.

