# Supplementary material for: The Acinetobacter baumannii Two-Component System AdeRS Regulates Genes Required for Multidrug Efflux, Biofilm Formation, and Virulence in a Strain-Specific Manner
Source: mBio. 2016 Apr 19;7(2):e00430-16. doi: 10.1128/mBio.00430-16 (PMC4850262; doi:10.1128/mBio.00430-16)

Fig S1 Relationships between 615 *A. baumannii* isolates based on MLST data (Pasteur scheme) as calculated by the BURST algorithm. Circles indicate major international groups responsible for the majority of disease. Arrows indicate strains used in this study. ATCC 19606 represents a singleton ST (ST 52) and is not on this network.

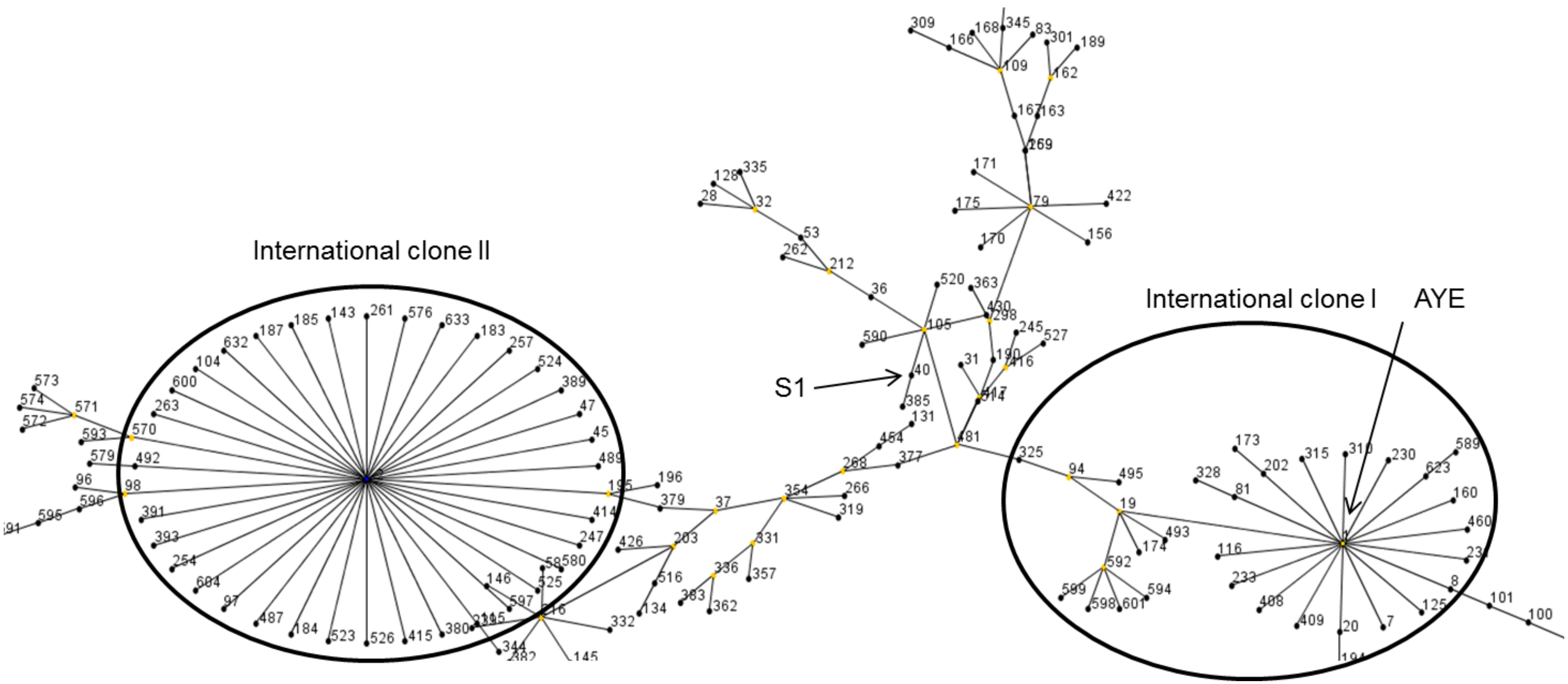

Supplement: Figure S1 — Relationships among 615 A. baumannii isolates based on multilocus sequence typing data (Pasteur scheme), as calculated by the BURST algorithm. Circles indicate major international groups responsible for the majority of diseases. Arrows indicate the strains used in this study. ATCC 19606 represents a singleton ST (ST52) and is not in this network. Download [file mbo002162774sf1.pdf]
