# Supplementary figures and images for: The Acinetobacter baumannii Two-Component System AdeRS Regulates Genes Required for Multidrug Efflux, Biofilm Formation, and Virulence in a Strain-Specific Manner
Source: mBio. 2016 Apr 19;7(2):e00430-16. doi: 10.1128/mBio.00430-16 (PMC4850262; doi:10.1128/mBio.00430-16)

**Figure S2.** Growth at 37°C, as determined by optical density.

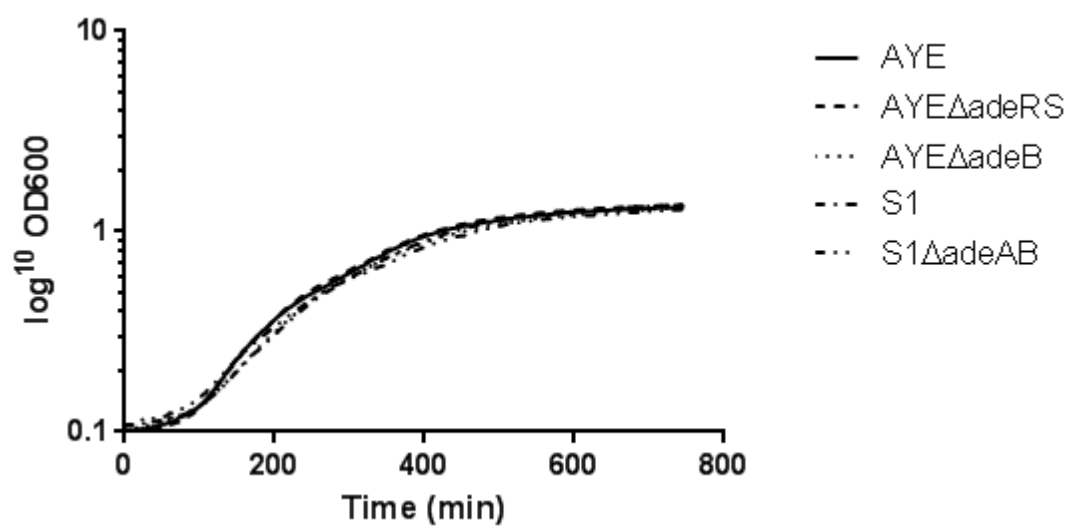

Supplement: Figure S2 — Growth at 37°C, as determined by OD600. Download [file mbo002162774sf2.pdf]
