# Supplementary material for: The Acinetobacter baumannii Two-Component System AdeRS Regulates Genes Required for Multidrug Efflux, Biofilm Formation, and Virulence in a Strain-Specific Manner
Source: mBio. 2016 Apr 19;7(2):e00430-16. doi: 10.1128/mBio.00430-16 (PMC4850262; doi:10.1128/mBio.00430-16)

Figure S3. A representative example of confirmation of MIC changes in AYE**Δ*adeRS* and AYEΔ*adeB* by measurement of growth kinetics in the presence of increasing concentrations of tetracycline, as determined by optical density.**

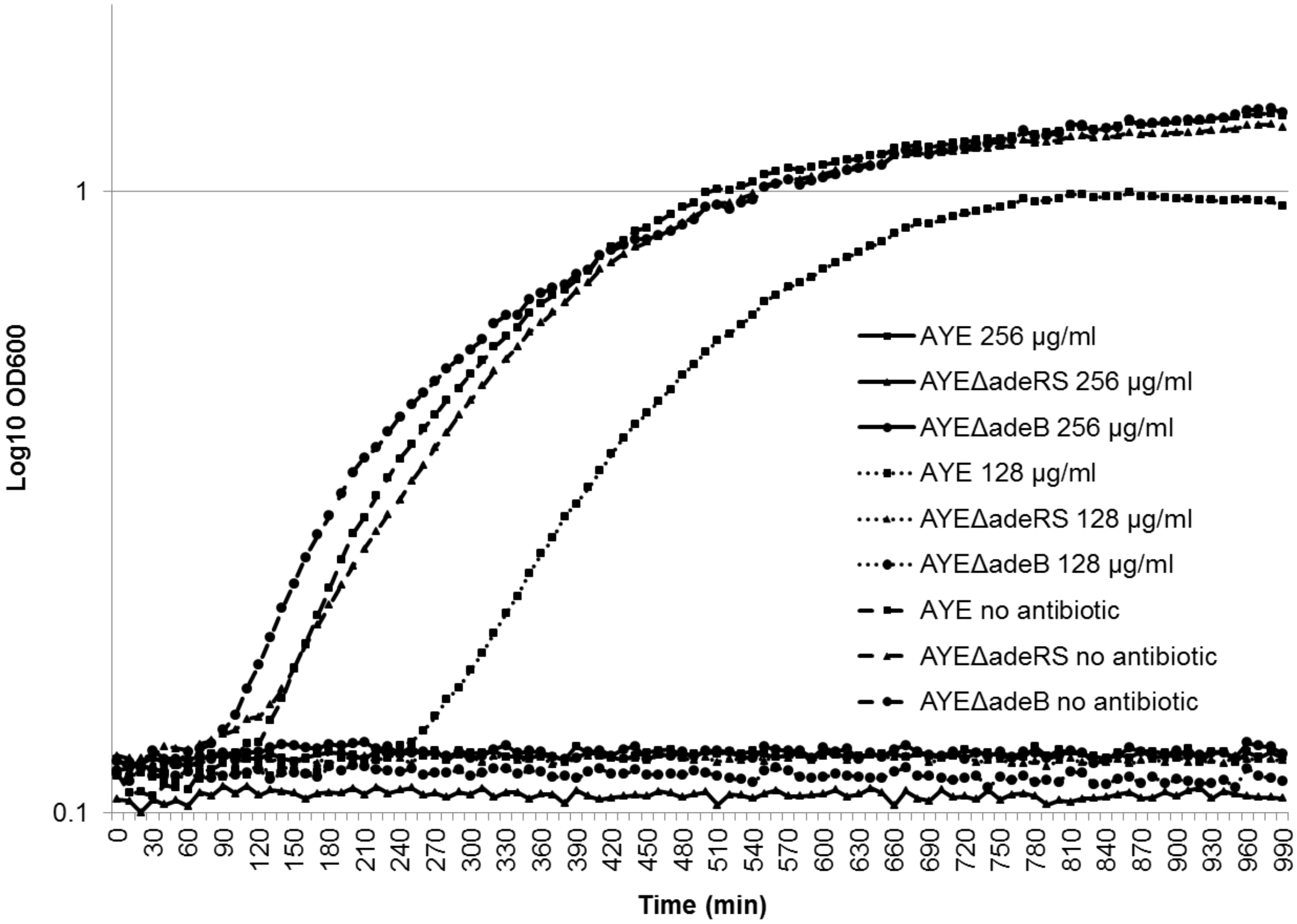

Supplement: Figure S3 — A representative example of confirmation of MIC changes for AYEΔadeRS and AYEΔadeB by measurement of growth kinetics in the presence of tetracycline, as determined by OD600. Download [file mbo002162774sf3.pdf]
