# Supplementary material for: The Acinetobacter baumannii Two-Component System AdeRS Regulates Genes Required for Multidrug Efflux, Biofilm Formation, and Virulence in a Strain-Specific Manner
Source: mBio. 2016 Apr 19;7(2):e00430-16. doi: 10.1128/mBio.00430-16 (PMC4850262; doi:10.1128/mBio.00430-16)

Figure S4. Adherent bacterial growth on mucosa , as determined by bacterial cell counts.

(A) AdeRS and AdeB mutants in AYE

(B) AdeAB mutant in S1

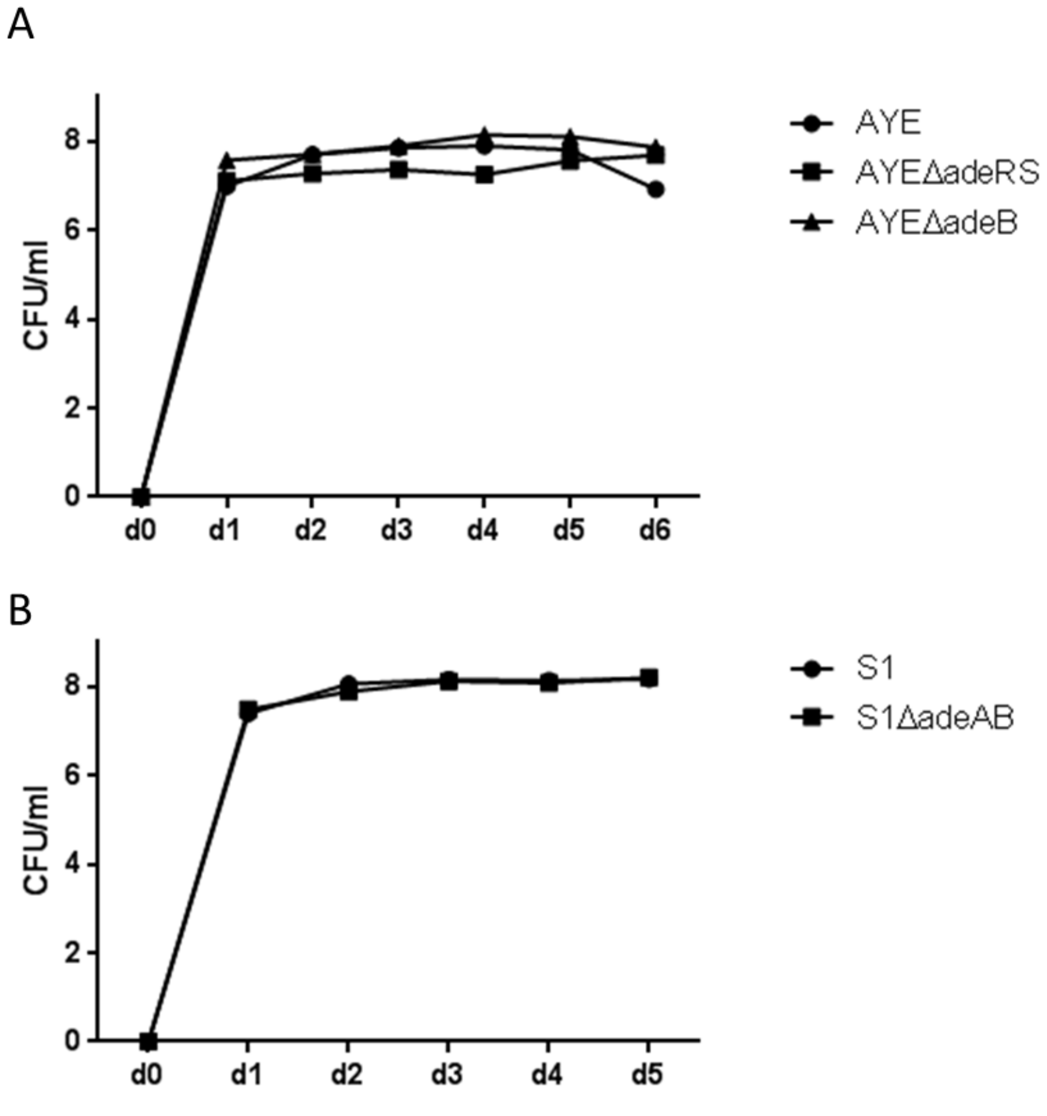

Supplement: Figure S4 — Adherent bacterial growth on mucosa, as determined by bacterial cell counts. (A) adeRS and adeB mutants of strain AYE. (B) adeAB mutant of S1. Download [file mbo002162774sf4.pdf]
