# Supplementary material for: The Acinetobacter baumannii Two-Component System AdeRS Regulates Genes Required for Multidrug Efflux, Biofilm Formation, and Virulence in a Strain-Specific Manner
Source: mBio. 2016 Apr 19;7(2):e00430-16. doi: 10.1128/mBio.00430-16 (PMC4850262; doi:10.1128/mBio.00430-16)
Supplement: Table S1 — Gene expression data from the complete transcriptome analysis of A. baumannii AYE by RNA-Seq, showing differentially expressed genes (P < 0.05) in AYEΔadeRS compared with AYE. [file mbo002162774st1.pdf]

Table S1 Gene expression data from the complete transcriptome analysis of *A. baumannii* AYE by RNA-Seq, showing differentially expressed genes (P < 0.05) in AYEΔ*adeRS* compared with AYE.

ABAYE Gene ID	Annotation	Gene Name	log2 Fold Change	Fold Change	P.Value
ABAYE1538	biotin carboxyl carrier protein of acetyl-CoA carboxylase (BCCP)	accB	0.66	1.58	0.01
ABAYE1877	Zn-dependent dipeptidase	acdP	-0.68	0.62	0.03
ABAYE3228	aconitate hydratase 1	acnA	-0.87	0.55	0.01
ABAYE3205	Copper-transporting P-type ATPase	actP	-0.85	0.55	0.02
ABAYE1821	membrane fusion protein	adeA	-7.00	0.01	0.00
ABAYE1822	RND protein	adeB	-6.50	0.01	0.00
ABAYE1823	outer membrane protein	adeC	-4.80	0.04	0.00
ABAYE1820	two-component response regulator	adeR	0.80	1.74	0.01
ABAYE1819	two-component sensor	adeS	-6.90	0.01	0.00
ABAYE2872	arginine/ornithine antiporter	arcD	0.56	1.47	0.04
ABAYE3839	N-alpha-acetylglutamate synthase (amino-acid acetyltransferase)	argA	0.54	1.45	0.03
ABAYE1539	3-dehydroquinate dehydratase%2C type II	aroQ	1.80	3.48	0.00
ABAYE3621	Rifampin ADP-ribosylating transferase ARR-2	arr-2	1.20	2.30	0.02

ABAYE3656	arsenate reductase (Arsenical pump modifier)	arsC	0.80	1.74	0.05
ABAYE3657	arsenical resistence operon repressor	arsR	1.10	2.14	0.02
ABAYE1921	aspartate ammonia-lyase (aspartase)	aspA	0.65	1.57	0.02
ABAYE2188	glutaminase-asparaginase	aspQ	1.90	3.73	0.01
ABAYE0353	arginine succinyltransferase	astA	1.60	3.03	0.03
ABAYE0355	succinylarginine dihydrolase	astB	0.85	1.80	0.03
ABAYE0354	succinylglutamic semialdehyde dehydrogenase	astD	0.95	1.93	0.01
ABAYE0356	succinylglutamate desuccinylase	astE	0.71	1.64	0.03
ABAYE3715	membrane-bound ATP synthase %2C F1 sector%2C epsilon-subunit	atpC	-0.96	0.51	0.04
ABAYE1096	bifunctional: 2%2C3-Dihydroxybenzoate-AMP ligase / S-dihydroxybenzoyltransferase	basE	0.63	1.55	0.05
ABAYE1097	2%2C3-dihydro-2%2C3-dihydroxybenzoate synthetase (isochorismatase) (N-terminal) with aryl carrier protein domain (C-terminal)	basF	0.67	1.59	0.04
ABAYE1090	Ferric acinetobactin transport system permease protein	bauC	0.75	1.68	0.01
ABAYE1909	3-hydroxybutyrate dehydrogenase	bdhA	-1.10	0.47	0.03
ABAYE0307	bacterioferritin	bfrB	-1.80	0.29	0.05
ABAYE2129	biotin synthetase	bioB	0.76	1.69	0.01
ABAYE3001	dethiobiotin synthetase (Dethiobiotin synthase) (DTB synthetase) (DTBS)	bioD	0.52	1.43	0.05

ABAYE0800	carbamoyl-phosphate synthase%2C large subunit	carB	-0.58	0.67	0.05
ABAYE1719	muconolactone delta-isomerase (MIase)	catC	1.10	2.14	0.01
ABAYE3411	curved DNA-binding protein	cbpA	-0.62	0.65	0.05
ABAYE2787	citrate transporter	citN	1.30	2.46	0.03
ABAYE3288	ATP-dependent Clp protease proteolytic subunit (Endopeptidase Clp) (Caseinolytic protease) (Protease Ti) (Heat shock protein F21.5)	clpP	0.72	1.65	0.05
ABAYE3620	Chloramphenicol resistance protein	cmlA	0.64	1.56	0.03
ABAYE3447	dephosphocoenzyme A kinase	coaE	0.77	1.71	0.01
ABAYE0316	pilin like competence factor	comB	2.60	6.06	0.01
ABAYE0319	Pilin like competence factor	comE	1.30	2.46	0.01
ABAYE0320	pilin like competence factor	comF	1.50	2.83	0.02
ABAYE0143	catabolite repression control protein	crc	0.72	1.65	0.02
ABAYE2535	cold shock-like protein	csp	-2.10	0.23	0.04
ABAYE0930	sulfate transport protein (ABC superfamily%2C peri_bind)	cysP	2.20	4.59	0.04
ABAYE1567	D-amino acid dehydrogenase%2C small subunit	dadA	-0.81	0.57	0.02
ABAYE3774	D-amino acid dehydrogenase%2C small subunit	dadA	0.97	1.96	0.01
ABAYE2315	acyl-CoA dehydrogenase	dcaA	1.10	2.14	0.04
ABAYE2307	beta-ketoadipyl CoA thiolase	dcaF	1.20	2.30	0.01

ABAYE2306	dehydrogenase	dcaH	1.30	2.46	0.03
ABAYE2301	acyl-CoA-transferase subunit A	dcaI	1.80	3.48	0.01
ABAYE1730	Dihydrocoumarin hydrolase	dch	-1.10	0.47	0.03
ABAYE2265	fragment of RecA-dependent DNA damage-inducible protein (partial)	ddrR	-1.30	0.41	0.01
ABAYE2833	peptide deformylase 2	def2	0.88	1.84	0.02
ABAYE1343	delta 9 acyl-lipid fatty acid desaturase	desC	-0.68	0.62	0.02
ABAYE3796	D-lactate dehydrogenase%2C NADH independent%2C FAD-binding domain	dld	0.71	1.64	0.02
ABAYE3833	thiol:disulfide interchange protein%2C periplasmic%2C alkali-inducible	dsbA	1.60	3.03	0.05
ABAYE2929	deoxyuridine 5-triphosphate nucleotidohydrolase	dut	1.40	2.64	0.03
ABAYE3569	ethidium bromide resistance protein (E1 protein)	ebr	0.71	1.64	0.01
ABAYE3613	ethidium bromide resistance protein (E1 protein)	ebr	0.65	1.57	0.02
ABAYE3617	ethidium bromide resistance protein (E1 protein)	ebr	0.63	1.55	0.02
ABAYE3642	ethidium bromide resistance protein (E1 protein)	ebr	0.65	1.57	0.02
ABAYE0677	bacteriolytic lipoprotein entericidin B.	ecnB	-3.00	0.13	0.03
ABAYE2951	beta-ketoacyl-ACP synthase I (3-oxoacyl-[acyl-carrier-protein] synthase I)	fabB	0.96	1.95	0.04
ABAYE3250	NADH-dependent enoyl-ACP reductase	fabI	0.78	1.72	0.02
ABAYE0436	acyl coenzyme A dehydrogenase	fadE	1.00	2.00	0.03

ABAYE2479	7-Fe ferredoxin	fdxA	-0.71	0.61	0.03
ABAYE3315	dihydrofolate reductase	folA	-0.59	0.66	0.04
ABAYE3284	fumarate hydratase	fumA	-0.62	0.65	0.04
ABAYE1563	fumarase C (fumarate hydratase Class II)	fumC	-0.93	0.52	0.01
ABAYE2920	negative regulator of ferric iron uptake	fur	-0.60	0.66	0.04
ABAYE0210	NADP+-dependent succinate semialdehyde dehydrogenase	gabD	-0.92	0.53	0.02
ABAYE0633	glucose dehydrogenase [pyrroloquinoline-quinone] precursor (Quinoprotein glucose DH)	gcd	-0.68	0.62	0.04
ABAYE0351	glutamate dehydrogenase (NAD(P)+) oxidoreductase protein	gdh	0.76	1.69	0.02
ABAYE3549	regulatory protein%2C P-II 2%2C for nitrogen assimilation by glutamine synthetase%2C regulates GlnL (NRII) and GlnE (ATase)	glnK	0.87	1.83	0.04
ABAYE3279	high-affinity gluconate permease (GntP family)	gntT	0.90	1.87	0.05
ABAYE0799	transcription elongation factor%2C cleaves 3 nucleotide of paused mRNA	greA	0.87	1.83	0.05
ABAYE2631	acyl coenzyme A dehydrogenase	hcaD	1.50	2.83	0.00
ABAYE2632	porin	hcaE	0.58	1.49	0.05
ABAYE3507	uroporphyrinogen-III synthase (UROS) (Uroporphyrinogen-III cosynthetase) (Hydroxymethylbilane hydrolyase [cyclizing])	hemD	0.54	1.45	0.04
ABAYE3158	integration host factor (IHF)%2C alpha subunit%2C DNA-binding protein%2C DNA replication	himA	-0.91	0.53	0.00

ABAYE1179	Histidine transport system permease protein (ABC superfamily%2C membrane)	hisM	0.65	1.57	0.03
ABAYE3238	acetohydroxy acid isomeroreductase	ilvC	0.77	1.71	0.03
ABAYE3239	acetolactate synthase isozyme III%2C small subunit	ilvH	0.56	1.47	0.05
ABAYE2022	iron-binding protein believed to be involved in Fe-S protein formation or repair%3B function as a scaffold for the assembly of the transiently bound Fe/S cluster	iscU	-0.77	0.59	0.04
ABAYE2270	Catalase hydroperoxidase II	katE	-2.00	0.25	0.00
ABAYE0326	lipase chaperone (Lipase foldase) (Lipase helper protein) (Lipase activator protein) (Lipase modulator)	lifO	1.10	2.14	0.04
ABAYE3797	L-lactate dehydrogenase%2C FMN linked	lldD	0.97	1.96	0.04
ABAYE3775	transcriptional regulator for lrp regulon and high-affinity branched-chain amino acid transport system%2C mediator of leucine response (AsnC family)	lrp	1.30	2.46	0.02
ABAYE3853	prolipoprotein signal peptidase (Signal peptidase II.)	lspA	0.67	1.59	0.03
ABAYE2233	malonate decarboxylase%2C alpha subunit	mdcA	0.69	1.61	0.03
ABAYE2231	malonate decarboxylase%2C delta subunit	mdcC	1.10	2.14	0.02
ABAYE2230	malonate decarboxylase%2C beta subunit	mdcD	0.80	1.74	0.03
ABAYE3605	Mercuric reductase (Hg(II) reductase)	merA	-0.98	0.51	0.03
ABAYE1448	delta(2)-isopentenylpyrophosphate tRNA-adenosine transferase	miaA	-1.30	0.41	0.00
ABAYE2936	cell division topological specificity factor (reverses minC inhibition of	minE	-0.64	0.64	0.02

	FtsZ ring)				
ABAYE0703	rod shape-determining protein	mreD	0.91	1.88	0.02
ABAYE0647	mechanosensitive channel	mscL	-1.00	0.50	0.00
ABAYE0183	NADH-dependent FMN reductase	msuE	1.40	2.64	0.02
ABAYE3095	cis%2Ccis-muconate transport protein (MFS superfamily)	mucK	1.20	2.30	0.02
ABAYE1542	response regulator protein	nasT	-1.60	0.33	0.02
ABAYE2766	endonuclease III DNA glycosylase/apyrimidinic (AP) lyase	nth	-0.67	0.63	0.03
ABAYE3059	NADH dehydrogenase I chain B	nuoB	0.73	1.66	0.02
ABAYE3007	trehalose-6-phosphate synthase	otsA	-2.50	0.18	0.01
ABAYE2375	subunit of Phenylacetate-CoA oxygenase%2C phenylacetic acid degradation	paaA	3.20	9.19	0.05
ABAYE2367	beta-ketoadipyl CoA thiolase with thiolase-like domain%2C phenylacetic acid degradation	paaJ	2.60	6.06	0.04
ABAYE0901	peptidoglycan-associated lipoprotein precursor	pal	-0.69	0.62	0.05
ABAYE3532	D-alanyl-D-alanine endopeptidase%2C penicillin-binding protein 7 and penicillin-binding protein 8	pbpG	-0.88	0.54	0.02
ABAYE0818	phosphoenolpyruvate carboxykinase [GTP] (PEP carboxykinase) (PEPCK)	pckG	-0.70	0.62	0.03
ABAYE3111	copper resistance protein A precursor	pcoA	-0.78	0.58	0.05
ABAYE3444	type 4 fimbrial biogenesis protein	pilB	2.20	4.59	0.00

ABAYE3445	type 4 fimbrial assembly protein	pilC	1.80	3.48	0.01
ABAYE3446	type 4 prepilin-like proteins leader peptide processing enzyme (Protein secretion protein XCPA)[Includes: Leader peptidase (Prepilin peptidase)%3B N-methyltransferase]	pilD	1.50	2.83	0.00
ABAYE0667	twitching motility protein	pilG	2.10	4.29	0.01
ABAYE0668	twitching motility protein	pilH	1.30	2.46	0.04
ABAYE0669	twitching motility protein	pilI	2.40	5.28	0.01
ABAYE0670	type IV pilus biogenesis protein	pilJ	2.30	4.92	0.00
ABAYE3535	type 4 fimbriae expression regulatory protein	pilR	1.50	2.83	0.01
ABAYE2918	twitching motility protein	pilT	3.00	8.00	0.00
ABAYE2919	twitching motility protein	pilU	2.90	7.46	0.01
ABAYE2074	type 4 fimbrial biogenesis protein	pilZ	0.74	1.67	0.04
ABAYE3675	inorganic pyrophosphatase	ppa	0.97	1.96	0.03
ABAYE3232	peptide chain release factor 3	prfC	-0.80	0.57	0.05
ABAYE3793	methylisocitrate lyase	prpB	2.00	4.00	0.02
ABAYE3792	methylcitrate synthase (citrate synthase 2)	prpC	0.62	1.54	0.04
ABAYE3283	phosphate acetyltransferase	pta	1.30	2.46	0.02
ABAYE0095	6%2C7-dimethyl-8-ribityllumazine synthase (Lumazine synthase)(riboflavin synthase beta chain)	ribH	0.57	1.48	0.02

ABAYE3902	RNase P%2C protein C5 component%2C processes tRNA%2C 4.5S RNA	rnpA	-1.30	0.41	0.02
ABAYE1650	ribose 5-phosphate isomerase	rpiA	-0.83	0.56	0.03
ABAYE0408	50S ribosomal protein L3	rplC	0.55	1.46	0.03
ABAYE1382	50S ribosomal protein L9	rplI	-1.20	0.44	0.01
ABAYE0324	50S ribosomal protein L19	rplS	-0.50	0.71	0.04
ABAYE3326	50S ribosomal protein L33	rpmG	-2.10	0.23	0.00
ABAYE3164	50S ribosomal protein L35	rpmI	-1.30	0.41	0.03
ABAYE1286	30S ribosomal protein S21	rpsU	0.87	1.83	0.02
ABAYE2798	rubredoxin	rubA	-0.68	0.62	0.03
ABAYE0774	succinate dehydrogenase%2C cytochrome b556 subunit	sdhC	0.80	1.74	0.01
ABAYE3442	preprotein translocase IISP family%2C auxillary membrane component	secG	0.63	1.55	0.05
ABAYE0332	D-3-phosphoglycerate dehydrogenase	serA	0.81	1.75	0.05
ABAYE3399	small ubiquitous protein required for normal growth	sirA	-0.81	0.57	0.01
ABAYE2921	outer membrane lipoprotein	smpA	-0.76	0.59	0.04
ABAYE1356	L-sorbosone dehydrogenase	sndH	-0.63	0.65	0.04
ABAYE1134	superoxide dismutase [Fe]	sodB	-0.81	0.57	0.02
ABAYE3841	alkanesulfonate transport protein (ABC superfamily%2C peri_bind)	ssuA	1.00	2.00	0.02

ABAYE3107	suppressor of gro	sugE	1.20	2.30	0.00
ABAYE0382	inositol-1-monophosphatase (IMPase) (Inositol-1-phosphatase) (I-1-Pase)	suhB	-1.00	0.50	0.01
ABAYE3637	Tetracycline resistance protein%2C class G (TETA(G))	tetA	1.50	2.83	0.02
ABAYE1283	2-keto-D-gluconate reductase (2-ketoaldonate reductase)	tkrA	1.10	2.14	0.03
ABAYE0387	toluene tolerance efflux transporter (ABC superfamily%2C peri-bind)	ttg2C	-0.81	0.57	0.04
ABAYE1710	Copper amine oxidase precursor (Tyramine oxidase) (2-phenylethylamine oxidase)	tynA	1.40	2.64	0.04
ABAYE3795	tyrosine aminotransferase%2C tyrosine repressible%2C PLP-dependent	tyrB	0.89	1.85	0.05
ABAYE0716	undecaprenylpyrophosphate phosphatase (bacitracin resistance protein)	uppP	-0.92	0.53	0.01
ABAYE2778	urease gamma subunit	ureA	1.80	3.48	0.01
ABAYE2623	vanillate O-demethylase oxygenase subunit (4-hydroxy-3-methoxybenzoate demethylase)	vanA	1.40	2.64	0.04
ABAYE2588	cyclic AMP receptor protein	vfr	1.30	2.46	0.02
ABAYE0969	UDP-N-acetyl glucosamine-2-epimerase	wecB	-0.80	0.57	0.01
ABAYE_16s_1	ABAYE_16s_1		-4.80	0.04	0.03
ABAYE_16s_2	ABAYE_16s_2		-4.50	0.04	0.04
ABAYE_16s_3	ABAYE_16s_3		-4.70	0.04	0.03

ABAYE_16s_4	ABAYE_16s_4		-4.60	0.04	0.05
ABAYE_16s_5	ABAYE_16s_5		-4.90	0.03	0.03
ABAYE_16s_6	ABAYE_16s_6		-4.80	0.04	0.03
ABAYE_23s_1	ABAYE_23s_1		-4.40	0.05	0.03
ABAYE_23s_2	ABAYE_23s_2		-4.40	0.05	0.03
ABAYE_23s_3	ABAYE_23s_3		-4.30	0.05	0.03
ABAYE_23s_4	ABAYE_23s_4		-4.40	0.05	0.02
ABAYE_23s_5	ABAYE_23s_5		-4.40	0.05	0.02
ABAYE_23s_6	ABAYE_23s_6		-4.40	0.05	0.02
ABAYE_5s_1	ABAYE_5s_1		-0.87	0.55	0.04
ABAYE_5s_2	ABAYE_5s_2		-0.96	0.51	0.02
ABAYE_5s_4	ABAYE_5s_4		-1.10	0.47	0.00
ABAYE_5s_6	ABAYE_5s_6		-0.99	0.50	0.01
ABAYE0011	putative chaperone involved in Fe-S cluster assembly and activation (HesB-like)		-0.91	0.53	0.05
ABAYE0016	putative glutathione S-transferase		0.92	1.89	0.01
ABAYE0017	conserved hypothetical protein%3B putative exported protein		1.10	2.14	0.03
ABAYE0020	conserved hypothetical protein%3B putative signal peptide		-1.00	0.50	0.04

ABAYE0050	conserved hypothetical protein%3B putative exported protein		-2.20	0.22	0.03
ABAYE0111	putative aminotransferase		-0.48	0.72	0.05
ABAYE0112	conserved hypothetical protein		-0.65	0.64	0.04
ABAYE0113	conserved hypothetical protein%3B putative exported protein		-0.63	0.65	0.04
ABAYE0120	hypothetical protein%3B putative signal peptide		0.67	1.59	0.03
ABAYE0123	hypothetical protein%3B putative signal peptide		0.98	1.97	0.02
ABAYE0130	conserved hypothetical protein%3B putative exported protein		1.10	2.14	0.01
ABAYE0137	conserved hypothetical protein%3B putative exported protein		0.47	1.39	0.04
ABAYE0141	conserved hypothetical protein%3B putative exported protein		-1.50	0.35	0.00
ABAYE0142	conserved hypothetical protein		0.67	1.59	0.04
ABAYE0164	conserved hypothetical protein%3B putative enzyme		1.90	3.73	0.00
ABAYE0165	putative ferredoxin		1.30	2.46	0.02
ABAYE0170	conserved hypothetical protein%3B putative exported protein		-0.95	0.52	0.01
ABAYE0180	putative isochorismatase		1.20	2.30	0.00
ABAYE0187	conserved hypothetical protein		2.40	5.28	0.03
ABAYE0188	putative sodium:solute symporter		2.60	6.06	0.04
ABAYE0190	putative high affinity choline transport protein (Bet-like)		-0.57	0.67	0.04
ABAYE0196	putative transcriptional regulator (TetR/AcrR family)		0.55	1.46	0.05

ABAYE0219	conserved hypothetical protein%3B putative membrane protein		0.63	1.55	0.03
ABAYE0224	hypothetical protein		-1.60	0.33	0.02
ABAYE0226	conserved hypothetical protein		1.30	2.46	0.02
ABAYE0228	hypothetical protein		-0.66	0.63	0.04
ABAYE0237	putative Efflux protein%2C LysE family		0.61	1.53	0.03
ABAYE0244	putative homoserine kinase (ThrB)		0.72	1.65	0.03
ABAYE0247	putative membrane protein		0.61	1.53	0.02
ABAYE0262	putative sulfate permease		-1.30	0.41	0.01
ABAYE0266	conserved hypothetical protein%3B putative signal peptide		2.30	4.92	0.01
ABAYE0268	putative Component of the czc cation-efflux system (CzcI)		0.85	1.80	0.01
ABAYE0286	conserved hypothetical protein		2.00	4.00	0.04
ABAYE0290	putative membrane protein (ComM)		1.90	3.73	0.00
ABAYE0291	putative membrane protein (ComN)		2.90	7.46	0.02
ABAYE0292	putative membrane protein (ComO)		2.50	5.66	0.00
ABAYE0293	putative lipoprotein (ComL)		3.30	9.85	0.00
ABAYE0294	putative outer membrane protein (ComQ)		2.50	5.66	0.00
ABAYE0300	conserved hypothetical protein		-0.66	0.63	0.03
ABAYE0301	conserved hypothetical protein%3B putative exported protein		-0.71	0.61	0.04

ABAYE0302	conserved hypothetical protein%3B putative membrane protein		-0.85	0.55	0.01
ABAYE0303	conserved hypothetical protein		-1.20	0.44	0.01
ABAYE0304	putative fimbrial protein precursor (Pilin)		3.40	10.56	0.04
ABAYE0314	putative Fimbrial protein precursor%3B putative type IV pilin protein		1.90	3.73	0.02
ABAYE0315	putative type IV fimbrial biogenesis protein		2.90	7.46	0.01
ABAYE0317	putative type IV fimbrial biogenesis protein		2.80	6.96	0.01
ABAYE0318	putative competence factor involved in DNA binding and uptake (ComC)		1.70	3.25	0.01
ABAYE0329	conserved hypothetical protein		-1.10	0.47	0.05
ABAYE0330	conserved hypothetical protein%3B putative membrane protein		-1.10	0.47	0.01
ABAYE0339	conserved hypothetical protein%3B putative membrane protein		0.50	1.41	0.04
ABAYE0358	conserved hypothetical protein%3B putative signal peptide		-0.67	0.63	0.03
ABAYE0370	conserved hypothetical protein		0.52	1.43	0.05
ABAYE0403	putative flavohemoprotein (Hemoglobin-like protein) (Flavohemoglobin)		1.10	2.14	0.01
ABAYE0438	conserved hypothetical protein		0.79	1.73	0.04
ABAYE0441	putative integral membrane protein%2C transporter		0.85	1.80	0.02
ABAYE0459	conserved hypothetical protein%3B putative tonB-domain		0.68	1.60	0.02
ABAYE0466	conserved hypothetical protein		-1.00	0.50	0.01
ABAYE0471	conserved hypothetical protein		-1.40	0.38	0.03

ABAYE0493	conserved hypothetical protein%3B putative membrane protein		0.62	1.54	0.02
ABAYE0506	conserved hypothetical protein		1.70	3.25	0.00
ABAYE0518	conserved hypothetical protein%3B putative exported protein		-0.98	0.51	0.04
ABAYE0529	conserved hypothetical protein		0.90	1.87	0.02
ABAYE0533	hypothetical protein		-0.69	0.62	0.01
ABAYE0534	hypothetical protein		0.57	1.48	0.03
ABAYE0538	hypothetical protein		0.75	1.68	0.03
ABAYE0542	hypothetical protein		1.00	2.00	0.00
ABAYE0543	conserved hypothetical protein%3B Putative phage-related DNA-binding protein		-1.30	0.41	0.00
ABAYE0550	conserved hypothetical protein%3B putative phage related protein		0.92	1.89	0.01
ABAYE0557	phage-related major tail sheath protein (FI-like)		1.30	2.46	0.02
ABAYE0566	putative phage-related membrane protein		0.91	1.88	0.01
ABAYE0567	putative phage-related membrane protein		1.20	2.30	0.01
ABAYE0587	putative glycosyl transferase		-1.40	0.38	0.04
ABAYE0591	putative TonB-dependent receptor protein		0.46	1.38	0.05
ABAYE0605	putative cobalamin adenosyltransferase		0.57	1.48	0.02
ABAYE0636	putative multidrug transporter		-1.40	0.38	0.00

ABAYE0639	putative type IV fimbrial biogenesis protein		0.69	1.61	0.02
ABAYE0662	conserved hypothetical protein		-0.98	0.51	0.03
ABAYE0666	conserved hypothetical protein%3B putative exported protein		1.80	3.48	0.00
ABAYE0671	putative sensor histidine kinase/response regulator%3B putative protein of chemotactic signal transduction system (ChA-like)		2.20	4.59	0.00
ABAYE0672	conserved hypothetical protein		1.10	2.14	0.01
ABAYE0686	putative nitroreductase		1.10	2.14	0.00
ABAYE0689	putative amino-acid transport protein		0.98	1.97	0.04
ABAYE0707	conserved hypothetical protein		-0.94	0.52	0.01
ABAYE0711	putative long-chain fatty acid transport protein		1.10	2.14	0.01
ABAYE0725	conserved hypothetical protein		0.74	1.67	0.04
ABAYE0730	conserved hypothetical protein%3B putative protein (DcaP-like)		1.20	2.30	0.04
ABAYE0742	putative oxidoreductase/dehydrogenase		-0.53	0.69	0.05
ABAYE0749	putative phosphatidylglycerophosphatase B (PgpB)		-0.87	0.55	0.04
ABAYE0760	conserved hypothetical protein		0.61	1.53	0.04
ABAYE0792	conserved hypothetical protein%3B putative biofilm-associated protein with RTX toxins and related Ca2+-binding domain		-1.40	0.38	0.00
ABAYE0797	putative universal stress protein A (UspA)		-1.00	0.50	0.03
ABAYE0832	conserved hypothetical protein		-0.64	0.64	0.05

ABAYE0841	conserved hypothetical protein		0.92	1.89	0.04
ABAYE0869	conserved hypothetical protein%3B putative membrane protein		-0.90	0.54	0.02
ABAYE0919	conserved hypothetical protein		1.50	2.83	0.00
ABAYE0920	conserved hypothetical protein%3B putative exported protein		-0.59	0.66	0.04
ABAYE0921	conserved hypothetical protein%3B putative exported protein		0.72	1.65	0.01
ABAYE0952	conserved hypothetical protein		-1.60	0.33	0.03
ABAYE0971	conserved hypothetical protein%3B putative exported protein		-1.20	0.44	0.03
ABAYE0972	putative glycosyltransferase		-1.20	0.44	0.01
ABAYE0973	conserved hypothetical protein		-0.70	0.62	0.03
ABAYE0974	conserved hypothetical protein%3B putative membrane protein		-1.20	0.44	0.01
ABAYE0989	conserved hypothetical protein		-0.57	0.67	0.03
ABAYE0995	conserved hypothetical protein		0.72	1.65	0.02
ABAYE0997	conserved hypothetical protein		0.58	1.49	0.04
ABAYE1029	putative transcriptional regulator (AsnC family)		1.30	2.46	0.04
ABAYE1046	conserved hypothetical protein%3B putative signal peptide		0.84	1.79	0.02
ABAYE1055	conserved hypothetical protein		0.81	1.75	0.02
ABAYE1063	putative Choline/carnitine/betaine transporter family protein		-0.64	0.64	0.04
ABAYE1072	putative Inner membrane protein%3B putative permease of the drug/metabolite		0.93	1.91	0.02

	transporter (DMT) superfamily			
ABAYE1084	conserved hypothetical protein	1.30	2.46	0.02
ABAYE1087	putative non-ribosomal peptide synthetase with condensation and peptidyl carrier protein domains (basB)	0.67	1.59	0.01
ABAYE1124	putative peptidase%3B putative metallopeptidase	0.85	1.80	0.03
ABAYE1135	conserved hypothetical protein	0.75	1.68	0.01
ABAYE1145	putative acyl-CoA dehydrogenase	-0.88	0.54	0.04
ABAYE1148	conserved hypothetical protein%3B putative signal pepetide	-0.82	0.57	0.02
ABAYE1160	putative rare lipoprotein A family (RlpA-like)	-0.92	0.53	0.03
ABAYE1167	conserved hypothetical protein	-0.93	0.52	0.02
ABAYE1189	fragment of conserved hypothetical protein (part 2)	-0.80	0.57	0.02
ABAYE1191	putative secretion pathway ATPase	1.50	2.83	0.00
ABAYE1201	conserved hypothetical protein	-0.87	0.55	0.04
ABAYE1207	bifunctional protein [Includes: putative prephenate or cyclohexadienyl dehydrogenase%3B 3-phosphoshikimate 1-carboxyvinyltransferase (5-enolpyruvylshikimate-3-phosphate synthase) (EPSP synthase) (EPSPS) (AroA)]	0.48	1.39	0.05
ABAYE1219	putative cold shock protein	-1.30	0.41	0.01
ABAYE1226	conserved hypothetical protein	-1.20	0.44	0.05
ABAYE1228	hypothetical protein	-0.67	0.63	0.03

ABAYE1229	hypothetical protein%3B putative phage related protein		-0.83	0.56	0.03
ABAYE1230	conserved hypothetical protein		-0.93	0.52	0.04
ABAYE1256	conserved hypothetical protein%3B putative phage related protein		-0.71	0.61	0.04
ABAYE1298	conserved hypothetical protein		-3.40	0.09	0.01
ABAYE1301	putative methyltransferase		-1.50	0.35	0.01
ABAYE1303	conserved hypothetical protein%3B putative signal peptide		-1.30	0.41	0.01
ABAYE1313	putative Short-chain dehydrogenase/reductase		1.00	2.00	0.01
ABAYE1327	conserved hypothetical protein		2.00	4.00	0.00
ABAYE1345	conserved hypothetical protein		0.87	1.83	0.04
ABAYE1347	conserved hypothetical protein%3B putative membrane protein		0.90	1.87	0.05
ABAYE1351	conserved hypothetical protein%3B putative exported protein		1.90	3.73	0.00
ABAYE1355	putative membrane protein		-0.83	0.56	0.03
ABAYE1372	fragment of conserved hypothetical protein (partial)		-2.30	0.20	0.00
ABAYE1376	conserved hypothetical protein		-0.93	0.52	0.00
ABAYE1379	conserved hypothetical protein		-0.85	0.55	0.02
ABAYE1395	hypothetical protein%3B putative biofilm synthesis protein		-1.20	0.44	0.04
ABAYE1396	Biofilm synthesis N-glycosyltransferase (PgaC-like)		-1.10	0.47	0.05
ABAYE1397	hypothetical protein%3B putative biofilm synthesis protein		-0.69	0.62	0.05

ABAYE1409	putative binding-protein-dependent transport systems periplasmic component		0.81	1.75	0.04
ABAYE1426	conserved hypothetical protein		-0.86	0.55	0.05
ABAYE1429	conserved hypothetical protein%3B putative exported protein		-0.81	0.57	0.02
ABAYE1430	conserved hypothetical protein		-0.74	0.60	0.02
ABAYE1443	putative acetyltransferase		-1.10	0.47	0.02
ABAYE1459	putative transport protein		-1.00	0.50	0.01
ABAYE1468	conserved hypothetical protein		-2.10	0.23	0.00
ABAYE1470	conserved hypothetical protein%3B putative biofilm synthesis protein		-1.50	0.35	0.03
ABAYE1472	putative Fimbrial usher protein		-1.50	0.35	0.01
ABAYE1473	hypothetical protein%3B putative biofilm synthesis domain		-1.10	0.47	0.03
ABAYE1474	putative glutathione S-transferase		-1.30	0.41	0.00
ABAYE1475	putative short chain dehydrogenase		-1.10	0.47	0.01
ABAYE1476	putative oxidoreductase%2C short-chain dehydrogenase/reductase family		-0.68	0.62	0.02
ABAYE1480	hypothetical protein%3B putative signal peptide		1.50	2.83	0.03
ABAYE1485	putative transcriptional regulator (TetR family)		-1.20	0.44	0.04
ABAYE1488	hypothetical protein		-2.30	0.20	0.00
ABAYE1506	putative transporter transmembrane protein (magnesium transporter)		1.30	2.46	0.02

ABAYE1561	putative intracellular sulfur oxidation protein (DsrE-like)		-1.80	0.29	0.00
ABAYE1562	putative UDP-galactose 4-epimerase (GalE-like)		-1.00	0.50	0.02
ABAYE1584	putative outer membrane protein (OmpH)		-0.70	0.62	0.01
ABAYE1596	hypothetical protein		-1.10	0.47	0.01
ABAYE1599	L-kynurenine hydrolase		0.80	1.74	0.04
ABAYE1604	conserved hypothetical protein		-1.20	0.44	0.02
ABAYE1607	Putative diguanylate cyclase/phosphodiesterase		-0.94	0.52	0.02
ABAYE1611	conserved hypothetical protein%3B putative signal peptide		1.60	3.03	0.01
ABAYE1629	conserved hypothetical protein		-0.83	0.56	0.02
ABAYE1634	conserved hypothetical protein%3B putative exported protein		-0.78	0.58	0.04
ABAYE1648	putative potassium uptake protein		0.61	1.53	0.04
ABAYE1671	putative cell division protein (FtsB-like)		-0.99	0.50	0.04
ABAYE1688	conserved hypothetical protein%3B putative membrane protein		0.79	1.73	0.03
ABAYE1703	conserved hypothetical protein		1.60	3.03	0.04
ABAYE1705	putative Aromatic-ring-hydroxylating dioxygenase small subunit		1.00	2.00	0.02
ABAYE1706	putative Short-chain dehydrogenase/reductase		0.72	1.65	0.04
ABAYE1711	conserved hypothetical protein%3B putative signal peptide		1.30	2.46	0.05
ABAYE1713	putative Penicillin acylase precursor (Penicillin amidase) (Penicillin		-0.67	0.63	0.05

	amidohydrolase)				
ABAYE1725	putative Permease of the major facilitator		0.79	1.73	0.03
ABAYE1728	putative NADP-dependent aldehyde dehydrogenase (AldH-like)		0.96	1.95	0.02
ABAYE1735	conserved hypothetical protein		-1.00	0.50	0.01
ABAYE1736	putative oxidoreductase%3B putative Metal-binding Oxidoreductase		-1.10	0.47	0.01
ABAYE1748	conserved hypothetical protein		-1.40	0.38	0.01
ABAYE1749	putative Isochorismatase hydrolase		-0.77	0.59	0.01
ABAYE1772	conserved hypothetical protein%3B putative regucalcin family protein		-0.53	0.69	0.04
ABAYE1780	putative NADP-dependent aldehyde dehydrogenase (AldH-like)		1.50	2.83	0.02
ABAYE1781	putative Dihydroxy-acid and 6-phosphogluconate dehydratase (IlvD-Edd-like)		1.60	3.03	0.01
ABAYE1783	putative transcriptional regulator (LysR family)		1.40	2.64	0.04
ABAYE1785	putative Permease of the major facilitator%3B putative tartrate transporter		0.77	1.71	0.04
ABAYE1796	putative multidrug resistance efflux pump		-0.60	0.66	0.05
ABAYE1807	putative transcriptional regulator (TetR family)		-0.91	0.53	0.03
ABAYE1838	hypothetical protein		-1.00	0.50	0.03
ABAYE1843	putative phage/plasmid replication protein		-0.71	0.61	0.01
ABAYE1846	hypothetical protein		0.82	1.77	0.00

ABAYE1851	hypothetical protein		-1.00	0.50	0.02
ABAYE1853	putative phage/plasmid replication protein		-0.76	0.59	0.01
ABAYE1876	hypothetical protein		-3.60	0.08	0.00
ABAYE1886	conserved hypothetical protein%3B putative 5`-nucleotidase domain		-0.90	0.54	0.01
ABAYE1890	hypothetical protein		1.10	2.14	0.02
ABAYE1901	conserved hypothetical protein%3B putative exported protein		0.96	1.95	0.02
ABAYE1907	putative Permease of the major facilitator superfamily		-0.86	0.55	0.02
ABAYE1916	putative acetyl-CoA acetyltransferase (Acetoacetyl-CoA thiolase)		-0.94	0.52	0.03
ABAYE1929	putative 4Fe-4S ferredoxin%3B putative Adenylylsulfate OR formate reductase%2C beta subunit		2.10	4.29	0.03
ABAYE1933	putative acyl carrier protein phosphodiesterase (ACP phosphodiesterase)		1.00	2.00	0.02
ABAYE1935	putative transcriptional regulator (AraC family)		-0.60	0.66	0.02
ABAYE1963	hypothetical protein		-0.92	0.53	0.03
ABAYE1969	hypothetical protein		2.70	6.50	0.01
ABAYE1974	conserved hypothetical protein%3B putative signal peptide		0.79	1.73	0.00
ABAYE2000	conserved hypothetical protein%3B putative membrane protein		1.40	2.64	0.01
ABAYE2001	putative ferric siderophore receptor protein		0.58	1.49	0.04
ABAYE2034	conserved hypothetical protein%3B putative exported protein		2.10	4.29	0.00

ABAYE2051	putative biopolymer transport protein (EXBD-like)		-0.68	0.62	0.03
ABAYE2063	conserved hypothetical protein		1.00	2.00	0.04
ABAYE2072	putative transcriptional regulator		0.51	1.42	0.05
ABAYE2079	putative biopolymer transport protein (EXBD-like)		1.20	2.30	0.01
ABAYE2080	putative biopolymer transport protein (EXBB-like)		1.20	2.30	0.00
ABAYE2109	conserved hypothetical protein		0.75	1.68	0.04
ABAYE2120	conserved hypothetical protein		-1.80	0.29	0.03
ABAYE2132	putative fimbrial protein precursor (Pilin)		1.10	2.14	0.04
ABAYE2133	putative pilin chaperone		1.10	2.14	0.01
ABAYE2137	fragment of putative outer membrane usher protein (part 2)		-1.10	0.47	0.04
ABAYE2140	hypothetical protein%3B putative exported protein		0.69	1.61	0.04
ABAYE2146	conserved hypothetical protein%3B putative membrane protein		-1.10	0.47	0.01
ABAYE2171	putative GTP-binding protein		-0.79	0.58	0.02
ABAYE2174	conserved hypothetical protein%3B putative exported protein		-1.30	0.41	0.02
ABAYE2178	conserved hypothetical protein		-0.61	0.66	0.03
ABAYE2246	putative Short-chain dehydrogenase/reductase%3B putative 3-oxoacyl-[acyl-carrier-protein] reductase		1.10	2.14	0.03
ABAYE2267	hypothetical protein		-2.20	0.22	0.00

ABAYE2269	putative Short-chain dehydrogenase/reductase		-2.30	0.20	0.01
ABAYE2273	hypothetical protein		-3.80	0.07	0.00
ABAYE2274	hypothetical protein		-3.10	0.12	0.00
ABAYE2277	hypothetical protein%3B putative exported protein		-3.10	0.12	0.02
ABAYE2295	conserved hypothetical protein		1.20	2.30	0.03
ABAYE2297	conserved hypothetical protein		1.70	3.25	0.01
ABAYE2298	putative transport protein (MFS superfamily)		0.63	1.55	0.03
ABAYE2323	putative transcriptional regulator (GntR family)		0.93	1.91	0.00
ABAYE2333	putative aldehyde dehydrogenase		0.96	1.95	0.02
ABAYE2334	conserved hypothetical protein		1.20	2.30	0.01
ABAYE2335	conserved hypothetical protein		0.79	1.73	0.04
ABAYE2336	conserved hypothetical protein		1.20	2.30	0.04
ABAYE2347	fragment of putative transcriptional regulator (TetR family)		-0.61	0.66	0.04
ABAYE2350	transposase of ISAba1%2C IS4 family (ORF 1)		-0.49	0.71	0.05
ABAYE2351	transposase of ISAba1%2C IS4 family (ORF 2)		-0.51	0.70	0.04
ABAYE2362	hypothetical protein%3B putative endonuclease		0.80	1.74	0.01
ABAYE2382	putative oxidoreductase		0.64	1.56	0.03
ABAYE2389	conserved hypothetical protein%3B putative exported protein		-0.81	0.57	0.02

ABAYE2407	conserved hypothetical protein		0.82	1.77	0.01
ABAYE2429	conserved hypothetical protein		1.40	2.64	0.01
ABAYE2430	conserved hypothetical protein		1.40	2.64	0.01
ABAYE2436	conserved hypothetical protein		-0.87	0.55	0.03
ABAYE2459	hypothetical protein		-0.91	0.53	0.02
ABAYE2465	putative Permease of the major facilitator superfamily		-1.10	0.47	0.00
ABAYE2475	putative transcriptional regulator (AsnC family)		0.82	1.77	0.04
ABAYE2487	putative outer membrane efflux protein%2C type I secretion protein		0.61	1.53	0.04
ABAYE2497	hypothetical protein		0.75	1.68	0.04
ABAYE2508	conserved hypothetical protein		1.10	2.14	0.03
ABAYE2510	conserved hypothetical protein		-1.10	0.47	0.03
ABAYE2514	conserved hypothetical protein		-0.90	0.54	0.01
ABAYE2521	hypothetical protein		-1.20	0.44	0.02
ABAYE2525	hypothetical protein%3B putative exported protein		-0.93	0.52	0.01
ABAYE2526	putative transcriptional regulator		1.20	2.30	0.02
ABAYE2536	conserved hypothetical protein%3B putative Amino acid efflux-like protein		0.62	1.54	0.04
ABAYE2539	hypothetical protein		0.58	1.49	0.05

ABAYE2546	conserved hypothetical protein		-0.59	0.66	0.03
ABAYE2548	conserved hypothetical protein		-2.10	0.23	0.01
ABAYE2569	conserved hypothetical protein%3B putative exported protein		-1.30	0.41	0.04
ABAYE2611	putative transcriptional regulator (TetR/AcrR family)		1.20	2.30	0.03
ABAYE2617	putative flavin-binding monooxygenase		-1.40	0.38	0.03
ABAYE2619	putative Acetyl esterase		0.82	1.77	0.01
ABAYE2620	putative Cholesterol oxidase		-0.71	0.61	0.03
ABAYE2633	conserved hypothetical protein%3B putative exported protein		1.70	3.25	0.00
ABAYE2634	conserved hypothetical protein		1.70	3.25	0.04
ABAYE2651	hypothetical protein%3B putative membrane protein		-1.30	0.41	0.04
ABAYE2652	hypothetical protein%3B putative Rhs family protein		-1.10	0.47	0.01
ABAYE2654	hypothetical protein%3B putative exported protein		1.00	2.00	0.02
ABAYE2655	conserved hypothetical protein%3B putative exported protein		-0.61	0.66	0.04
ABAYE2665	conserved hypothetical protein		1.80	3.48	0.01
ABAYE2669	conserved hypothetical protein		0.84	1.79	0.00
ABAYE2671	conserved hypothetical protein%3B putative haemocin immunity protein		0.64	1.56	0.05
ABAYE2689	conserved hypothetical protein		-1.80	0.29	0.02
ABAYE2695	hypothetical protein%3B putative acetyltransferase%2C GNAT family		-0.84	0.56	0.02

ABAYE2698	putative bacteriophage protein		-1.30	0.41	0.03
ABAYE2701	conserved hypothetical protein from bacteriophage		-0.73	0.60	0.04
ABAYE2703	hypothetical protein		-1.90	0.27	0.03
ABAYE2713	conserved hypothetical protein		-2.60	0.16	0.02
ABAYE2727	conserved hypothetical protein		-1.20	0.44	0.01
ABAYE2732	conserved hypothetical protein		-0.97	0.51	0.02
ABAYE2738	hypothetical protein		-1.20	0.44	0.04
ABAYE2741	conserved hypothetical protein		-1.20	0.44	0.02
ABAYE2757	conserved hypothetical protein%3B putative signal peptide		-1.30	0.41	0.03
ABAYE2782	putative lipoprotein		-1.50	0.35	0.00
ABAYE2792	conserved hypothetical protein		1.00	2.00	0.01
ABAYE2800	putative hydrolase (EstB)		0.56	1.47	0.03
ABAYE2813	putative membrane-bound protein in GNT I transport system (GntY)		1.10	2.14	0.00
ABAYE2817	conserved hypothetical protein%3B putative lipoprotein precursor		-2.00	0.25	0.00
ABAYE2821	conserved hypothetical protein		1.30	2.46	0.00
ABAYE2823	putative transketolase C-terminal section		1.10	2.14	0.01
ABAYE2824	putative transketolase N-terminal section		1.30	2.46	0.05
ABAYE2826	putative 3-oxoacyl-[acyl-carrier-protein] reductase (3-ketoacyl-acyl carrier protein		0.78	1.72	0.03

	reductase)				
ABAYE2836	putative acetolactate synthase (IlvB-like)		0.64	1.56	0.03
ABAYE2837	putative aldehyde dehydrogenase		1.20	2.30	0.02
ABAYE2838	putative L-aspartate dehydrogenase (NadX)		1.30	2.46	0.03
ABAYE2839	putative short-chain dehydrogenase		1.40	2.64	0.00
ABAYE2842	putative permease (MFS superfamily)		0.47	1.39	0.04
ABAYE2843	putative ferredoxin reductase component (dioxygenase)		1.30	2.46	0.04
ABAYE2847	conserved hypothetical protein		1.50	2.83	0.04
ABAYE2850	conserved hypothetical protein		-0.98	0.51	0.02
ABAYE2871	putative homocysteine S-methyltransferase family protein		1.10	2.14	0.04
ABAYE2881	hypothetical protein		-0.81	0.57	0.02
ABAYE2891	hypothetical protein%3B putative membrane protein from bacteriophage		-1.10	0.47	0.01
ABAYE2895	hypothetical protein from bacteriophage		-1.70	0.31	0.04
ABAYE2896	hypothetical protein from bacteriophage		-2.90	0.13	0.00
ABAYE2897	hypothetical protein from bacteriophage		-2.60	0.16	0.03
ABAYE2903	hypothetical protein from bacteriophage		-0.74	0.60	0.04
ABAYE2925	putative glutamine amidotransferase		0.59	1.51	0.03
ABAYE2942	putative arginine-tRNA-protein transferase		0.84	1.79	0.05

ABAYE2959	fragment of putative dioxygenase alpha subunit (part 2)		-0.93	0.52	0.03
ABAYE2962	putative Lipase		1.30	2.46	0.01
ABAYE2985	conserved hypothetical protein%3B putative exported protein		-0.56	0.68	0.03
ABAYE2988	putative hydrolase		0.56	1.47	0.05
ABAYE2990	conserved hypothetical protein		-1.20	0.44	0.00
ABAYE3005	putative carboxylesterase%2C biotin biosynthesis (BioH)		-0.58	0.67	0.04
ABAYE3008	putative transport protein (permease)		-1.40	0.38	0.01
ABAYE3013	putative transcriptional regulator (GntR-family)		1.10	2.14	0.00
ABAYE3030	conserved hypothetical protein		-1.60	0.33	0.00
ABAYE3032	conserved hypothetical protein%3B putative exported protein		-0.93	0.52	0.05
ABAYE3036	putative multidrug resistance efflux pump		0.97	1.96	0.04
ABAYE3040	conserved hypothetical protein%3B putative membrane protein		-1.80	0.29	0.01
ABAYE3041	conserved hypothetical protein		-2.50	0.18	0.00
ABAYE3068	putative outermembrane protein exposed to the bacterial surface		-0.74	0.60	0.02
ABAYE3069	conserved hypothetical protein		0.53	1.44	0.03
ABAYE3072	hypothetical protein		0.76	1.69	0.04
ABAYE3074	hypothetical protein%3B putative membrane protein		2.20	4.59	0.00
ABAYE3080	conserved hypothetical protein		0.71	1.64	0.05

ABAYE3082	putative transcriptional regulator (LysR family)		1.30	2.46	0.05
ABAYE3120	conserved hypothetical protein		1.50	2.83	0.03
ABAYE3122	putative MutT/nudix family protein		0.89	1.85	0.04
ABAYE3125	putative pilus assembly protein (FilD)		0.70	1.62	0.03
ABAYE3127	putative pilus assembly protein (FilB)		0.93	1.91	0.05
ABAYE3135	putative sigma(54) modulation protein RpoX		-2.00	0.25	0.00
ABAYE3165	putative transporter		-0.78	0.58	0.02
ABAYE3167	putative glutathione S-transferase		0.92	1.89	0.02
ABAYE3212	hypothetical protein%3B putative exported protein		-2.00	0.25	0.02
ABAYE3235	putative transcriptional regulator (TetR family)		-1.00	0.50	0.03
ABAYE3290	putative ferric siderophore receptor protein		0.63	1.55	0.02
ABAYE3309	conserved hypothetical protein		-0.71	0.61	0.03
ABAYE3310	conserved hypothetical protein		0.95	1.93	0.04
ABAYE3333	conserved hypothetical protein%3B putative membrane protein		-1.20	0.44	0.02
ABAYE3339	putative Zn-dependent oxidoreductase		0.71	1.64	0.03
ABAYE3342	putative transporter protein		0.47	1.39	0.04
ABAYE3350	conserved hypothetical protein		1.90	3.73	0.00
ABAYE3370	conserved hypothetical protein%3B putative membrane protein		0.90	1.87	0.04

ABAYE3386	putative type III effector HopPmaJ		1.20	2.30	0.00
ABAYE3425	conserved hypothetical protein		-0.82	0.57	0.03
ABAYE3457	conserved hypothetical protein		0.85	1.80	0.01
ABAYE3473	conserved hypothetical protein%3B putative signal peptide		-0.91	0.53	0.05
ABAYE3475	conserved hypothetical protein%3B putative exported protein		-0.83	0.56	0.02
ABAYE3485	conserved hypothetical protein		0.91	1.88	0.01
ABAYE3487	conserved hypothetical protein%3B putative exported protein		-0.89	0.54	0.04
ABAYE3503	putative DNA binding protein		-1.10	0.47	0.00
ABAYE3522	putative glutamyl t-RNA synthetase		-0.62	0.65	0.03
ABAYE3525	conserved hypothetical protein%3B putative exported protein		-0.91	0.53	0.01
ABAYE3561	fragment of conjugal transfer protein trbI		-0.71	0.61	0.05
ABAYE3599	putative Relaxase/helicase		-1.50	0.35	0.01
ABAYE3602	fragment of conserved hypothetical protein (part 2)		0.85	1.80	0.02
ABAYE3615	putative transposase		0.53	1.44	0.03
ABAYE3667	conserved hypothetical protein		0.93	1.91	0.04
ABAYE3674	putative outer membrane protein		1.40	2.64	0.01
ABAYE3676	conserved hypothetical protein%3B putative membrane protein		0.79	1.73	0.01
ABAYE3695	conserved hypothetical protein		-0.72	0.61	0.02

ABAYE3702	fragment of conserved hypothetical protein (partial)		-1.60	0.33	0.00
ABAYE3706	conserved hypothetical protein%3B putative exported protein		2.40	5.28	0.00
ABAYE3707	putative Rossmann-fold nucleotide-binding protein involved in DNA uptake (Smf)		1.70	3.25	0.01
ABAYE3708	putative ribosome maturation factor (dsRNA-binding protein) (RimN)		0.65	1.57	0.02
ABAYE3711	putative transporter (MFS superfamily)		0.62	1.54	0.03
ABAYE3736	conserved hypothetical protein		0.93	1.91	0.03
ABAYE3744	putative membrane protein		0.92	1.89	0.01
ABAYE3763	putative enoyl-CoA hydratase/isomerase family protein		1.10	2.14	0.03
ABAYE3765	putative acyl-CoA dehydrogenase		1.70	3.25	0.04
ABAYE3776	conserved hypothetical protein%3B putative membrane protein		0.65	1.57	0.03
ABAYE3777	putative ferric siderophore receptor protein		0.62	1.54	0.03
ABAYE3782	hypothetical protein		-1.10	0.47	0.00
ABAYE3794	putative transcriptional regulator (GntR family)		0.94	1.92	0.02
ABAYE3807	putative acetyltransferase (WeeI)		0.82	1.77	0.04
ABAYE3810	conserved hypothetical protein%3B putative polysaccharide polymerase		0.94	1.92	0.01
ABAYE3824	fragment of conserved hypothetical protein (partial)		-0.66	0.63	0.03
ABAYE3856	conserved hypothetical protein		-1.20	0.44	0.01

ABAYE3860	Conserved hypothetical protein%3B putative signal peptide		-1.50	0.35	0.00
ABAYE3866	hypothetical protein%3B putative peptidase M10A and M12B%2C matrixin and adamalysin%3B putative signal peptide		-0.84	0.56	0.03
ABAYE3868	conserved hypothetical protein		-1.60	0.33	0.01
ABAYE3874	conserved hypothetical protein%3B putative membrane protein		0.65	1.57	0.04
ABAYE3888	conserved hypothetical protein%3B putative membrane protein		-1.60	0.33	0.03
ABAYE3889	putative thiol:disulfide interchange protein		-1.00	0.50	0.00
ABAYEmisc_RNA_9			0.74	1.67	0.01
ABAYEtRNA17	ABAYEtRNA17		0.89	1.85	0.02
ABAYEtRNA20	ABAYEtRNA20		-2.10	0.23	0.00
ABAYEtRNA21	ABAYEtRNA21		1.30	2.46	0.02
ABAYEtRNA23	ABAYEtRNA23		-2.10	0.23	0.00
ABAYEtRNA25	ABAYEtRNA25		-2.20	0.22	0.00
ABAYEtRNA41	ABAYEtRNA41		1.60	3.03	0.00
ABAYEtRNA43	ABAYEtRNA43		0.96	1.95	0.01
ABAYEtRNA45	ABAYEtRNA45		1.50	2.83	0.01
ABAYEtRNA46	ABAYEtRNA46		-1.20	0.44	0.01
ABAYEtRNA51	ABAYEtRNA51		0.90	1.87	0.01

ABAYEtRNA54	ABAYEtRNA54		0.92	1.89	0.01
ABAYEtRNA57	ABAYEtRNA57		-2.40	0.19	0.00
ABAYEtRNA6	ABAYEtRNA6		-1.30	0.41	0.02
ABAYEtRNA63	ABAYEtRNA63		-3.00	0.13	0.00
ABAYEtRNA7	ABAYEtRNA7		-1.40	0.38	0.01
ABAYEtRNA8	ABAYEtRNA8		-1.30	0.41	0.01
