# Supplementary material for: The Acinetobacter baumannii Two-Component System AdeRS Regulates Genes Required for Multidrug Efflux, Biofilm Formation, and Virulence in a Strain-Specific Manner
Source: mBio. 2016 Apr 19;7(2):e00430-16. doi: 10.1128/mBio.00430-16 (PMC4850262; doi:10.1128/mBio.00430-16)
Supplement: Table S2 — Gene expression data from the complete transcriptome analysis of A. baumannii AYE by RNA-Seq, showing differentially expressed genes (P < 0.05) in AYEΔadeB compared with AYE. [file mbo002162774st2.pdf]

Table S2 Gene expression data from the complete transcriptome analysis of *A. baumannii* AYE by RNA-Seq, showing differentially expressed genes ($P < 0.05$) in AYEΔ*adeB* compared with AYE.

ABAYE Gene ID	Annotation	Gene Name	log2 Fold Change	Fold Change	P.Value
ABAYE_16s_1	ABAYE_16s_1		1.10	2.14	0.00
ABAYE_16s_2	ABAYE_16s_2		1.10	2.14	0.00
ABAYE_16s_3	ABAYE_16s_3		1.20	2.30	0.00
ABAYE_16s_4	ABAYE_16s_4		1.10	2.14	0.01
ABAYE_16s_5	ABAYE_16s_5		1.10	2.14	0.01
ABAYE_16s_6	ABAYE_16s_6		1.20	2.30	0.00
ABAYE_23s_1	ABAYE_23s_1		0.73	1.66	0.00
ABAYE_23s_2	ABAYE_23s_2		0.72	1.65	0.00
ABAYE_23s_3	ABAYE_23s_3		0.64	1.56	0.01
ABAYE_23s_4	ABAYE_23s_4		0.81	1.75	0.00
ABAYE_23s_5	ABAYE_23s_5		0.63	1.55	0.01
ABAYE_23s_6	ABAYE_23s_6		0.81	1.75	0.00
ABAYE0009	putative RND type efflux pump involved in aminoglycoside resistance (AdeT)	adeT	0.48	1.39	0.04

ABAYE0016	putative glutathione S-transferase		-0.62	0.65	0.02
ABAYE0038	putative transport protein (MFS superfamily)		0.94	1.92	0.00
ABAYE0039	aldehyde reductase	alrA	0.98	1.97	0.00
ABAYE0041	conserved hypothetical protein		-0.81	0.57	0.00
ABAYE0050	conserved hypothetical protein%3B putative exported protein		1.30	2.46	0.00
ABAYE0052	putative PQQ-dependent aldose sugar dehydrogenase precursor		0.41	1.33	0.04
ABAYE0064	4-hydroxyphenylpyruvate dioxygenase (4HPPD)(HPPDase)	hpd	-0.34	0.79	0.05
ABAYE0066	putative homogentisate 1%2C2-dioxygenase		-0.51	0.70	0.01
ABAYE0067	maleylacetoacetate isomerase (MAAI)	hmgC	-0.45	0.73	0.04
ABAYE0068	fumarylacetoacetase (Fumarylacetoacetate hydrolase)	hmgB	-0.57	0.67	0.03
ABAYE0069	aromatic amino acid transporter (APC family)	aroP	-0.56	0.68	0.02
ABAYE0071	putative regulatory protein (nitrile hydratase activator like)		0.73	1.66	0.01
ABAYE0080	conserved hypothetical protein%3B putative signal peptide		-0.59	0.66	0.05
ABAYE0081	putative hydrolase		-0.57	0.67	0.04
ABAYE0082	putative glutamate racemase		-0.82	0.57	0.01
ABAYE0085	conserved hypothetical protein%3B putative two-component regulatory system		-0.62	0.65	0.00
ABAYE0086	conserved hypothetical protein		-0.73	0.60	0.00
ABAYE0088	hypothetical protein		-0.69	0.62	0.03

ABAYE0089	glucosamine--fructose-6-phosphate aminotransferase	glmS	0.36	1.28	0.01
ABAYE0090	bifunctional protein [Includes: UDP-N-acetylglucosamine pyrophosphorylase (N-acetylglucosamine-1-phosphate uridyltransferase)%3B Glucosamine-1-phosphate N-acetyltransferase]	glmU	0.45	1.37	0.03
ABAYE0091	phosphatidylglycerophosphatase A	pgpA	0.53	1.44	0.01
ABAYE0105	conserved hypothetical protein%3B putative secretory lipase precursor		-0.56	0.68	0.04
ABAYE0111	putative aminotransferase		0.40	1.32	0.01
ABAYE0112	conserved hypothetical protein		0.45	1.37	0.00
ABAYE0113	conserved hypothetical protein%3B putative exported protein		0.48	1.39	0.00
ABAYE0115	conserved hypothetical protein		0.40	1.32	0.03
ABAYE0118	conserved hypothetical protein		-0.42	0.75	0.05
ABAYE0119	hypothetical protein		-0.78	0.58	0.00
ABAYE0121	hypothetical protein%3B putative signal peptide		-0.59	0.66	0.01
ABAYE0123	hypothetical protein%3B putative signal peptide		-0.85	0.55	0.02
ABAYE0127	ureidoglycolate amidohydrolase(decarboxylating)	allA	-0.37	0.77	0.02
ABAYE0129	putative flavoprotein monooxygenase acting on aromatic compound		0.47	1.39	0.01
ABAYE0146	conserved hypothetical protein		-0.50	0.71	0.00
ABAYE0148	UDP-N-acetylglucosamine:N-acetylmuramyl-(pentapeptide) pyrophosphoryl-undecaprenol N-acetylglucosamine transferase	murG	0.33	1.26	0.02

ABAYE0149	UDP-N-acetylmuramate--alanine ligase	murC	0.34	1.27	0.01
ABAYE0158	dihydrolipoamide S-acetyltransferase%2C E2 component of the pyruvate dehydrogenase complex	aceF	-0.24	0.85	0.05
ABAYE0164	conserved hypothetical protein%3B putative enzyme		-2.80	0.14	0.00
ABAYE0165	putative ferredoxin		-1.60	0.33	0.00
ABAYE0168	pyridoxamine 5-phosphate oxidase (acts also on pyridoxine phosphate and pyridoxine)	pdxH	0.35	1.27	0.01
ABAYE0170	conserved hypothetical protein%3B putative exported protein		1.30	2.46	0.00
ABAYE0173	putative transcriptional regulator (ArsR family)		0.34	1.27	0.04
ABAYE0174	xenobiotic reductase	xenB	0.47	1.39	0.00
ABAYE0175	3-deoxy-D-manno-2-octulosonate transferase	kdtA	0.36	1.28	0.01
ABAYE0176	conserved hypothetical protein		0.49	1.40	0.00
ABAYE0182	putative sulfonate monooxygenase (MsuD)		0.65	1.57	0.01
ABAYE0190	putative high affinity choline transport protein (Bet-like)		0.61	1.53	0.00
ABAYE0191	conserved hypothetical protein%3B putative exported protein		-0.46	0.73	0.02
ABAYE0192	transposase of ISAba1%2C IS4 family (ORF 2)		-0.63	0.65	0.03
ABAYE0193	transposase of ISAba1%2C IS4 family (ORF 1)		-0.60	0.66	0.00
ABAYE0199	membrane protein required for efficient alginate biosynthesis	glpM	0.64	1.56	0.01
ABAYE0202	putative transport protein (MFS superfamily)		-0.35	0.78	0.01

ABAYE0203	ssDNA-binding protein controls activity of RecBCD nuclease	ssb	-0.34	0.79	0.02
ABAYE0212	putative hydrolase%2C isochorismatase family		0.73	1.66	0.00
ABAYE0216	conserved hypothetical protein%3B putative glutathione-dependent formaldehyde-activating		0.31	1.24	0.03
ABAYE0227	conserved hypothetical protein		-0.80	0.57	0.01
ABAYE0228	hypothetical protein		-1.50	0.35	0.00
ABAYE0235	conserved hypothetical protein%3B putative signal peptide		3.20	9.19	0.00
ABAYE0252	conserved hypothetical protein%3B putative membrane protein		-1.20	0.44	0.00
ABAYE0262	putative sulfate permease		4.70	25.99	0.00
ABAYE0264	homocysteine synthase	metY	-0.34	0.79	0.02
ABAYE0266	conserved hypothetical protein%3B putative signal peptide		-2.00	0.25	0.00
ABAYE0275	conserved hypothetical protein		-0.74	0.60	0.00
ABAYE0278	conserved hypothetical protein%3B putative exported protein		-0.61	0.66	0.02
ABAYE0279	sulfate transport protein (ABC superfamily%2C peri_bind)	sbp	-0.69	0.62	0.00
ABAYE0290	putative membrane protein (ComM)		-2.60	0.16	0.00
ABAYE0291	putative membrane protein (ComN)		-3.30	0.10	0.00
ABAYE0292	putative membrane protein (ComO)		-3.00	0.13	0.00
ABAYE0293	putative lipoprotein (ComL)		-3.10	0.12	0.00

ABAYE0294	putative outer membrane protein (ComQ)		-2.80	0.14	0.00
ABAYE0295	shikimate-kinase	aroK	-0.48	0.72	0.00
ABAYE0300	conserved hypothetical protein		0.45	1.37	0.02
ABAYE0302	conserved hypothetical protein%3B putative membrane protein		0.64	1.56	0.01
ABAYE0303	conserved hypothetical protein		0.77	1.71	0.00
ABAYE0304	putative fimbrial protein precursor (Pilin)		-5.20	0.03	0.00
ABAYE0308	putative regulatory or redox component complexing with Bfr%2C in iron storage and mobility (Bfd)		0.67	1.59	0.01
ABAYE0314	putative Fimbrial protein precursor%3B putative type IV pilin protein		-1.70	0.31	0.00
ABAYE0315	putative type IV fimbrial biogenesis protein		-2.00	0.25	0.00
ABAYE0316	pilin like competence factor	comB	-2.20	0.22	0.00
ABAYE0317	putative type IV fimbrial biogenesis protein		-2.30	0.20	0.00
ABAYE0318	putative competence factor involved in DNA binding and uptake (ComC)		-1.70	0.31	0.00
ABAYE0319	Pilin like competence factor	comE	-1.50	0.35	0.00
ABAYE0320	pilin like competence factor	comF	-1.40	0.38	0.00
ABAYE0329	conserved hypothetical protein		0.91	1.88	0.00
ABAYE0330	conserved hypothetical protein%3B putative membrane protein		0.29	1.22	0.04
ABAYE0334	conserved hypothetical protein%3B putative exported protein		1.00	2.00	0.00

ABAYE0335	conserved hypothetical protein		0.72	1.65	0.00
ABAYE0336	modulator of drug activity%2C similar to electron transfer flavoprotein-NAD/FAD/quinone oxidoreductase	mdaB	0.56	1.47	0.00
ABAYE0337	conserved hypothetical protein%3B putative signal peptide		0.60	1.52	0.00
ABAYE0340	superoxide dismutase precursor (Cu-Zn)	sodC	0.67	1.59	0.00
ABAYE0345	conserved hypothetical protein%3B putative exported protein		0.61	1.53	0.03
ABAYE0346	conserved hypothetical protein%3B putative exported protein		0.28	1.21	0.03
ABAYE0347	conserved hypothetical protein		0.41	1.33	0.01
ABAYE0349	putative APC family%2C S-methylmethionine transporter (MmuP)		0.47	1.39	0.03
ABAYE0352	succinylornithine transaminase (also has acetylornitine transaminase activity%2C PLP-dependent) (carbon starvation protein C)	astC	0.32	1.25	0.02
ABAYE0353	arginine succinyltransferase	astA	0.52	1.43	0.02
ABAYE0357	conserved hypothetical protein%3B putative signal peptide		1.30	2.46	0.00
ABAYE0359	putative alkaline protease		0.63	1.55	0.02
ABAYE0360	conserved hypothetical protein%3B putative signal peptide		0.77	1.71	0.00
ABAYE0361	conserved hypothetical protein%3B putative signal peptide		0.83	1.78	0.00
ABAYE0374	conserved hypothetical protein%3B putative signal peptide		0.50	1.41	0.00
ABAYE0376	conserved hypothetical protein%3B putative signal peptide		-0.71	0.61	0.00
ABAYE0393	putative DNA transformation protein (ComF)		0.31	1.24	0.02

ABAYE0395	conserved hypothetical protein		0.46	1.38	0.01
ABAYE0399	putative Proton/sodium-glutamate symport protein		0.26	1.20	0.03
ABAYE0400	conserved hypothetical protein		0.35	1.27	0.02
ABAYE0402	conserved hypothetical protein		0.24	1.18	0.04
ABAYE0410	50S ribosomal protein L23	rplW	-0.31	0.81	0.02
ABAYE0411	50S ribosomal protein L2	rplB	-0.49	0.71	0.01
ABAYE0415	50S ribosomal protein L16	rplP	-0.23	0.85	0.05
ABAYE0416	50S ribosomal protein L29	rpmC	-0.27	0.83	0.03
ABAYE0433	RNA polymerase%2C alpha subunit	rpoA	-0.50	0.71	0.01
ABAYE0436	acyl coenzyme A dehydrogenase	fadE	0.51	1.42	0.02
ABAYE0439	conserved hypothetical protein%3B putative exported protein		0.41	1.33	0.02
ABAYE0441	putative integral membrane protein%2C transporter		0.63	1.55	0.00
ABAYE0447	conserved hypothetical protein%3Bputative HAD-superfamily subfamily IB%2C PSPase-like		-0.53	0.69	0.01
ABAYE0459	conserved hypothetical protein%3B putative tonB-domain		-0.68	0.62	0.00
ABAYE0464	conserved hypothetical protein%3B putative exported protein		0.60	1.52	0.00
ABAYE0474	conserved hypothetical protein		0.45	1.37	0.04
ABAYE0479	putative dehydrogenase		0.54	1.45	0.02

ABAYE0481	putative acyl-CoA dehydrogenase		0.47	1.39	0.00
ABAYE0487	30S ribosomal protein S9	rpsI	-0.29	0.82	0.03
ABAYE0492	conserved hypothetical protein%3B putative signal peptide		-0.77	0.59	0.00
ABAYE0505	putative pyridine nucleotide-disulfide oxidoreductase%2C class I		-0.84	0.56	0.00
ABAYE0506	conserved hypothetical protein		-2.80	0.14	0.00
ABAYE0510	transposase of ISAba1%2C IS4 family (ORF 1)		-0.59	0.66	0.03
ABAYE0522	conserved hypothetical protein%3B putative membrane protein		-0.67	0.63	0.00
ABAYE0523	DNA repair protein%2C associated with replication forks	radC	-0.78	0.58	0.00
ABAYE0524	bifunctional protein [Includes: 4-phosphopantothenoylcysteine decarboxylase%3B phosphopantothenoylcysteine synthetase%2C FMN-binding]	dfp	-0.25	0.84	0.04
ABAYE0529	conserved hypothetical protein		-1.90	0.27	0.00
ABAYE0530	conserved hypothetical protein%3B putative membrane protein		-1.20	0.44	0.00
ABAYE0537	hypothetical protein		0.28	1.21	0.03
ABAYE0539	hypothetical protein		0.65	1.57	0.02
ABAYE0540	hypothetical protein		0.53	1.44	0.02
ABAYE0548	hypothetical protein%3B putative NAD-dependent DNA ligase		-0.84	0.56	0.00
ABAYE0549	conserved hypothetical protein%3B putative exported protein		-0.52	0.70	0.00
ABAYE0552	phage-related late control gene protein (GPD-like)		0.45	1.37	0.03

ABAYE0559	putative phage tail protein		-0.49	0.71	0.02
ABAYE0570	putative Phage small terminase subunit precursor		-0.62	0.65	0.05
ABAYE0571	phage-related capsid protein precursor (GPN-like)		-0.57	0.67	0.02
ABAYE0572	phage-related capsid scaffolding protein (GPO-like)		-1.20	0.44	0.00
ABAYE0579	conserved hypothetical protein		-0.30	0.81	0.02
ABAYE0585	conserved hypothetical protein		-0.95	0.52	0.00
ABAYE0589	conserved hypothetical protein%3B putative membrane protein		1.00	2.00	0.00
ABAYE0603	conserved hypothetical protein%3B putative signal peptide		0.69	1.61	0.00
ABAYE0604	putative glycerophosphodiester phosphodiesterase		0.35	1.27	0.04
ABAYE0613	tryptophan synthase alpha chain	trpA	0.48	1.39	0.01
ABAYE0619	putative antioxidant protein		1.00	2.00	0.00
ABAYE0621	conserved hypothetical protein%3B putative exported protein		0.41	1.33	0.01
ABAYE0631	conserved hypothetical protein		0.45	1.37	0.02
ABAYE0632	putative glucose-sensitive porin (OprB-like)		0.50	1.41	0.00
ABAYE0633	glucose dehydrogenase [pyrroloquinoline-quinone] precursor (Quinoprotein glucose DH)	gcd	0.61	1.53	0.00
ABAYE0637	conserved hypothetical protein		0.63	1.55	0.01
ABAYE0639	putative type IV fimbrial biogenesis protein		-0.52	0.70	0.02

ABAYE0640	putative Outer membrane protein precursor (OmpA-like)		-0.32	0.80	0.01
ABAYE0647	mechanosensitive channel	mscL	0.37	1.29	0.02
ABAYE0659	conserved hypothetical protein		0.52	1.43	0.02
ABAYE0662	conserved hypothetical protein		0.74	1.67	0.00
ABAYE0663	prolyl-tRNA synthetase	proS	-0.25	0.84	0.03
ABAYE0666	conserved hypothetical protein%3B putative exported protein		-1.70	0.31	0.00
ABAYE0667	twitching motility protein	pilG	-2.80	0.14	0.00
ABAYE0668	twitching motility protein	pilH	-2.70	0.15	0.00
ABAYE0669	twitching motility protein	pilI	-3.50	0.09	0.00
ABAYE0670	type IV pilus biogenesis protein	pilJ	-4.00	0.06	0.00
ABAYE0671	putative sensor histidine kinase/response regulator%3B putative protein of chemotactic signal transduction system (ChA-like)		-3.60	0.08	0.00
ABAYE0672	conserved hypothetical protein		-2.70	0.15	0.00
ABAYE0674	putative Oxygen-independent coproporphyrinogen III oxidase		-3.30	0.10	0.00
ABAYE0675	conserved hypothetical protein%3B putative enzyme		-2.40	0.19	0.00
ABAYE0676	succinyl-diaminopimelate desuccinylase	dapE	0.46	1.38	0.02
ABAYE0677	bacteriolytic lipoprotein entericidin B.	ecnB	2.30	4.92	0.00
ABAYE0682	putative bifunctional protein [Includes: sulfite reductase [NADPH] flavoprotein alpha-component%3B iron-uptake factor]		-1.10	0.47	0.00

ABAYE0690	conserved hypothetical protein%3B putative exported protein		0.98	1.97	0.00
ABAYE0693	conserved hypothetical protein%3B putative exported protein		-0.37	0.77	0.03
ABAYE0694	conserved hypothetical protein%3B putative exported protein		-0.41	0.75	0.04
ABAYE0695	conserved hypothetical protein%3B putative exported protein		0.66	1.58	0.00
ABAYE0703	rod shape-determining protein	mreD	0.43	1.35	0.04
ABAYE0704	putative Maf-like protein		0.38	1.30	0.05
ABAYE0707	conserved hypothetical protein		-0.46	0.73	0.04
ABAYE0708	bifunctional protein [Includes: wax ester synthase / acyl-CoA:diacylglycerol acyltransferase]	wax-dgaT	-0.57	0.67	0.04
ABAYE0730	conserved hypothetical protein%3B putative protein (DcaP-like)		-0.79	0.58	0.00
ABAYE0731	lipid A phosphoethanolamine transferase%2C associated with polymyxin resistance	eptA	-1.40	0.38	0.00
ABAYE0732	transcriptional regulator	qseB	-0.68	0.62	0.00
ABAYE0733	two-component sensor kinase transcription regulator protein	qseC	-3.30	0.10	0.00
ABAYE0735	putative ammonium transporter		-0.92	0.53	0.01
ABAYE0738	conserved hypothetical protein		0.46	1.38	0.02
ABAYE0743	conserved hypothetical protein%3B putative enzyme		0.28	1.21	0.02
ABAYE0748	putative membrane fusion protein (AdeA-like)		0.32	1.25	0.01
ABAYE0749	putative phosphatidylglycerophosphatase B (PgpB)		0.41	1.33	0.02

ABAYE0751	octaprenyl-diphosphate synthase (Octaprenyl pyrophosphate synthetase) (OPP synthetase)	ispB	-0.30	0.81	0.01
ABAYE0754	50S ribosomal protein L27	rpmA	-0.32	0.80	0.01
ABAYE0758	multifunctional protein [Includes: Uroporphyrin-III C-methyltransferase (Urogen III methylase) (SUMT) (Uroporphyrinogen III methylase) (UROM)%3B Precorrin-2 oxidase %3B Ferrochelatase] (Siroheme synthase)	cysG	0.30	1.23	0.02
ABAYE0786	hypothetical protein		-0.57	0.67	0.00
ABAYE0792	conserved hypothetical protein%3B putative biofilm-associated protein with RTX toxins and related Ca2+-binding domain		1.60	3.03	0.00
ABAYE0793	conserved hypothetical protein		-0.51	0.70	0.01
ABAYE0794	hypothetical protein%3B putative bifunctional protein: [Includes: enzyme of poly-gamma-glutamate biosynthesis%3B D-alanyl-D-alanine carboxypeptidase]		-0.44	0.74	0.01
ABAYE0795	hypothetical protein%3B putative metalloprotease		-0.51	0.70	0.02
ABAYE0799	transcription elongation factor%2C cleaves 3 nucleotide of paused mRNA	greA	-0.41	0.75	0.02
ABAYE0802	conserved hypothetical protein		-0.50	0.71	0.00
ABAYE0803	conserved hypothetical protein		0.37	1.29	0.03
ABAYE0804	putative RNA-binding protein		0.33	1.26	0.04
ABAYE0821	hypothetical protein%3B putative surface adhesion protein with RTX toxins and related Ca2+-binding proteins domains		-1.90	0.27	0.00
ABAYE0824	diacylglycerol kinase	dgkA	-1.50	0.35	0.00

ABAYE0825	conserved hypothetical protein%3B putative Metallo-beta-lactamase		-0.70	0.62	0.00
ABAYE0826	putative glycerophosphoryl diester phosphodiesterase%2C periplasmic precursor protein (GLPQ)		0.40	1.32	0.03
ABAYE0831	putative transglycosylase		-0.62	0.65	0.00
ABAYE0832	conserved hypothetical protein		0.60	1.52	0.04
ABAYE0833	conserved hypothetical protein		0.27	1.21	0.04
ABAYE0838	putative peptide synthetase		-1.20	0.44	0.00
ABAYE0839	hypothetical protein%3B putative membrane protein		-0.91	0.53	0.00
ABAYE0840	hypothetical protein%3B putative membrane protein		1.50	2.83	0.00
ABAYE0846	putative nitroreductase		0.54	1.45	0.03
ABAYE0848	putative HTH-type transcriptional protein (TetR family)		0.54	1.45	0.01
ABAYE0874	conserved hypothetical protein		-0.24	0.85	0.05
ABAYE0879	conserved hypothetical protein		-0.82	0.57	0.02
ABAYE0893	putative outer membrane protein		-0.51	0.70	0.02
ABAYE0901	peptidoglycan-associated lipoprotein precursor	pal	0.61	1.53	0.00
ABAYE0902	tolerance to colicins E2%2C E%2C A%2C and K%2C required for OM integrity	tolB	0.55	1.46	0.00
ABAYE0904	tolerance to group A colicins%2C single-stranded filamentous DNA phage%2C required for OM integrity	tolA	0.47	1.39	0.04

ABAYE0905	tolerance to group A colicins%2C single-stranded DNA filamentous phage%2C required for OM integrity	tolR	0.71	1.64	0.00
ABAYE0906	tolerance to group A colicins%2C single-stranded DNA filamentous phage%2C required for OM integrity	tolQ	0.52	1.43	0.02
ABAYE0916	putative nicotinamide/nicotinate-nucleotide adenylyltransferase		0.47	1.39	0.04
ABAYE0917	conserved hypothetical protein%3B putative signal peptide		0.46	1.38	0.05
ABAYE0920	conserved hypothetical protein%3B putative exported protein		0.55	1.46	0.00
ABAYE0924	putative porin protein associated with imipenem resistance		2.10	4.29	0.00
ABAYE0926	sulfate permease A protein%2C chromate resistance (ABC superfamily%2C atp_bind)	cysA	0.30	1.23	0.04
ABAYE0927	sulfate transport protein (ABC superfamily%2C membrane)	cysW	0.55	1.46	0.01
ABAYE0928	sulfate transport protein (ABC superfamily%2C membrane)	cysT	0.48	1.39	0.04
ABAYE0936	putative serine protease		0.54	1.45	0.01
ABAYE0940	conserved hypothetical protein%3B putative exported protein		-0.44	0.74	0.00
ABAYE0947	putative L-allo-threonine dehydrogenase%2C NADP+-dependent (other substrates include L-serine%2C D-serine%2C D-threonine and 3-hydroxyisobutyrate)		-0.41	0.75	0.01
ABAYE0952	conserved hypothetical protein		0.33	1.26	0.02
ABAYE0966	conserved hypothetical protein%3B putative exported protein		-0.57	0.67	0.03
ABAYE0975	deoxyribodipyrimidine photolyase (photoreactivation)%2C FAD-binding	phrB	-1.00	0.50	0.00
ABAYE0977	NADH dehydrogenase	ndh	0.41	1.33	0.04

ABAYE0979	conserved hypothetical protein		-0.26	0.84	0.04
ABAYE0986	putative transcriptional regulator (LysR family)		-0.65	0.64	0.00
ABAYE0990	putative protease		-0.41	0.75	0.01
ABAYE0992	conserved hypothetical protein		-0.39	0.76	0.03
ABAYE0993	conserved hypothetical protein		-0.46	0.73	0.03
ABAYE0994	conserved hypothetical protein		0.50	1.41	0.05
ABAYE0995	conserved hypothetical protein		-0.88	0.54	0.00
ABAYE0996	conserved hypothetical protein%3B putative enzyme		-0.92	0.53	0.00
ABAYE0997	conserved hypothetical protein		-0.66	0.63	0.01
ABAYE0998	conserved hypothetical protein		-0.54	0.69	0.04
ABAYE0999	conserved hypothetical protein		-0.85	0.55	0.00
ABAYE1000	conserved hypothetical protein		-0.84	0.56	0.00
ABAYE1013	dual specificity pseudouridine synthase for 23S rRNA and tRNAphe modification	rluA	0.44	1.36	0.03
ABAYE1028	putative aldehyde dehydrogenase		1.30	2.46	0.03
ABAYE1030	putative pyruvate decarboxylase/indolepyruvate decarboxylase		1.50	2.83	0.00
ABAYE1042	putative Nudix hydrolase		0.39	1.31	0.01
ABAYE1045	conserved hypothetical protein%3B putative exported protein		1.10	2.14	0.00

ABAYE1046	conserved hypothetical protein%3B putative signal peptide		-0.77	0.59	0.01
ABAYE1048	lipoprotein precursor	nlpD	0.82	1.77	0.00
ABAYE1049	putative transcriptional regulator (LysR family)		-0.69	0.62	0.00
ABAYE1054	50S ribosomal protein L31	rpmE	-0.36	0.78	0.03
ABAYE1055	conserved hypothetical protein		-1.60	0.33	0.00
ABAYE1056	conserved hypothetical protein		-0.91	0.53	0.00
ABAYE1066	putative aldehyde dehydrogenase%3B putative betaine-aldehyde dehydrogenase		0.52	1.43	0.03
ABAYE1074	putative Prevent host death protein (Phd-like)		1.30	2.46	0.05
ABAYE1078	hypothetical protein%3B putative signal peptide		-0.43	0.74	0.03
ABAYE1079	hypothetical protein		-0.58	0.67	0.01
ABAYE1082	conserved hypothetical protein		0.42	1.34	0.04
ABAYE1086	putative acinetobactin biosynthesis protein (basA)		-0.64	0.64	0.03
ABAYE1087	putative non-ribosomal peptide synthetase with condensation and peptidyl carrier protein domains (basB)		-2.00	0.25	0.00
ABAYE1089	Ferric acinetobactin transport system permease protein	bauD	-1.20	0.44	0.00
ABAYE1090	Ferric acinetobactin transport system permease protein	bauC	-0.94	0.52	0.01
ABAYE1091	Ferric acinetobactin transport system ATP-binding protein	bauE	-1.90	0.27	0.00
ABAYE1092	Putative ferric acinetobactin binding protein (BauB)		-1.40	0.38	0.00

ABAYE1093	Putative ferric acinetobactin receptor (bauA)		-1.30	0.41	0.00
ABAYE1094	Putative acinetobactin siderophore biosynthesis protein (basC)		-0.47	0.72	0.03
ABAYE1097	2%2C3-dihydro-2%2C3-dihydroxybenzoate synthetase (isochorismatase) (N-terminal) with aryl carrier protein domain (C-terminal)	basF	-0.99	0.50	0.00
ABAYE1098	Histidine decarboxylase	hdc	-0.74	0.60	0.02
ABAYE1100	putative ABC transporter ATP-binding component (barA)		-0.62	0.65	0.01
ABAYE1101	putative ABC transporter (barB)		-0.83	0.56	0.00
ABAYE1102	putative thioesterase (basH)		-0.70	0.62	0.00
ABAYE1104	isochorismate synthetase	basJ	-0.47	0.72	0.04
ABAYE1108	conserved hypothetical protein%3B putative signal peptide		-1.00	0.50	0.00
ABAYE1109	conserved hypothetical protein%3B putative signal peptide		-0.50	0.71	0.05
ABAYE1122	putative aminopeptidase N(PEPN)		-0.40	0.76	0.03
ABAYE1124	putative peptidase%3B putative metallopeptidase		0.56	1.47	0.01
ABAYE1125	putative Amino acid/peptide transporter		0.83	1.78	0.01
ABAYE1136	HtrA-like serine protease	algW	0.31	1.24	0.04
ABAYE1144	putative 4-carboxymuconolactone decarboxylase		0.89	1.85	0.00
ABAYE1145	putative acyl-CoA dehydrogenase		0.68	1.60	0.00
ABAYE1151	conserved hypothetical protein%3B putative exported protein		2.20	4.59	0.00

ABAYE1154	protein chain elongation factor EF-Ts	tsf	-0.46	0.73	0.00
ABAYE1160	putative rare lipoprotein A family (RlpA-like)		0.67	1.59	0.00
ABAYE1172	putative Outer membrane efflux protein		0.49	1.40	0.01
ABAYE1173	putative Multidrug/solvent efflux pump membrane transporter		0.48	1.39	0.01
ABAYE1174	putative Multidrug/solvent efflux pump membrane transporter		0.58	1.49	0.00
ABAYE1176	putative transcriptional regulator (LysR family)		0.55	1.46	0.03
ABAYE1177	Lysine-arginine-ornithine-histidine-binding periplasmic protein precursor (ABC superfamily%2C peri_bind)	hisJ	-0.71	0.61	0.00
ABAYE1183	putative protease		-1.10	0.47	0.04
ABAYE1188	transposase of IS10A%2C IS4 family		0.93	1.91	0.00
ABAYE1189	fragment of conserved hypothetical protein (part 2)		1.10	2.14	0.00
ABAYE1191	putative secretion pathway ATPase		-2.80	0.14	0.00
ABAYE1192	conserved hypothetical protein%3B putative exported protein		3.20	9.19	0.00
ABAYE1193	putative two-component response regulator		1.40	2.64	0.00
ABAYE1194	putative two-component system sensor histidine kinase		0.98	1.97	0.00
ABAYE1197	conserved hypothetical protein%3B putative exported protein		0.65	1.57	0.00
ABAYE1198	bifunctional protein [Includes penicillin-insensitive transglycosylase%3B penicillin-sensitive transpeptidase] (penicillin-binding protein 1B)	mrcB	0.40	1.32	0.01
ABAYE1208	conserved hypothetical protein		0.32	1.25	0.03

ABAYE1216	conserved hypothetical protein%3B putative membrane protein		0.30	1.23	0.03
ABAYE1218	conserved hypothetical protein		-0.32	0.80	0.02
ABAYE1221	conserved hypothetical protein%3B putative exported protein		0.71	1.64	0.00
ABAYE1226	conserved hypothetical protein		0.27	1.21	0.04
ABAYE1227	hypothetical protein		0.40	1.32	0.03
ABAYE1230	conserved hypothetical protein		0.78	1.72	0.02
ABAYE1231	hypothetical protein		0.77	1.71	0.00
ABAYE1232	hypothetical protein		0.97	1.96	0.04
ABAYE1233	hypothetical protein		1.30	2.46	0.02
ABAYE1234	hypothetical protein%3B putative exported protein		2.40	5.28	0.00
ABAYE1237	hypothetical protein		1.80	3.48	0.00
ABAYE1239	hypothetical protein		0.68	1.60	0.01
ABAYE1240	conserved hypothetical protein%3B putative Phage replication protein		1.20	2.30	0.00
ABAYE1241	hypothetical protein		0.97	1.96	0.04
ABAYE1245	conserved hypothetical protein%3B putative phage related protein		1.00	2.00	0.00
ABAYE1246	hypothetical protein		1.10	2.14	0.02
ABAYE1253	hypothetical protein		-0.36	0.78	0.01
ABAYE1254	hypothetical protein		1.10	2.14	0.02

ABAYE1255	hypothetical protein		1.60	3.03	0.01
ABAYE1256	conserved hypothetical protein%3B putative phage related protein		0.84	1.79	0.02
ABAYE1257	hypothetical protein%3B putative exported protein		1.20	2.30	0.01
ABAYE1259	conserved hypothetical protein		0.79	1.73	0.00
ABAYE1260	hypothetical protein		1.60	3.03	0.00
ABAYE1261	conserved hypothetical protein%3B putative phage related protein		2.20	4.59	0.00
ABAYE1263	conserved hypothetical protein%3B putative phage related acetyltransferase		1.20	2.30	0.01
ABAYE1267	hypothetical protein		-1.20	0.44	0.00
ABAYE1270	hypothetical protein		1.90	3.73	0.00
ABAYE1273	conserved hypothetical protein%3B putative membrane protein		0.39	1.31	0.05
ABAYE1280	amidophosphoribosyltransferase	purF	-0.31	0.81	0.04
ABAYE1284	conserved hypothetical protein%3B putative exported protein		0.50	1.41	0.02
ABAYE1296	putative Methylmalonate-semialdehyde dehydrogenase (MmsA-like)		-0.62	0.65	0.04
ABAYE1297	putative amino acid transport protein		-0.68	0.62	0.04
ABAYE1298	conserved hypothetical protein		1.10	2.14	0.01
ABAYE1301	putative methyltransferase		0.89	1.85	0.00
ABAYE1303	conserved hypothetical protein%3B putative signal peptide		0.94	1.92	0.04
ABAYE1307	putative HTH-type transcriptional regulator (AraC family)		0.63	1.55	0.01

ABAYE1308	putative poly(R)-hydroxyalkanoic acid synthase		0.57	1.48	0.04
ABAYE1309	sodium/glutamate symport carrier protein	gltS	0.64	1.56	0.01
ABAYE1314	conserved hypothetical protein		0.48	1.39	0.02
ABAYE1315	conserved hypothetical protein%3B putative AAA ATPase		1.20	2.30	0.00
ABAYE1316	putative HTH-type Transcriptional regulator (GntR family)		1.20	2.30	0.00
ABAYE1318	hypothetical protein%3B putative transcriptional regulator (TetR family)		1.80	3.48	0.01
ABAYE1319	protein CsuA/B%3B putative secreted protein related to type I pili		1.50	2.83	0.00
ABAYE1320	protein CsuA		0.98	1.97	0.00
ABAYE1321	protein CsuB%3B putative secreted protein related to type I pili		0.86	1.82	0.01
ABAYE1322	protein CsuC%3B putative type I pilus usher pathway chaperone		1.10	2.14	0.00
ABAYE1323	protein CsuD%3B putative type I pili usher protein		1.20	2.30	0.00
ABAYE1324	protein CsuE%3B putative secreted protein related to type I pili		1.30	2.46	0.00
ABAYE1326	hypothetical protein		-0.38	0.77	0.04
ABAYE1327	conserved hypothetical protein		0.50	1.41	0.01
ABAYE1328	proline/betaine transporter (MFS superfamily)	proP	1.90	3.73	0.00
ABAYE1341	putative heavy metal sensor kinase		0.36	1.28	0.04
ABAYE1342	putative outer membrane lipoprotein (lipocalin)		0.69	1.61	0.01
ABAYE1352	conserved hypothetical protein%3B putative exported protein		0.51	1.42	0.04

ABAYE1353	putative acetyltransferase%3B putative modification of 30S ribosomal subunit protein S5 (RIMJ)(acetylation of N-terminal alanine)		-1.30	0.41	0.05
ABAYE1356	L-sorbosone dehydrogenase	sndH	1.00	2.00	0.00
ABAYE1357	putative acetyltransferase		1.30	2.46	0.00
ABAYE1372	fragment of conserved hypothetical protein (partial)		2.50	5.66	0.00
ABAYE1373	glucose-inhibited division protein A	gidA	0.38	1.30	0.03
ABAYE1377	conserved hypothetical protein		-0.36	0.78	0.04
ABAYE1386	cytochrome o ubiquinol oxidase subunit IV	cyoD	-0.27	0.83	0.02
ABAYE1387	cytochrome o ubiquinol oxidase subunit III	cyoC	-0.31	0.81	0.02
ABAYE1399	conserved hypothetical protein		0.63	1.55	0.01
ABAYE1408	putative binding-protein-dependent transport systems inner membrane component		0.50	1.41	0.02
ABAYE1411	putative 3-hydroxyacyl-CoA dehydrogenase		0.78	1.72	0.01
ABAYE1412	putative acyl-CoA dehydrogenase protein (acdB-like)		0.61	1.53	0.05
ABAYE1413	putative acetyl-CoA synthetase/AMP-(fatty) acid ligase		0.67	1.59	0.01
ABAYE1415	molybdopterin biosynthesis protein (moeB) OR thiamin-thiazole moiety synthesis (thiF)		0.85	1.80	0.00
ABAYE1424	regulator of kdp operon (transcriptional effector)	kdpE	0.43	1.35	0.01
ABAYE1432	aconitate hydratase 2	acnB	-0.34	0.79	0.01
ABAYE1433	hypothetical protein		-0.95	0.52	0.00

ABAYE1437	hypothetical protein%3B putative signal peptide		-2.00	0.25	0.00
ABAYE1438	conserved hypothetical protein%3B putative signal peptide		-1.20	0.44	0.01
ABAYE1439	transcriptional regulator	metR	-0.27	0.83	0.05
ABAYE1448	delta(2)-isopentenylpyrophosphate tRNA-adenosine transferase	miaA	0.34	1.27	0.04
ABAYE1450	putative integral membrane protein		0.55	1.46	0.01
ABAYE1457	Ethanolamine ammonia-lyase light chain (Ethanolamine ammonia-lyase small subunit)	eutC	0.81	1.75	0.01
ABAYE1459	putative transport protein		0.75	1.68	0.00
ABAYE1460	aldehyde dehydrogenase	ald1	1.20	2.30	0.00
ABAYE1461	putative transcriptional regulator		0.40	1.32	0.01
ABAYE1462	conserved hypothetical protein		0.37	1.29	0.01
ABAYE1463	putative alcohol dehydrogenase		1.70	3.25	0.00
ABAYE1464	putative helicase		0.23	1.17	0.05
ABAYE1479	hypothetical protein		1.00	2.00	0.01
ABAYE1480	hypothetical protein%3B putative signal peptide		-2.60	0.16	0.00
ABAYE1484	conserved hypothetical protein		-0.53	0.69	0.03
ABAYE1486	putative siderophore receptor		-0.82	0.57	0.03
ABAYE1487	putative transporter with mechanosensitive ion channel.		1.10	2.14	0.00

ABAYE1488	hypothetical protein		0.65	1.57	0.02
ABAYE1489	hypothetical protein		0.94	1.92	0.04
ABAYE1490	hypothetical protein		1.60	3.03	0.00
ABAYE1492	hypothetical protein%3B putative signal peptide		0.97	1.96	0.01
ABAYE1494	putative outer membrane porin%2C receptor for Fe(III)-coprogen%2C Fe(III)-ferrioxamine B and Fe(III)-rhodotrulic acid uptake (FhuE)		-0.70	0.62	0.00
ABAYE1498	conserved hypothetical protein		1.00	2.00	0.03
ABAYE1500	conserved hypothetical protein%3B putative stress protein		-0.73	0.60	0.03
ABAYE1503	conserved hypothetical protein%3B putative signal peptide		2.90	7.46	0.00
ABAYE1505	P-type ATPase%2C Mg2+ ATPase transporter	mgtA	1.10	2.14	0.00
ABAYE1507	putative benzoate membrane transport protein		-0.63	0.65	0.05
ABAYE1508	putative transcriptional regulator (AsnC family)%3B putative Leucine-responsive regulatory protein		0.53	1.44	0.00
ABAYE1512	conserved hypothetical protein%3B putative membrane protein		0.99	1.99	0.03
ABAYE1514	putative short-chain dehydrogenase		0.74	1.67	0.00
ABAYE1515	conserved hypothetical protein		0.43	1.35	0.00
ABAYE1516	conserved hypothetical protein		0.44	1.36	0.03
ABAYE1517	putative transcriptional regulator		0.71	1.64	0.00
ABAYE1518	methyl viologen resistance protein (MFS superfamily)	smvA	1.00	2.00	0.00

ABAYE1524	conserved hypothetical protein		1.10	2.14	0.00
ABAYE1529	putative glutamate synthase.		0.76	1.69	0.01
ABAYE1530	putative iron-regulated membrane protein		0.55	1.46	0.00
ABAYE1549	molybdopterin biosynthesis%2C protein A	moaA	0.47	1.39	0.01
ABAYE1550	conserved hypothetical protein		0.54	1.45	0.02
ABAYE1551	molybdopterin converting factor%2C large subunit	moaE	0.45	1.37	0.01
ABAYE1552	bifunctional protein [Includes: molybdenum cofactor biosynthesis protein C%3B molybdenum cofactor biosynthesis protein B]	moaCB	0.49	1.40	0.00
ABAYE1553	molybdopterin biosynthesis protein	moeA	0.34	1.27	0.03
ABAYE1561	putative intracellular sulfur oxidation protein (DsrE-like)		0.80	1.74	0.00
ABAYE1562	putative UDP-galactose 4-epimerase (GalE-like)		1.10	2.14	0.00
ABAYE1563	fumarase C (fumarate hydratase Class II)	fumC	0.74	1.67	0.00
ABAYE1564	hypothetical protein		0.42	1.34	0.01
ABAYE1565	hypothetical protein%3B putative signal peptide		-1.30	0.41	0.00
ABAYE1574	sensory kinase (soluble) in two-component regulatory system	glnL	-0.64	0.64	0.05
ABAYE1594	conserved hypothetical protein%3B putative signal peptide		-0.54	0.69	0.01
ABAYE1595	conserved hypothetical protein		-0.64	0.64	0.00
ABAYE1604	conserved hypothetical protein		0.88	1.84	0.00

ABAYE1605	Quinoprotein glucose dehydrogenase-B precursor (Glucose dehydrogenase-B [pyrroloquinoline-quinone]) (Soluble glucose dehydrogenase) (s-GDH)	gdhB	0.82	1.77	0.00
ABAYE1608	conserved hypothetical protein		0.46	1.38	0.01
ABAYE1611	conserved hypothetical protein%3B putative signal peptide		-4.00	0.06	0.00
ABAYE1621	methionine aminopeptidase	map	-0.75	0.59	0.00
ABAYE1622	putative transcriptional regulator (LysR family)		0.50	1.41	0.01
ABAYE1629	conserved hypothetical protein		0.51	1.42	0.04
ABAYE1633	conserved hypothetical protein		0.54	1.45	0.01
ABAYE1635	conserved hypothetical protein%3B putative membrane protein		-1.00	0.50	0.00
ABAYE1636	cytochrome d terminal oxidase polypeptide subunit II	cydB	-1.00	0.50	0.00
ABAYE1637	cytochrome d terminal oxidase%2C polypeptide subunit I	cydA	-1.20	0.44	0.00
ABAYE1657	conserved hypothetical protein%3B putative membrane protein		0.38	1.30	0.03
ABAYE1658	phospho-2-dehydro-3-deoxyheptonate aldolase	aro	-1.10	0.47	0.00
ABAYE1661	2%2C4-dienoyl-CoA reductase	fadH	0.52	1.43	0.04
ABAYE1671	putative cell division protein (FtsB-like)		0.39	1.31	0.01
ABAYE1672	2-C-methyl-D-erythritol 4-phosphate cytidylyltransferase	ispD	0.38	1.30	0.01
ABAYE1673	3-oxoadipate CoA-transferase subunit A (Beta-ketoadipate:succinyl-CoA transferase subunit A)	pcaI	0.56	1.47	0.04

ABAYE1680	protocatechuate 3%2C4-dioxygenase beta chain (3%2C4-PCD)	pcaH	0.85	1.80	0.02
ABAYE1681	protocatechuate 3%2C4-dioxygenase alpha chain (3%2C4-PCD)	pcaG	0.89	1.85	0.04
ABAYE1682	catabolic 3-dehydroquinate dehydratase (3-dehydroquinase)	quiB	1.10	2.14	0.00
ABAYE1683	3-dehydroshikimate dehydratase (DHS dehydratase) (DHSase)	quiC	0.46	1.38	0.02
ABAYE1686	conserved hypothetical protein%3B putative signal peptide		-0.73	0.60	0.00
ABAYE1687	conserved hypothetical protein%3B putative membrane protein		-0.62	0.65	0.05
ABAYE1688	conserved hypothetical protein%3B putative membrane protein		-0.57	0.67	0.01
ABAYE1700	putative Amidase		1.80	3.48	0.02
ABAYE1701	conserved hypothetical protein%3B putative Acyl-CoA dehydrogenase domain		2.40	5.28	0.00
ABAYE1702	conserved hypothetical protein		2.00	4.00	0.00
ABAYE1703	conserved hypothetical protein		2.00	4.00	0.00
ABAYE1704	putative aromatic-ring-hydroxylating dioxygenase large subunit		1.30	2.46	0.00
ABAYE1705	putative Aromatic-ring-hydroxylating dioxygenase small subunit		1.20	2.30	0.03
ABAYE1706	putative Short-chain dehydrogenase/reductase		1.80	3.48	0.01
ABAYE1707	putative Ferredoxin (Oxidoreductase FAD/NAD(P)-binding region)%3B putative aromatic oxidoreductase		0.88	1.84	0.02
ABAYE1710	Copper amine oxidase precursor (Tyramine oxidase) (2-phenylethylamine oxidase)	tynA	0.83	1.78	0.01
ABAYE1711	conserved hypothetical protein%3B putative signal peptide		1.20	2.30	0.03

ABAYE1712	phenylacetaldehyde dehydrogenase	feaB	0.87	1.83	0.02
ABAYE1714	3-oxoadipate enol-lactonase II (Beta-ketoadipate enol-lactone hydrolase II)	catD	0.45	1.37	0.04
ABAYE1715	beta-ketoadipyl CoA thiolase	catF	0.79	1.73	0.01
ABAYE1716	3-oxoadipate CoA-transferase subunit B (Beta-ketoadipate:succinyl-CoA transferase subunit B)	catJ	0.92	1.89	0.03
ABAYE1717	3-oxoadipate CoA-transferase subunit A (Beta-ketoadipate:succinyl-CoA transferase subunit A)	catI	0.64	1.56	0.02
ABAYE1718	catechol 1%2C2-dioxygenase	catA	0.72	1.65	0.00
ABAYE1719	muconolactone delta-isomerase (MIase)	catC	1.20	2.30	0.04
ABAYE1720	muconate cycloisomerase I (Cis%2Ccis-muconate lactonizing enzyme I) (MLE)	catB	0.39	1.31	0.04
ABAYE1728	putative NADP-dependent aldehyde dehydrogenase (AldH-like)		1.10	2.14	0.00
ABAYE1730	Dihydrocoumarin hydrolase	dch	0.71	1.64	0.00
ABAYE1732	conserved hypothetical protein%3B putative oxidoreductase		0.56	1.47	0.00
ABAYE1733	conserved hypothetical protein%3B putative signal peptide		0.64	1.56	0.01
ABAYE1736	putative oxidoreductase%3B putative Metal-binding Oxidoreductase		0.43	1.35	0.01
ABAYE1740	putative transcriptional regulator (AraC family)		-0.41	0.75	0.02
ABAYE1741	putative short chain dehydrogenase		1.90	3.73	0.00
ABAYE1746	L-sorbosone dehydrogenase	sndH	1.40	2.64	0.00

ABAYE1747	conserved hypothetical protein%3B putative membrane protein		1.40	2.64	0.01
ABAYE1749	putative Isochorismatase hydrolase		0.95	1.93	0.01
ABAYE1750	putative transcriptional regulator (LysR family)		0.71	1.64	0.00
ABAYE1751	Quinoprotein glucose dehydrogenase-B precursor (Glucose dehydrogenase-B [pyrroloquinoline-quinone]) (Soluble glucose dehydrogenase) (s-GDH)	gdhB	0.30	1.23	0.04
ABAYE1752	glutathione-regulated potassium-efflux system protein (K(+)/H(+) antiporter)	kef	0.47	1.39	0.02
ABAYE1753	hypothetical protein%3B putative signal peptide		0.84	1.79	0.01
ABAYE1757	hypothetical protein		-0.77	0.59	0.01
ABAYE1759	conserved hypothetical protein		-0.87	0.55	0.00
ABAYE1760	conserved hypothetical protein		-0.51	0.70	0.00
ABAYE1761	conserved hypothetical protein		-0.61	0.66	0.01
ABAYE1763	hypothetical protein%3B putative signal peptide		-0.80	0.57	0.03
ABAYE1768	putative Permease of the major facilitator		0.67	1.59	0.02
ABAYE1769	conserved hypothetical protein%3B putative metal-dependent hydrolase		0.73	1.66	0.00
ABAYE1770	conserved hypothetical protein%3B putative membrane protein		0.73	1.66	0.01
ABAYE1774	putative transcriptional regulator (LysR family)		0.72	1.65	0.00
ABAYE1777	putative Multidrug resistance efflux pump		0.45	1.37	0.03

ABAYE1778	putative Permease of the major facilitator superfamily%3B putative Multidrug efflux MFS transporter		0.58	1.49	0.02
ABAYE1781	putative Dihydroxy-acid and 6-phosphogluconate dehydratase (IlvD-Edd-like)		0.46	1.38	0.04
ABAYE1784	conserved hypothetical protein%3B putative NAD-dependent epimerase/dehydratase		0.68	1.60	0.01
ABAYE1785	putative Permease of the major facilitator%3B putative tartrate transporter		0.54	1.45	0.01
ABAYE1792	putative methylenetetrahydrofolate reductase		0.64	1.56	0.01
ABAYE1795	putative amidase		0.41	1.33	0.05
ABAYE1801	conserved hypothetical protein		0.53	1.44	0.03
ABAYE1805	conserved hypothetical protein%3B putative bifunctional protein [Includes :putative Restriction enzyme%3B putative helicase]		-0.47	0.72	0.01
ABAYE1818	hypothetical protein		0.54	1.45	0.02
ABAYE1821	membrane fusion protein	adeA	4.80	27.86	0.00
ABAYE1822	RND protein	adeB	3.80	13.93	0.00
ABAYE1823	outer membrane protein	adeC	4.60	24.25	0.00
ABAYE1824	conserved hypothetical protein		3.80	13.93	0.00
ABAYE1828	conserved hypothetical protein%3B putative membrane protein		0.51	1.42	0.04
ABAYE1830	hypothetical protein		-1.80	0.29	0.00
ABAYE1831	putative phage/plasmid replication protein		-1.40	0.38	0.00

ABAYE1833	hypothetical protein		-1.70	0.31	0.00
ABAYE1834	conserved hypothetical protein%3B putative phage-related protein		-0.74	0.60	0.00
ABAYE1835	conserved hypothetical protein%3B putative Phage-related protein		-0.68	0.62	0.03
ABAYE1836	hypothetical protein		-0.89	0.54	0.00
ABAYE1837	hypothetical protein%3B putative phage-related exported protein		-4.70	0.04	0.00
ABAYE1838	hypothetical protein		-4.20	0.05	0.00
ABAYE1839	hypothetical protein		-1.80	0.29	0.00
ABAYE1840	putative phage/plasmid replication protein		-1.50	0.35	0.00
ABAYE1846	hypothetical protein		-0.53	0.69	0.01
ABAYE1847	conserved hypothetical protein%3B putative phage-related protein		-0.50	0.71	0.02
ABAYE1849	hypothetical protein		-0.47	0.72	0.00
ABAYE1850	hypothetical protein%3B putative phage-related exported protein		-3.20	0.11	0.00
ABAYE1851	hypothetical protein		-3.40	0.09	0.00
ABAYE1855	hypothetical protein%3B putative exported protein		-1.80	0.29	0.00
ABAYE1856	putative fimbrial protein precursor (Pilin)		-2.20	0.22	0.00
ABAYE1857	putative pilin chaperone		-1.30	0.41	0.01
ABAYE1858	putative outer membrane usher protein		-1.40	0.38	0.01
ABAYE1859	putative Fimbria adhesin protein		-1.50	0.35	0.00

ABAYE1860	hypothetical protein%3B putative exported protein		-2.60	0.16	0.00
ABAYE1868	transposase of ISAba1%2C IS4 family (ORF 1)		-0.52	0.70	0.01
ABAYE1875	hypothetical protein		1.60	3.03	0.00
ABAYE1876	hypothetical protein		2.30	4.92	0.00
ABAYE1877	Zn-dependent dipeptidase	acdP	0.76	1.69	0.00
ABAYE1878	coenzyme PQQ synthesis protein E (Coenzyme PQQ synthesis protein III)	pqqE	0.90	1.87	0.00
ABAYE1879	coenzyme PQQ synthesis protein D (Coenzyme PQQ synthesis protein II)	pqqD	0.89	1.85	0.00
ABAYE1880	coenzyme PQQ synthesis protein C (Coenzyme PQQ synthesis protein I)	pqqC	0.72	1.65	0.00
ABAYE1881	coenzyme PQQ synthesis protein B (Coenzyme PQQ synthesis protein V)	pqqB	0.71	1.64	0.00
ABAYE1882	coenzyme PQQ synthesis protein A (Coenzyme PQQ synthesis protein IV)	pqqA	0.68	1.60	0.00
ABAYE1890	hypothetical protein		1.20	2.30	0.00
ABAYE1897	anthranilate dioxygenase small subunit	antB	1.70	3.25	0.01
ABAYE1902	conserved hypothetical protein		0.63	1.55	0.03
ABAYE1904	putative oxidoreductase%2C short-chain dehydrogenase/reductase family		1.60	3.03	0.00
ABAYE1906	fragment of putative carboxymethylenebutenolidase (Dienelactone hydrolase) (DLH) (part 1)		1.20	2.30	0.01
ABAYE1910	putative D-beta-hydroxybutyrate permease		0.60	1.52	0.01

ABAYE1913	acetoacetyl-CoA transferase%2C alpha subunit	atoD	0.56	1.47	0.02
ABAYE1914	acetoacetyl-CoA transferase%2C beta subunit	atoA	0.84	1.79	0.00
ABAYE1916	putative acetyl-CoA acetyltransferase (Acetoacetyl-CoA thiolase)		0.57	1.48	0.01
ABAYE1923	dicarboxylic acid transport protein%3B alpha-ketoglutarate permease (MFS superfamily)	pcaT	0.59	1.51	0.00
ABAYE1924	hypothetical protein		0.75	1.68	0.03
ABAYE1925	conserved hypothetical protein		0.65	1.57	0.00
ABAYE1928	putative transport protein (ABC superfamily%2C peri_bind)%3B putative nitrate ABC transporter%2C periplasmic		0.52	1.43	0.04
ABAYE1931	putative transcriptional regulator (GntR family)		1.00	2.00	0.03
ABAYE1934	putative transcriptional regulator		0.60	1.52	0.04
ABAYE1936	conserved hypothetical protein%3B putative membrane protein		-0.47	0.72	0.02
ABAYE1937	putative homoserine dehydrogenase (HDH)		-0.73	0.60	0.01
ABAYE1938	conserved hypothetical protein%3B putative membrane protein		-0.53	0.69	0.02
ABAYE1939	conserved hypothetical protein%3B putative NAD(FAD)-dependent dehydrogenase		-0.83	0.56	0.01
ABAYE1940	conserved hypothetical protein%3B putative Beta-lactamase-like protein		-0.87	0.55	0.00
ABAYE1943	putative (R%2CR)-butanediol dehydrogenase		0.49	1.40	0.00
ABAYE1945	dihydrolipoamide dehydrogenase (E3 component of pyruvate and 2-oxoglutarate dehydrogenases complexes)	acoD	0.75	1.68	0.00

ABAYE1946	dihydrolipoamide acetyltransferase	acoC	1.10	2.14	0.00
ABAYE1947	acetoin:2%2C6-dichlorophenolindophenol oxidoreductase beta subunit (Acetoin:DCPIP oxidoreductase-beta) (AO:DCPIP OR) (TPP-dependent acetoin dehydrogenase E1 beta-subunit)	acoB	1.30	2.46	0.00
ABAYE1948	acetoin:2%2C6-dichlorophenolindophenol oxidoreductase alpha subunit (Acetoin:DCPIP oxidoreductase-alpha) (AO:DCPIP OR)	acoA	1.40	2.64	0.00
ABAYE1949	lipoate synthase	lipA	0.39	1.31	0.03
ABAYE1950	putative transcriptional regulator%3B putative regulator of acetoin metabolisme		0.48	1.39	0.02
ABAYE1951	conserved hypothetical protein		-0.82	0.57	0.00
ABAYE1952	conserved hypothetical protein		-0.58	0.67	0.02
ABAYE1956	fragment of conserved hypothetical protein (part 2)		0.40	1.32	0.02
ABAYE1957	fragment of conserved hypothetical protein (part 1)		0.69	1.61	0.00
ABAYE1958	conserved hypothetical protein		0.41	1.33	0.02
ABAYE1959	conserved hypothetical protein%3B putative membrane protein		0.40	1.32	0.01
ABAYE1960	conserved hypothetical protein		0.30	1.23	0.03
ABAYE1962	conserved hypothetical protein		0.80	1.74	0.00
ABAYE1963	hypothetical protein		-0.59	0.66	0.01
ABAYE1964	hypothetical protein		-1.50	0.35	0.00
ABAYE1965	ribonuclease D%2C processes tRNA	rnd	-0.42	0.75	0.01

ABAYE1968	conserved hypothetical protein		0.61	1.53	0.00
ABAYE1969	hypothetical protein		1.40	2.64	0.03
ABAYE1971	putative poly(R)-hydroxyalkanoic acid synthase		0.47	1.39	0.02
ABAYE1973	hypothetical protein%3B putative exported protein		-0.81	0.57	0.00
ABAYE1975	conserved hypothetical protein		0.85	1.80	0.00
ABAYE1979	putative porin precursor		0.47	1.39	0.01
ABAYE1980	conserved hypothetical protein		0.91	1.88	0.00
ABAYE1981	conserved hypothetical protein		0.38	1.30	0.05
ABAYE1982	conserved hypothetical protein		0.27	1.21	0.05
ABAYE1988	conserved hypothetical protein%3B putative membrane protein		0.92	1.89	0.00
ABAYE1991	conserved hypothetical protein%3B putative exported protein		0.47	1.39	0.02
ABAYE1996	putative hydrolase%2C haloacid dehalogenase-like family		0.43	1.35	0.03
ABAYE1999	conserved hypothetical protein%3B putative membrane protein		-0.79	0.58	0.00
ABAYE2001	putative ferric siderophore receptor protein		-1.70	0.31	0.00
ABAYE2003	conserved hypothetical protein		-0.43	0.74	0.02
ABAYE2004	conserved hypothetical protein%3B putative siderophore biosynthesis protein		-0.58	0.67	0.00
ABAYE2005	conserved hypothetical protein		-0.59	0.66	0.00
ABAYE2007	putative lysine/ornithine N-monooxygenase		-0.90	0.54	0.03

ABAYE2011	conserved hypothetical protein%3B putative exported protein		0.28	1.21	0.02
ABAYE2017	DNA-binding protein HU-beta	hupB	-0.32	0.80	0.03
ABAYE2034	conserved hypothetical protein%3B putative exported protein		0.40	1.32	0.01
ABAYE2038	conserved hypothetical protein		-0.37	0.77	0.03
ABAYE2047	putative TonB protein		-0.69	0.62	0.02
ABAYE2057	conserved hypothetical protein%3B putative membrane protein		-0.24	0.85	0.05
ABAYE2059	conserved hypothetical protein%3B putative exported protein		0.59	1.51	0.00
ABAYE2060	integration host factor (IHF)%2Cbeta subunit%2C site specific recombination	himD	0.48	1.39	0.01
ABAYE2061	30S ribosomal protein S1	rpsA	-0.46	0.73	0.02
ABAYE2073	conserved hypothetical protein		-0.40	0.76	0.00
ABAYE2074	type 4 fimbrial biogenesis protein	pilZ	-0.87	0.55	0.00
ABAYE2075	DNA polymerase III%2C delta prime subunit	holB	-0.32	0.80	0.03
ABAYE2078	lipid transport protein%2C flippase (ABC superfamily%2C membrane (N-terminal)%2C atp_bind (C-terminal))	msbA	-0.23	0.85	0.03
ABAYE2080	putative biopolymer transport protein (EXBB-like)		-0.56	0.68	0.02
ABAYE2087	peptidyl-prolyl cis-trans isomerase	surA	0.25	1.19	0.05
ABAYE2092	conserved hypothetical protein		0.34	1.27	0.05
ABAYE2096	putative transcriptional regulator (ArsR family)		1.30	2.46	0.00

ABAYE2100	putative transport protein (ABC superfamily%2C ATP_bind)		0.35	1.27	0.03
ABAYE2101	conserved hypothetical protein		0.35	1.27	0.03
ABAYE2104	NAD-dependent deacetylase (Regulatory protein sirtuin family)	npdA	0.45	1.37	0.03
ABAYE2106	leucine-responsive regulatory protein	lrp	0.51	1.42	0.02
ABAYE2114	conserved hypothetical protein%3B putative exported protein		0.39	1.31	0.05
ABAYE2122	Carbapenem-hydrolyzing oxacillinase OXA-69 (Beta-lactamase OXA-69)	bla-OXA-69	1.10	2.14	0.00
ABAYE2131	conserved hypothetical protein		-0.92	0.53	0.00
ABAYE2132	putative fimbrial protein precursor (Pilin)		-3.10	0.12	0.00
ABAYE2133	putative pilin chaperone		-1.80	0.29	0.00
ABAYE2134	fragment of putative outer membrane usher protein (part 1)		-0.88	0.54	0.01
ABAYE2136	transposase of ISAba1%2C IS4 family (ORF 1)		-0.61	0.66	0.00
ABAYE2140	hypothetical protein%3B putative exported protein		1.20	2.30	0.01
ABAYE2141	putative transcriptional regulator (LysR family)		0.55	1.46	0.00
ABAYE2149	putative phage integrase		-0.58	0.67	0.00
ABAYE2151	conserved hypothetical protein		-0.64	0.64	0.00
ABAYE2152	putative transcriptional regulator (TetR family)		-1.20	0.44	0.00
ABAYE2168	D-methionine transport protein (ABC superfamily%2C peri_bind)	metQ	0.32	1.25	0.04

ABAYE2174	conserved hypothetical protein%3B putative exported protein		-0.90	0.54	0.00
ABAYE2178	conserved hypothetical protein		-1.10	0.47	0.00
ABAYE2183	putative transcriptional regulator (AraC family)		0.49	1.40	0.01
ABAYE2187	putative glutamate symport transmembrane protein		0.95	1.93	0.01
ABAYE2190	putative tRNA/rRNA methyltransferase		0.37	1.29	0.02
ABAYE2191	serine acetyltransferase	cysE	0.27	1.21	0.03
ABAYE2192	conserved hypothetical protein%3B putative exported protein		0.39	1.31	0.01
ABAYE2195	putative chromate transport protein		1.00	2.00	0.00
ABAYE2200	Arsenate reductase		1.80	3.48	0.00
ABAYE2201	fragment of conserved hypothetical protein		1.30	2.46	0.01
ABAYE2208	hydantoin-racemase	hyu	0.65	1.57	0.02
ABAYE2209	Alpha-ketoglutarate-dependent taurine dioxygenase (2-aminoethanesulfonate dioxygenase) (Sulfate starvation-induced protein 3) (SSI3)	tauD	0.51	1.42	0.01
ABAYE2216	putative acyl-CoA dehydrogenase		0.91	1.88	0.00
ABAYE2217	conserved hypothetical protein		1.50	2.83	0.00
ABAYE2219	ubiquinol oxidase subunit I%2C cyanide insensitive	cioA	1.60	3.03	0.00
ABAYE2220	ubiquinol oxidase subunit II%2C cyanide insensitive	cioB	1.70	3.25	0.00
ABAYE2223	putative bifunctional protein [Includes: putative transcriptional regulator (GntR		0.54	1.45	0.00

	family)%3B putative amino transferase]				
ABAYE2227	malonate decarboxylase%2C epsilon subunit (acyl carrier transferase)	mdcH	0.61	1.53	0.05
ABAYE2228	phosphoribosyl-dephospho-CoA transferase (Holo-ACP synthase)	mdcG	0.51	1.42	0.02
ABAYE2229	malonate decarboxylase%2C gamma subunit	mdcE	0.78	1.72	0.00
ABAYE2235	putative tetracycline resistance protein		-0.76	0.59	0.00
ABAYE2237	conserved hypothetical protein%3B putative negative transcriptional regulator		0.66	1.58	0.01
ABAYE2244	conserved hypothetical protein%3B putative glutathione S-transferase enzyme with thioredoxin-like domain		0.63	1.55	0.00
ABAYE2245	putative transcriptional regulator (LysR family)		0.51	1.42	0.03
ABAYE2253	putative amino acid transport protein (ABC superfamily%2C peri_bind)		-0.34	0.79	0.03
ABAYE2256	putative amino acid transport protein (ABC superfamily%2C membrane)		0.56	1.47	0.02
ABAYE2258	conserved hypothetical protein%3B putative acetyltransferase		0.77	1.71	0.00
ABAYE2260	putative two-component sensor kinase		-0.60	0.66	0.03
ABAYE2265	fragment of RecA-dependent DNA damage-inducible protein (partial)	ddrR	0.85	1.80	0.03
ABAYE2267	hypothetical protein		0.75	1.68	0.00
ABAYE2268	hypothetical protein		2.30	4.92	0.00
ABAYE2269	putative Short-chain dehydrogenase/reductase		0.39	1.31	0.03
ABAYE2270	Catalase hydroperoxidase II	katE	1.30	2.46	0.00

ABAYE2271	conserved hypothetical protein		1.00	2.00	0.01
ABAYE2276	hypothetical protein		1.50	2.83	0.02
ABAYE2277	hypothetical protein%3B putative exported protein		2.00	4.00	0.00
ABAYE2281	conserved hypothetical protein		-1.70	0.31	0.00
ABAYE2282	putative transcriptional regulator (LysR family)		-1.10	0.47	0.00
ABAYE2285	conserved hypothetical protein%3B putative SAM-dependent methyltransferase		0.99	1.99	0.00
ABAYE2286	conserved hypothetical protein%3B putative AMP-dependent synthetase and ligase		0.90	1.87	0.00
ABAYE2287	putative transcriptional regulator (TetR family)		0.61	1.53	0.01
ABAYE2289	conserved hypothetical protein%3B putative Carboxyl transferase domain		1.10	2.14	0.01
ABAYE2290	3-methylglutaconyl-CoA hydratase	mgh	1.60	3.03	0.00
ABAYE2291	conserved hypothetical protein%3B putative Biotin carboxylase		1.60	3.03	0.00
ABAYE2292	putative Hydroxymethylglutaryl-CoA lyase (HMG-CoA lyase) (HL) (3-hydroxy-3-methylglutarate-CoA lyase)(MvaB-like)		1.60	3.03	0.00
ABAYE2295	conserved hypothetical protein		0.57	1.48	0.02
ABAYE2302	transport protein in catabolism of dicarboxylic acids (MFS superfamily)	dcaK	0.58	1.49	0.02
ABAYE2303	acyl-CoA dehydrogenase	dcaA	0.92	1.89	0.03
ABAYE2304	enoyl-CoA hydratase	dcaE	1.20	2.30	0.01
ABAYE2305	oxidoreductase%3B putative ketoacyl-CoA reductase	dcaC	1.20	2.30	0.01

ABAYE2306	dehydrogenase	dcaH	0.76	1.69	0.04
ABAYE2307	beta-ketoadipyl CoA thiolase	dcaF	0.95	1.93	0.04
ABAYE2309	putative transport protein (MFS superfamily)		0.69	1.61	0.00
ABAYE2310	conserved hypothetical protein%3B putative 2-nitropropane dioxygenase		1.10	2.14	0.01
ABAYE2311	conserved hypothetical protein%3B putative Enoyl-CoA hydratase/isomerase		0.84	1.79	0.05
ABAYE2312	transcriptional regulator (IclR family)	dcaS	0.53	1.44	0.03
ABAYE2314	L-carnitine dehydrogenase	caiB	0.90	1.87	0.01
ABAYE2315	acyl-CoA dehydrogenase	dcaA	1.90	3.73	0.00
ABAYE2316	cis%2Ccis-muconate transport protein (MFS superfamily)	mucK	0.64	1.56	0.04
ABAYE2318	2-aminoethylphosphonate:pyruvate transaminasee	phnW	0.75	1.68	0.00
ABAYE2319	putative FAD dependent oxidoreductase		0.98	1.97	0.03
ABAYE2320	putative transport protein (ABC superfamily%2C membrane)		1.30	2.46	0.01
ABAYE2325	p-hydroxybenzoate hydroxylase transcriptional activator	pobR	1.10	2.14	0.01
ABAYE2327	putative transcriptional regulator (LysR family)		0.65	1.57	0.02
ABAYE2330	conserved hypothetical protein%3B putative exported protein		2.10	4.29	0.00
ABAYE2331	putative purine cytosine permease		1.10	2.14	0.00
ABAYE2333	putative aldehyde dehydrogenase		0.74	1.67	0.04
ABAYE2335	conserved hypothetical protein		1.20	2.30	0.01

ABAYE2336	conserved hypothetical protein		1.20	2.30	0.04
ABAYE2337	conserved hypothetical protein		1.20	2.30	0.01
ABAYE2342	conserved hypothetical protein%3B putative Catalase domain		0.60	1.52	0.00
ABAYE2345	conserved hypothetical protein%3B putative acyl-CoA transferase/carnitine dehydratase		0.55	1.46	0.04
ABAYE2354	hypothetical protein		2.10	4.29	0.00
ABAYE2355	hypothetical protein		0.65	1.57	0.01
ABAYE2361	putative helicase		-0.78	0.58	0.00
ABAYE2362	hypothetical protein%3B putative endonuclease		-0.86	0.55	0.00
ABAYE2378	putative amino acid transporter%3B putative L-serine transporter		0.54	1.45	0.00
ABAYE2379	NADP-dependent fatty aldehyde dehydrogenase	aldH	0.84	1.79	0.00
ABAYE2388	putative Dihydrodipicolinate synthetase		0.93	1.91	0.00
ABAYE2392	conserved hypothetical protein		-1.70	0.31	0.00
ABAYE2393	putative acetyltransferase		-0.87	0.55	0.00
ABAYE2395	putative cyanate transport protein (CYNX)(MFS family)		0.47	1.39	0.00
ABAYE2399	conserved hypothetical protein%3B putative exported protein		-0.55	0.68	0.01
ABAYE2400	conserved hypothetical protein%3B putative exported protein		-0.69	0.62	0.00
ABAYE2401	conserved hypothetical protein		-0.74	0.60	0.00

ABAYE2402	conserved hypothetical protein		-0.86	0.55	0.00
ABAYE2403	conserved hypothetical protein		-0.75	0.59	0.00
ABAYE2404	putative ClpA/B-type chaperone		-0.92	0.53	0.00
ABAYE2405	conserved hypothetical protein		-1.10	0.47	0.00
ABAYE2406	putative outer membrane lipoprotein		-1.20	0.44	0.00
ABAYE2407	conserved hypothetical protein		-1.00	0.50	0.00
ABAYE2408	conserved hypothetical protein%3B putative membrane protein		-1.30	0.41	0.00
ABAYE2409	conserved hypothetical protein		-1.40	0.38	0.00
ABAYE2410	conserved hypothetical protein		-1.30	0.41	0.00
ABAYE2411	conserved hypothetical protein		-4.60	0.04	0.00
ABAYE2412	conserved hypothetical protein		-5.70	0.02	0.00
ABAYE2413	conserved hypothetical protein		-8.20	0.00	0.00
ABAYE2414	conserved hypothetical protein		-7.50	0.01	0.00
ABAYE2415	conserved hypothetical protein		-6.40	0.01	0.00
ABAYE2416	conserved hypothetical protein		-6.00	0.02	0.00
ABAYE2418	conserved hypothetical protein		-5.60	0.02	0.00
ABAYE2419	putative transport protein (ABC superfamily%2C membrane)		-4.30	0.05	0.00
ABAYE2420	putative transport protein (ABC superfamily%2C atp_bind)		-3.00	0.13	0.00

ABAYE2421	putative transport protein (ABC superfamily%2C peri_bind)		-3.40	0.09	0.00
ABAYE2422	putative amidase		-3.60	0.08	0.00
ABAYE2423	putative transcriptional regulator		-1.10	0.47	0.01
ABAYE2425	putative HTH-type transcriptional regulator		-2.20	0.22	0.00
ABAYE2426	conserved hypothetical protein%3B putative NAD(P)H dehydrogenase (quinone)		-3.30	0.10	0.00
ABAYE2427	putative Biotin carboxyl carrier protein		-1.20	0.44	0.04
ABAYE2428	putative multifonctional protein [Includes: putative Carbamoyl-phosphate synthase L chain%3B putative Biotin carboxylase%3B putative Allophanate hydrolase subunit 2 and 1]		-4.70	0.04	0.00
ABAYE2429	conserved hypothetical protein		-1.50	0.35	0.00
ABAYE2430	conserved hypothetical protein		-2.70	0.15	0.00
ABAYE2431	putative zinc-type alcohol dehydrogenase-like protein		-2.50	0.18	0.00
ABAYE2432	conserved hypothetical protein		-5.10	0.03	0.00
ABAYE2434	hypothetical protein%3B putative exported protein		-6.80	0.01	0.00
ABAYE2435	hypothetical protein%3B putative exported protein		-7.00	0.01	0.00
ABAYE2436	conserved hypothetical protein		-6.10	0.01	0.00
ABAYE2437	conserved hypothetical protein%3B putative chloride transport protein		-5.70	0.02	0.00
ABAYE2438	Acetyl-/propionyl-coenzyme A carboxylase alpha chain [Includes: Biotin carboxylase %3B Biotin carboxyl carrier protein (BCCP)]	bccA	-5.00	0.03	0.00

ABAYE2439	putative allophanate hydrolase subunit 1 and 2		-4.10	0.06	0.00
ABAYE2440	conserved hypothetical protein		-3.60	0.08	0.00
ABAYE2441	putative lactam utilization protein		-3.90	0.07	0.00
ABAYE2442	conserved hypothetical protein%3B putative membrane protein		-4.10	0.06	0.00
ABAYE2443	putative transcriptional regulator (LysR family)		-4.70	0.04	0.00
ABAYE2444	hypothetical protein%3B putative exported protein		-5.00	0.03	0.00
ABAYE2445	hypothetical protein		-5.10	0.03	0.00
ABAYE2446	hypothetical protein		-4.90	0.03	0.00
ABAYE2447	hypothetical protein		-4.40	0.05	0.00
ABAYE2449	hypothetical protein		-3.90	0.07	0.00
ABAYE2450	fragment of putative Rhs family protein (part 2)		-5.30	0.03	0.00
ABAYE2452	transposase of ISAba1%2C IS4 family (ORF 1)		-0.96	0.51	0.00
ABAYE2453	fragment of putative Rhs family protein (part 1)		-7.90	0.00	0.00
ABAYE2454	conserved hypothetical protein		-8.40	0.00	0.00
ABAYE2456	putative beta-lactamase		-5.50	0.02	0.00
ABAYE2457	putative acetyltransferase		-3.00	0.13	0.00
ABAYE2458	hypothetical protein		-3.70	0.08	0.00
ABAYE2459	hypothetical protein		-2.80	0.14	0.00

ABAYE2460	putative hydroxyacyl-CoA dehydrogenase		-5.00	0.03	0.00
ABAYE2461	conserved hypothetical protein		-3.70	0.08	0.00
ABAYE2463	hypothetical protein%3B putative exported protein		-7.10	0.01	0.00
ABAYE2464	conserved hypothetical protein%3B putative exported protein		-6.20	0.01	0.00
ABAYE2465	putative Permease of the major facilitator superfamily		-5.00	0.03	0.00
ABAYE2468	putative lipid A biosynthesis acyltransferase		-6.40	0.01	0.00
ABAYE2469	hypothetical protein		-3.20	0.11	0.00
ABAYE2470	hypothetical protein%3B putative membrane protein		-4.00	0.06	0.00
ABAYE2471	conserved hypothetical protein%3B putative membrane protein		-3.60	0.08	0.00
ABAYE2476	methyl-directed mismatch repair%2C recognize exocyclic adducts of guanosine	mutS	0.26	1.20	0.03
ABAYE2480	conserved hypothetical protein%3B putative exported protein		0.50	1.41	0.01
ABAYE2484	conserved hypothetical protein		-0.40	0.76	0.05
ABAYE2497	hypothetical protein		-1.30	0.41	0.00
ABAYE2498	conserved hypothetical protein%3B putative exported protein		-0.92	0.53	0.00
ABAYE2501	hypothetical protein%3B putative exported protein		-0.82	0.57	0.00
ABAYE2503	hypothetical protein%3B putative exported protein		0.70	1.62	0.00
ABAYE2504	fragment of conserved hypothetical protein		0.72	1.65	0.00

ABAYE2506	conserved hypothetical protein		0.73	1.66	0.00
ABAYE2508	conserved hypothetical protein		-0.86	0.55	0.01
ABAYE2509	hypothetical protein		-0.90	0.54	0.01
ABAYE2516	hypothetical protein		0.73	1.66	0.00
ABAYE2519	hypothetical protein		1.20	2.30	0.00
ABAYE2520	hypothetical protein%3B putative exported protein		1.10	2.14	0.00
ABAYE2522	hypothetical protein		-0.50	0.71	0.02
ABAYE2525	hypothetical protein%3B putative exported protein		1.60	3.03	0.00
ABAYE2530	hypothetical protein		-0.54	0.69	0.01
ABAYE2532	hypothetical protein%3B putative exported protein		0.95	1.93	0.00
ABAYE2536	conserved hypothetical protein%3B putative Amino acid efflux-like protein		-0.83	0.56	0.00
ABAYE2539	hypothetical protein		1.10	2.14	0.04
ABAYE2546	conserved hypothetical protein		0.45	1.37	0.04
ABAYE2548	conserved hypothetical protein		0.65	1.57	0.00
ABAYE2557	benzoate 1%2C2-dioxygenase electron transfer component [Includes: Ferredoxin%3B Ferredoxin--NAD(+) reductase]	benC	1.10	2.14	0.00
ABAYE2558	cis-1%2C2-dihydroxycyclohexa-3%2C 5-diene-1-carboxylate dehydrogenase%2C(cis-1%2C2-dihydroxy-3%2C 4-cyclohexadiene-1-carboxylate dehydrogenase) (2-hydro-1%2C2-dihydroxybenzoate dehydrogenase) (DHB dehydrogenase)	benD	0.89	1.85	0.00

ABAYE2560	benzoate transport protein (MFS superfamily)	benK	0.47	1.39	0.03
ABAYE2566	O-methyl transferase	mdmC	0.26	1.20	0.04
ABAYE2567	conserved hypothetical protein		0.60	1.52	0.02
ABAYE2569	conserved hypothetical protein%3B putative exported protein		0.85	1.80	0.00
ABAYE2571	putative extracellular nuclease		0.26	1.20	0.05
ABAYE2575	conserved hypothetical protein%3B putative bacterial outer membrane domain		0.66	1.58	0.01
ABAYE2585	putative peptidase%2C M23/M37 family		0.45	1.37	0.00
ABAYE2586	putative acyltransferase%3B acyltransferase for phosphonate utilization (PhnO)		0.70	1.62	0.00
ABAYE2588	cyclic AMP receptor protein	vfr	-0.58	0.67	0.02
ABAYE2589	putative oxidoreductase%2C aldo/keto reductase family		0.58	1.49	0.01
ABAYE2594	D-erythrose 4-phosphate dehydrogenase	epd	0.31	1.24	0.02
ABAYE2600	putative cytochrome		0.52	1.43	0.03
ABAYE2602	putative transporter		-0.32	0.80	0.05
ABAYE2604	conserved hypothetical protein%3B putative exported protein		-1.70	0.31	0.00
ABAYE2608	conserved hypothetical protein		0.67	1.59	0.01
ABAYE2616	putative transcritional regulator		0.56	1.47	0.02
ABAYE2618	putative short-chain dehydrogenase		0.79	1.73	0.04
ABAYE2619	putative Acetyl esterase		0.81	1.75	0.04

ABAYE2621	putative porin for vanillate trafficking (VanP)		0.55	1.46	0.01
ABAYE2622	vanillate transporter (MFS superfamily)	vanK	1.00	2.00	0.00
ABAYE2623	vanillate O-demethylase oxygenase subunit (4-hydroxy-3-methoxybenzoate demethylase)	vanA	1.90	3.73	0.00
ABAYE2624	vanillate O-demethylase oxidoreductase (Vanillate degradation ferredoxin-like protein)	vanB	0.59	1.51	0.02
ABAYE2634	conserved hypothetical protein		1.60	3.03	0.00
ABAYE2636	transposase of ISAba1%2C IS4 family (ORF 1)		-0.37	0.77	0.01
ABAYE2643	transposase of ISAba1%2C IS4 family (ORF 1)		-0.37	0.77	0.04
ABAYE2645	fragment of conserved hypothetical protein%3B putative membrane protein		-0.60	0.66	0.01
ABAYE2649	transposase of ISAba1%2C IS4 family (ORF 2)		-0.46	0.73	0.02
ABAYE2651	hypothetical protein%3B putative membrane protein		-1.00	0.50	0.01
ABAYE2652	hypothetical protein%3B putative Rhs family protein		-0.80	0.57	0.01
ABAYE2653	hypothetical protein%3B putative Rhs family protein		-0.79	0.58	0.02
ABAYE2654	hypothetical protein%3B putative exported protein		-0.68	0.62	0.03
ABAYE2656	hypothetical protein%3B putative exported protein		-0.79	0.58	0.01
ABAYE2665	conserved hypothetical protein		-2.40	0.19	0.00
ABAYE2666	putative NADH pyrophosphatase (NUDIX hydrolase family)(NudC)		-0.41	0.75	0.00
ABAYE2668	conserved hypothetical protein%3B putative exported protein		0.52	1.43	0.00

ABAYE2672	inhibitor of replication initiation (transcriptional regulator of dnaA and argK (affects arginine transport) (LysR family))	iciA	-0.35	0.78	0.05
ABAYE2676	conserved hypothetical protein		-0.46	0.73	0.04
ABAYE2682	conserved hypothetical protein		0.42	1.34	0.01
ABAYE2689	conserved hypothetical protein		0.74	1.67	0.02
ABAYE2690	hypothetical protein%3B putative membrane protein		1.30	2.46	0.04
ABAYE2695	hypothetical protein%3B putative acetyltransferase%2C GNAT family		-0.51	0.70	0.04
ABAYE2699	hypothetical protein		-0.85	0.55	0.00
ABAYE2700	hypothetical protein%3B putative exported protein		-0.91	0.53	0.00
ABAYE2712	hypothetical protein		0.58	1.49	0.02
ABAYE2730	conserved hypothetical protein		0.63	1.55	0.02
ABAYE2731	conserved hypothetical protein		0.84	1.79	0.02
ABAYE2732	conserved hypothetical protein		1.30	2.46	0.01
ABAYE2733	hypothetical protein		1.20	2.30	0.03
ABAYE2735	hypothetical protein%3B putative exported protein		1.40	2.64	0.04
ABAYE2736	fragment of conserved hypothetical protein		0.99	1.99	0.04
ABAYE2738	hypothetical protein		1.10	2.14	0.00
ABAYE2741	conserved hypothetical protein		1.00	2.00	0.02

ABAYE2742	putative replicative DNA helicase		0.76	1.69	0.01
ABAYE2751	conserved hypothetical protein		0.71	1.64	0.01
ABAYE2752	hypothetical protein%3B putative phage-related protein		0.78	1.72	0.01
ABAYE2763	conserved hypothetical protein%3B putative exported protein		1.50	2.83	0.00
ABAYE2777	urease beta subunit	ureB	-0.36	0.78	0.04
ABAYE2779	urease accessory protein	ureD	-0.70	0.62	0.02
ABAYE2782	putative lipoprotein		1.30	2.46	0.00
ABAYE2783	isocitrate lyase	aceA	-0.93	0.52	0.00
ABAYE2787	citrate transporter	citN	0.45	1.37	0.04
ABAYE2791	sulfate adenylyltransferase subunit 2 (Sulfate adenylate transferase) (SAT) (ATP-sulfurylase small subunit)	cysD	0.34	1.27	0.04
ABAYE2792	conserved hypothetical protein		0.49	1.40	0.03
ABAYE2793	conserved hypothetical protein%3B putative exported protein		0.38	1.30	0.04
ABAYE2802	conserved hypothetical protein%3B putative signal peptide		-0.77	0.59	0.03
ABAYE2805	conserved hypothetical protein%3B putative membrane protein		-0.32	0.80	0.02
ABAYE2807	putative transport protein (ABC superfamily%2C atp_bind)		0.57	1.48	0.01
ABAYE2808	multifunctional protein [Includes: acyl-CoA thioesterase I%3B protease I%3B lysophospholipaseL(I)]	tesA	0.40	1.32	0.03
ABAYE2810	lipase	lip1	0.28	1.21	0.05

ABAYE2812	ferric enterobactin receptor precursor	pfeA	-2.00	0.25	0.00
ABAYE2816	conserved hypothetical protein%3B putative exported protein		-1.60	0.33	0.00
ABAYE2817	conserved hypothetical protein%3B putative lipoprotein precursor		-0.96	0.51	0.02
ABAYE2823	putative transketolase C-terminal section		0.75	1.68	0.00
ABAYE2824	putative transketolase N-terminal section		0.54	1.45	0.03
ABAYE2829	Aldose 1-epimerase precursor (Mutarotase)		1.10	2.14	0.00
ABAYE2830	fosmidomycin/multidrug transport protein (MFS superfamily)	fsr	0.38	1.30	0.01
ABAYE2831	putative transcriptional regulator (AraC family)		-0.36	0.78	0.05
ABAYE2832	putative Glutaminase		-0.82	0.57	0.00
ABAYE2834	putative permease (MFS superfamily)		0.64	1.56	0.00
ABAYE2835	conserved hypothetical protein%3B putative exported protein		0.71	1.64	0.00
ABAYE2836	putative acetolactate synthase (IlvB-like)		0.91	1.88	0.00
ABAYE2837	putative aldehyde dehydrogenase		1.30	2.46	0.00
ABAYE2838	putative L-aspartate dehydrogenase (NadX)		1.30	2.46	0.00
ABAYE2839	putative short-chain dehydrogenase		0.91	1.88	0.01
ABAYE2840	putative hydrolase		0.81	1.75	0.02
ABAYE2841	conserved hypothetical protein		1.20	2.30	0.02
ABAYE2842	putative permease (MFS superfamily)		0.89	1.85	0.03

ABAYE2844	putative dioxygenase		0.73	1.66	0.00
ABAYE2846	putative vanillate O-demethylase oxygenase subunit (VanA-like)		0.70	1.62	0.02
ABAYE2847	conserved hypothetical protein		1.70	3.25	0.00
ABAYE2851	putative nucleoside/purine/pyrimidine transport protein (NMN family) (PnuC)		0.56	1.47	0.04
ABAYE2855	putative lipoprotein (Haemin storage system) (HmsF)		-0.61	0.66	0.01
ABAYE2859	conserved hypothetical protein		0.72	1.65	0.00
ABAYE2861	conserved hypothetical protein%3B putative exported protein		3.50	11.31	0.00
ABAYE2863	putative membrane protein		0.88	1.84	0.00
ABAYE2864	high-affinity choline transporter (BCCT family)	betT	0.83	1.78	0.00
ABAYE2865	putative choline/carnitine/betaine transporter family protein		1.10	2.14	0.00
ABAYE2866	repressor of bet genes	betI	0.85	1.80	0.02
ABAYE2867	NAD+-dependent betaine aldehyde dehydrogenase	betB	0.86	1.82	0.05
ABAYE2868	choline dehydrogenase%2C a flavoprotein	betA	0.70	1.62	0.04
ABAYE2869	malate dehydrogenase%2C FAD/NAD(P)-binding domain	mqo	-0.36	0.78	0.04
ABAYE2880	hypothetical protein%3B putative exported protein		1.30	2.46	0.03
ABAYE2881	hypothetical protein		0.87	1.83	0.02
ABAYE2883	conserved hypothetical protein		-1.00	0.50	0.00
ABAYE2884	putative TatD-related deoxyribonuclease from bacteriophage		-0.62	0.65	0.00

ABAYE2885	conserved hypothetical protein from bacteriophage		-1.20	0.44	0.00
ABAYE2886	conserved hypothetical protein from bacteriophage		-1.30	0.41	0.00
ABAYE2887	conserved hypothetical protein from bacteriophage		-1.70	0.31	0.00
ABAYE2891	hypothetical protein%3B putative membrane protein from bacteriophage		-0.81	0.57	0.00
ABAYE2892	putative nucleoid-associated protein from bacteriophage		-0.84	0.56	0.00
ABAYE2894	hypothetical protein from bacteriophage		-0.67	0.63	0.02
ABAYE2895	hypothetical protein from bacteriophage		-0.64	0.64	0.02
ABAYE2899	conserved hypothetical protein from bacteriophage		-1.00	0.50	0.00
ABAYE2900	hypothetical protein from bacteriophage		-0.73	0.60	0.00
ABAYE2901	conserved hypothetical protein from bacteriophage		-0.96	0.51	0.00
ABAYE2902	hypothetical protein from bacteriophage		-0.78	0.58	0.03
ABAYE2904	putative site-specific recombinase%2C integrase from bacteriophage		-0.40	0.76	0.03
ABAYE2905	gamma-glutamyltranspeptidase precursor	ggt	0.31	1.24	0.04
ABAYE2918	twitching motility protein	pilT	-3.30	0.10	0.00
ABAYE2919	twitching motility protein	pilU	-2.90	0.13	0.00
ABAYE2924	conserved hypothetical protein%3B putative Hemerythrin-like protein		-2.30	0.20	0.00
ABAYE2928	phosphomannomutase	algC	0.35	1.27	0.03
ABAYE2931	putative outer membrane protein		0.59	1.51	0.00

ABAYE2933	conserved hypothetical protein		0.64	1.56	0.01
ABAYE2934	cell division inhibitor%2C inhibits ftsZ ring formation	minC	0.36	1.28	0.01
ABAYE2935	cell division inhibitor%2C a membrane ATPase%2Cactivates minC	minD	0.27	1.21	0.03
ABAYE2937	conserved hypothetical protein		0.61	1.53	0.01
ABAYE2945	conserved hypothetical protein		0.48	1.39	0.00
ABAYE2946	protein chain elongation factor EF-Tu (duplicate of tufB)	tufA	-0.65	0.64	0.00
ABAYE2947	protein chain elongation factor EF-G%2C GTP-binding	fusA	-0.58	0.67	0.00
ABAYE2949	30S ribosomal protein S12	rpsL	-0.24	0.85	0.03
ABAYE2957	putative dioxygenase beta subunit		1.00	2.00	0.00
ABAYE2958	Succinate-semialdehyde dehydrogenase [NADP+] (SSDH)	gabD	1.20	2.30	0.00
ABAYE2959	fragment of putative dioxygenase alpha subunit (part 2)		1.10	2.14	0.00
ABAYE2960	transposase of IS10A%2C IS4 family		0.96	1.95	0.00
ABAYE2961	fragment of putative dioxygenase alpha subunit (part 1)		0.66	1.58	0.02
ABAYE2962	putative Lipase		1.30	2.46	0.00
ABAYE2963	putative BCCT family transporter		1.00	2.00	0.01
ABAYE2964	tartrate dehydrogenase/decarboxylase (TDH) (D-malate dehydrogenase [decarboxylating])		0.92	1.89	0.00
ABAYE2965	putative transcriptional regulator (LysR family)		0.73	1.66	0.00

ABAYE2966	conserved hypothetical protein%3B putative exported protein		-0.32	0.80	0.02
ABAYE2978	outer-membrane lipoprotein precursor	lolB	0.31	1.24	0.04
ABAYE2988	putative hydrolase		0.30	1.23	0.02
ABAYE2990	conserved hypothetical protein		0.90	1.87	0.00
ABAYE3001	dethiobiotin synthetase (Dethiobiotin synthase) (DTB synthetase) (DTBS)	bioD	0.55	1.46	0.03
ABAYE3002	putative biotin biosynthesis protein (BioC)		0.70	1.62	0.00
ABAYE3003	8-amino-7-oxononanoate synthase	bioF	0.60	1.52	0.00
ABAYE3004	adenosylmethionine-8-amino-7-oxononanoate aminotransferase	bioA	0.43	1.35	0.01
ABAYE3007	trehalose-6-phosphate synthase	otsA	0.67	1.59	0.01
ABAYE3008	putative transport protein (permease)		0.75	1.68	0.01
ABAYE3011	putative cell division protein (ZipA-like)		0.33	1.26	0.04
ABAYE3030	conserved hypothetical protein		1.60	3.03	0.00
ABAYE3034	putative transcriptional regulator (TetR family)		0.76	1.69	0.01
ABAYE3035	putative transporter (MFS superfamily)		0.51	1.42	0.01
ABAYE3036	putative multidrug resistance efflux pump		0.57	1.48	0.01
ABAYE3040	conserved hypothetical protein%3B putative membrane protein		1.60	3.03	0.00
ABAYE3041	conserved hypothetical protein		1.10	2.14	0.00
ABAYE3061	putative membrane protein%3B putative GGDEF domain		-0.44	0.74	0.03

ABAYE3063	putative sensory histidine kinase in two-component regulatory system		0.42	1.34	0.01
ABAYE3068	putative outermembrane protein exposed to the bacterial surface		2.00	4.00	0.00
ABAYE3072	hypothetical protein		-1.00	0.50	0.00
ABAYE3078	putative flavoprotein oxidoreductase		-1.10	0.47	0.00
ABAYE3079	5-methyltetrahydropteroyltriglutamate-homocysteine methyltransferase	metE	-1.40	0.38	0.00
ABAYE3080	conserved hypothetical protein		-1.60	0.33	0.00
ABAYE3083	putative amino acid efflux protein (LysE family)		1.00	2.00	0.00
ABAYE3086	putative hydrolase		-0.40	0.76	0.02
ABAYE3089	citrate-proton symporter (MFS superfamily)	citA	0.52	1.43	0.02
ABAYE3090	putative substrate-binding protein (ABC superfamily%2C peri-bind)		1.00	2.00	0.05
ABAYE3092	conserved hypothetical protein		0.67	1.59	0.01
ABAYE3093	citrate utilization protein B	citB	0.79	1.73	0.01
ABAYE3097	glutaryl-CoA dehydrogenase	gcdH	0.50	1.41	0.04
ABAYE3115	conserved hypothetical protein		0.56	1.47	0.01
ABAYE3118	conserved hypothetical protein		0.49	1.40	0.02
ABAYE3144	organic hydroperoxide resistance protein	ohr	0.77	1.71	0.01
ABAYE3170	putative AMP-dependent synthetase/ligase		0.46	1.38	0.00
ABAYE3178	putative DNA uptake protein and/or related DNA-binding protein		-0.99	0.50	0.00

ABAYE3194	putative membrane protein		0.43	1.35	0.04
ABAYE3195	conserved hypothetical protein		-1.00	0.50	0.00
ABAYE3196	putative transport protein (MFS superfamily)		-0.39	0.76	0.03
ABAYE3208	conserved hypothetical protein		0.90	1.87	0.00
ABAYE3217	hypothetical protein		-0.74	0.60	0.00
ABAYE3218	hypothetical protein		0.36	1.28	0.04
ABAYE3219	hypothetical protein		0.32	1.25	0.04
ABAYE3223	tRNA-dihydrouridine synthase A	dusA	0.47	1.39	0.03
ABAYE3224	conserved hypothetical protein		1.10	2.14	0.00
ABAYE3225	putative transcriptional regulator (LysR family)		0.30	1.23	0.02
ABAYE3227	putative enzyme NAD(P)-binding		0.46	1.38	0.01
ABAYE3228	aconitate hydratase 1	acnA	0.88	1.84	0.00
ABAYE3233	conserved hypothetical protein		-2.00	0.25	0.00
ABAYE3234	putative transcriptional regulator (TetR family)		-1.60	0.33	0.00
ABAYE3235	putative transcriptional regulator (TetR family)		-1.10	0.47	0.01
ABAYE3243	conserved hypothetical protein%3B putative exported protein		-0.80	0.57	0.01
ABAYE3251	conserved hypothetical protein%3B putative membrane protein		-0.64	0.64	0.00
ABAYE3253	conserved hypothetical protein		0.36	1.28	0.01

ABAYE3257	molecular chaperone in protein export	secB	-0.29	0.82	0.04
ABAYE3258	putative 4-phosphopantetheinyl transferase		0.40	1.32	0.01
ABAYE3271	carboxy-terminal protease for penicillin-binding protein	prc	0.31	1.24	0.02
ABAYE3272	beta-N-acetyl-D-glucosaminidase	nagZ	0.40	1.32	0.02
ABAYE3273	conserved hypothetical protein		0.31	1.24	0.03
ABAYE3277	putative NADP-dependent glyceraldehyde-3-phosphate dehydrogenase		0.49	1.40	0.02
ABAYE3278	thermoresistant gluconokinase (Gluconate kinase)	gntK	0.39	1.31	0.03
ABAYE3279	high-affinity gluconate permease (GntP family)	gntT	0.56	1.47	0.00
ABAYE3280	bifunctional protein [Includes: 4-hydroxy-2-oxoglutarate aldolase (2-keto-4-hydroxyglutarate aldolase) (KHG-aldolase)%3B 2-dehydro-3-deoxyphosphogluconate aldolase (Phospho-2-dehydro-3-deoxygluconate aldolase) (Phospho-2-keto-3-deoxygluconate aldolase) (2-keto-3-deoxy-6-phosphogluconate aldolase) (KDPG-aldolase)]	eda	0.35	1.27	0.03
ABAYE3281	phosphogluconate dehydratase (6-phosphogluconate dehydratase)	edd	0.55	1.46	0.00
ABAYE3283	phosphate acetyltransferase	pta	0.55	1.46	0.03
ABAYE3293	homoserine O-acetyltransferase	metX	0.23	1.17	0.05
ABAYE3294	methionine biosynthesis protein	metW	0.24	1.18	0.04
ABAYE3302	transposase of ISAba1%2C IS4 family (ORF 1)		-0.53	0.69	0.00
ABAYE3309	conserved hypothetical protein		0.49	1.40	0.02
ABAYE3311	prolipoprotein diacylglyceryl transferase	lgt	0.42	1.34	0.00

ABAYE3317	peptide methionine sulfoxide reductase (Protein-methionine-S-oxide reductase) (Peptide Met(O) reductase)	msrA	0.44	1.36	0.01
ABAYE3318	putative biopolymer transport protein (ExbD)		-0.60	0.66	0.05
ABAYE3319	putative biopolymer transport protein (ExbB)		-0.80	0.57	0.03
ABAYE3330	conserved hypothetical protein		0.45	1.37	0.01
ABAYE3334	conserved hypothetical protein%3B putative signal peptide		-0.88	0.54	0.00
ABAYE3348	aspartate-semialdehyde dehydrogenase%2C NAD(P)-binding	asd	-0.36	0.78	0.05
ABAYE3349	conserved hypothetical protein%3B putative signal peptide		-1.20	0.44	0.00
ABAYE3350	conserved hypothetical protein		-2.70	0.15	0.00
ABAYE3356	conserved hypothetical protein		-1.20	0.44	0.00
ABAYE3374	23S rRNA pseudouridylate synthase	rluC	0.39	1.31	0.04
ABAYE3389	conserved hypothetical protein%3B putative exported protein		0.44	1.36	0.05
ABAYE3393	ferrochelatase	hemH	-0.39	0.76	0.03
ABAYE3394	conserved hypothetical protein		-0.89	0.54	0.01
ABAYE3399	small ubiquitous protein required for normal growth	sirA	0.50	1.41	0.01
ABAYE3410	putative amino-acid efflux transmembrane protein		-0.76	0.59	0.00
ABAYE3417	conserved hypothetical protein %3B putative Penicillin-binding protein%2C transpeptidase fold		0.60	1.52	0.04
ABAYE3421	conserved hypothetical protein		0.45	1.37	0.01

ABAYE3427	putative oxidoreductase%3B flavoprotein monooxygenase		-0.64	0.64	0.02
ABAYE3444	type 4 fimbrial biogenesis protein	pilB	-3.20	0.11	0.00
ABAYE3445	type 4 fimbrial assembly protein	pilC	-2.80	0.14	0.00
ABAYE3446	type 4 prepilin-like proteins leader peptide processing enzyme (Protein secretion protein XCPA)[Includes: Leader peptidase (Prepilin peptidase)%3B N-methyltransferase]	pilD	-1.50	0.35	0.00
ABAYE3447	dephosphocoenzyme A kinase	coaE	-0.44	0.74	0.00
ABAYE3451	conserved hypothetical protein		-0.33	0.80	0.05
ABAYE3453	protein used in recombination and DNA repair	recN	-0.28	0.82	0.04
ABAYE3466	conserved hypothetical protein		0.83	1.78	0.03
ABAYE3468	conserved hypothetical protein%3B putative exported protein		-2.30	0.20	0.00
ABAYE3472	conserved hypothetical protein%3B putative exported protein		0.57	1.48	0.01
ABAYE3479	conserved hypothetical protein		-0.53	0.69	0.02
ABAYE3481	conserved hypothetical protein%3B putative exported protein		0.80	1.74	0.00
ABAYE3486	conserved hypothetical protein%3B putative exported protein		0.92	1.89	0.01
ABAYE3487	conserved hypothetical protein%3B putative exported protein		1.70	3.25	0.00
ABAYE3490	50S ribosomal protein L7/L12	rplL	-0.36	0.78	0.00
ABAYE3496	protein chain elongation factor EF-Tu (duplicate of tufA)	tufB	-0.40	0.76	0.02
ABAYE3518	hydroxymethylpyrimidine moiety synthesis in thiamin biosynthesis	thiC	0.37	1.29	0.03

ABAYE3519	adenosine diphosphate sugar pyrophosphatase (ADP-ribose pyrophosphatase)	aspP	-0.63	0.65	0.01
ABAYE3525	conserved hypothetical protein%3B putative exported protein		-0.66	0.63	0.04
ABAYE3528	site-specific tyrosine recombinase	xerD	-0.75	0.59	0.00
ABAYE3535	type 4 fimbriae expression regulatory protein	pilR	-1.70	0.31	0.00
ABAYE3538	conserved hypothetical protein%3B putative membrane protein		0.28	1.21	0.03
ABAYE3550	conserved hypothetical protein		-0.36	0.78	0.02
ABAYE3551	fragment of magnesium chelatase%2C competence related protein (ComM)		-0.63	0.65	0.00
ABAYE3562	hypothetical protein		0.92	1.89	0.00
ABAYE3565	conserved hypothetical protein		0.89	1.85	0.00
ABAYE3566	hypothetical protein		0.81	1.75	0.00
ABAYE3567	conserved hypothetical protein		0.72	1.65	0.00
ABAYE3570	Aminoglycoside 3-(9)-O-adenylyltransferase AADA1	aadA1	0.59	1.51	0.02
ABAYE3571	putative GCN5-related N-acetyltransferase%2C OrfX		0.44	1.36	0.01
ABAYE3572	putative GCN5-related N-acetyltransferase%2C OrfX		0.36	1.28	0.04
ABAYE3580	transposase of IS15DI%2C IS6 family		0.36	1.28	0.02
ABAYE3581	Resolvase	tnpR	0.45	1.37	0.01
ABAYE3582	Transposase		0.47	1.39	0.02

ABAYE3583_1	transposase of IS26%2C IS6 family	tnpA	0.42	1.34	0.03
ABAYE3588	putative GCN5-related N-acetyltransferase		0.49	1.40	0.00
ABAYE3589	hypothetical protein		0.97	1.96	0.00
ABAYE3590	hypothetical protein		1.10	2.14	0.00
ABAYE3591	conserved hypothetical protein		0.98	1.97	0.00
ABAYE3592	hypothetical protein		1.00	2.00	0.00
ABAYE3593	conserved hypothetical protein%3B putative Isochorismatase hydrolase		1.10	2.14	0.00
ABAYE3596	conserved hypothetical protein%3B putative membrane protein (PecM-like)		0.87	1.83	0.00
ABAYE3598	tetracycline repressor protein	tetR	0.47	1.39	0.01
ABAYE3599	putative Relaxase/helicase		0.52	1.43	0.01
ABAYE3601	Mercuric resistance operon regulatory protein	merR	0.65	1.57	0.01
ABAYE3602	fragment of conserved hypothetical protein (part 2)		0.97	1.96	0.00
ABAYE3603	fragment of conserved hypothetical protein (part 1)		0.84	1.79	0.03
ABAYE3604	Mercury resistance inner membrane protein	merC	0.61	1.53	0.04
ABAYE3605	Mercuric reductase (Hg(II) reductase)	merA	0.91	1.88	0.00
ABAYE3606	HTH-type transcriptional regulator (Mercuric resistance protein merD)	merD	0.85	1.80	0.01
ABAYE3607	mercuric resistance protein	merE	1.00	2.00	0.00
ABAYE3610	conserved hypothetical protein		0.64	1.56	0.00

ABAYE3611	conserved hypothetical protein		0.89	1.85	0.01
ABAYE3619	Beta-lactamase OXA-10 precursor	oxa-10	-0.42	0.75	0.03
ABAYE3623	Extended spectrum beta-lactamase VEB-1	veb-1	-0.36	0.78	0.01
ABAYE3624	transposase of IS10A%2C IS4 family		0.32	1.25	0.01
ABAYE3627	GroEL/integrase fusion protein		0.33	1.26	0.05
ABAYE3633	GroEL/integrase fusion protein		0.48	1.39	0.01
ABAYE3636	putative transcriptional regulator (LysR family)		0.65	1.57	0.01
ABAYE3637	Tetracycline resistance protein%2C class G (TETA(G))	tetA	0.52	1.43	0.00
ABAYE3639	Tetracycline repressor protein class G	tetR	0.43	1.35	0.03
ABAYE3647	Streptomycin 3-kinase (Streptomycin 3-phosphotransferase) (SPH)	strB	0.53	1.44	0.03
ABAYE3648	Streptomycin 3-kinase (Streptomycin 3-phosphotransferase) (Streptomycin 6-phosphotransferase) (Streptomycin 6-kinase)	aphE	0.48	1.39	0.02
ABAYE3649	fragment of Streptomycin 3-adenylyltransferase		0.65	1.57	0.02
ABAYE3671	putative Dihydrodipicolinate synthase (DapA)		-0.49	0.71	0.02
ABAYE3685	putative transport protein (MFS superfamily)		0.35	1.27	0.01
ABAYE3686	putative lysozyme		0.33	1.26	0.02
ABAYE3690	conserved hypothetical protein		0.75	1.68	0.00
ABAYE3693	conserved hypothetical protein%3B putative membrane protein		1.20	2.30	0.00

ABAYE3694	conserved hypothetical protein%3B putative flavodoxin		0.92	1.89	0.00
ABAYE3695	conserved hypothetical protein		1.20	2.30	0.00
ABAYE3696	subunit of cysteine synthase A and O-acetylserine sulfhydrolase A%2C PLP-dependent enzyme	cysK	-0.30	0.81	0.04
ABAYE3697	putative acetyltransferase		-0.50	0.71	0.02
ABAYE3701	conserved hypothetical protein		0.48	1.39	0.02
ABAYE3702	fragment of conserved hypothetical protein (partial)		0.61	1.53	0.01
ABAYE3703	putative outer membrane copper receptor (OprC)		0.76	1.69	0.00
ABAYE3704	conserved hypothetical protein%3B putative exported protein		0.49	1.40	0.01
ABAYE3706	conserved hypothetical protein%3B putative exported protein		-2.20	0.22	0.00
ABAYE3707	putative Rossmann-fold nucleotide-binding protein involved in DNA uptake (Smf)		-1.80	0.29	0.00
ABAYE3708	putative ribosome maturation factor (dsRNA-binding protein) (RimN)		-0.28	0.82	0.03
ABAYE3715	membrane-bound ATP synthase %2C F1 sector%2C epsilon-subunit	atpC	-0.34	0.79	0.01
ABAYE3719	membrane-bound ATP synthase %2C F1 sector%2C delta-subunit	atpH	-0.37	0.77	0.01
ABAYE3720	membrane-bound ATP synthase%2C F0 sector%2C subunit b	atpF	-0.36	0.78	0.01
ABAYE3734	glutathione S-transferase		0.37	1.29	0.05
ABAYE3743	putative integral membrane protein (DedA)		0.32	1.25	0.02
ABAYE3744	putative membrane protein		0.39	1.31	0.04

ABAYE3745	conserved hypothetical protein%3B putative exported protein		0.65	1.57	0.01
ABAYE3746	putative ATPase involved in chromosome partitioning (ParA family ATPase)		0.44	1.36	0.01
ABAYE3753	putative efflux pump membrane transporter (RND superfamily)		-1.20	0.44	0.00
ABAYE3754	putative Non-ribosomal peptide synthetase		-1.80	0.29	0.00
ABAYE3755	putative Phosphopantetheine binding protein		-1.30	0.41	0.02
ABAYE3756	putative acyl-CoA dehydrogenase		-2.20	0.22	0.00
ABAYE3757	putative non-ribosomal peptide synthase%3B AMP-dependent synthetase and ligase domain		-2.10	0.23	0.00
ABAYE3758	putative Autoinducer-binding transcriptional regulator (LuxR family)		-0.59	0.66	0.02
ABAYE3761	N-acylhomoserine lactone synthase%2C autoinducer synthesis protein		-1.10	0.47	0.00
ABAYE3762	putative transport protein (MFS family)		0.46	1.38	0.04
ABAYE3766	putative acetyl-coA synthetase/AMP-(fatty) acid ligase		0.59	1.51	0.00
ABAYE3770	APC family%2C D-serine/D-alanine/glycine transport protein	cycA	-0.54	0.69	0.01
ABAYE3771	APC family%2C D-serine/D-alanine/glycine transport protein	cycA	-0.59	0.66	0.04
ABAYE3777	putative ferric siderophore receptor protein		-0.29	0.82	0.04
ABAYE3780	putative dual specificity pseudouridine synthase for 23S rRNA and tRNAphe modification (RluA-like)		0.49	1.40	0.01
ABAYE3783	putative Short-chain dehydrogenase/reductase SDR		-3.20	0.11	0.00
ABAYE3789	conserved hypothetical protein		-0.53	0.69	0.01

ABAYE3791	putative methyl-cis-aconitic acid hydratase (AcnM)		0.67	1.59	0.01
ABAYE3792	methylcitrate synthase (citrate synthase 2)	prpC	0.72	1.65	0.01
ABAYE3793	methylisocitrate lyase	prpB	0.42	1.34	0.02
ABAYE3794	putative transcriptional regulator (GntR family)		0.54	1.45	0.00
ABAYE3796	D-lactate dehydrogenase%2C NADH independent%2C FAD-binding domain	dld	-0.77	0.59	0.00
ABAYE3797	L-lactate dehydrogenase%2C FMN linked	lldD	-1.20	0.44	0.00
ABAYE3798	transcriptional repressor for L-lactate utilization (GntR family)	lldR	-1.10	0.47	0.00
ABAYE3799	L-lactate permease	lldP	-1.60	0.33	0.00
ABAYE3816	polysaccharide export protein	wza	0.44	1.36	0.01
ABAYE3817	low molecular weight protein-tyrosine-phosphatase	ptp	0.51	1.42	0.00
ABAYE3818	tyrosine-protein kinase%2C autophosphorylates	ptk	0.42	1.34	0.01
ABAYE3822	N-acetyl-anhydromuramyl-L-alanine amidase (Regulates ampC)	ampD	-0.53	0.69	0.00
ABAYE3825	phospholipase C precursor (PLC) (Phosphatidylcholine cholinephosphohydrolase) (Phosphatidylcholine-hydrolyzing phospholipase C) (PC-PLC)	plc	-0.38	0.77	0.03
ABAYE3831	conserved hypothetical protein		0.27	1.21	0.04
ABAYE3838	conserved hypothetical protein%3B putative exported protein		-1.40	0.38	0.01
ABAYE3839	N-alpha-acetylglutamate synthase (amino-acid acetyltransferase)	argA	-0.61	0.66	0.00

ABAYE3845	putative transcriptional repressor (TetR/AcrR family)		-0.68	0.62	0.00
ABAYE3846	putative 5-methylthioadenosine/S-adenosylhomocysteine nucleosidase		-0.55	0.68	0.01
ABAYE3848	putative malic acid transport protein		-0.46	0.73	0.00
ABAYE3861	conserved hypothetical protein%3B putative exported protein		3.70	13.00	0.00
ABAYE3864	Hsp 24 nucleotide exchange factor	grpE	-0.64	0.64	0.03
ABAYE3866	hypothetical protein%3B putative peptidase M10A and M12B%2C matrixin and adamalysin%3B putative signal peptide		-0.98	0.51	0.00
ABAYE3867	hypothetical protein%3B putative signal peptide		-1.30	0.41	0.01
ABAYE3868	conserved hypothetical protein		-0.60	0.66	0.02
ABAYE3874	conserved hypothetical protein%3B putative membrane protein		-0.40	0.76	0.03
ABAYE3877	UDP-N-acetylmuramate:L-alanyl-gamma-D-glutamyl-meso-diaminopimelate ligase	mpl	0.40	1.32	0.00
ABAYE3888	conserved hypothetical protein%3B putative membrane protein		0.35	1.27	0.01
ABAYE3891	transposase of ISAba1%2C IS4 family (ORF 1)		-0.49	0.71	0.03
ABAYEtRNA21	ABAYEtRNA21		1.20	2.30	0.05
ABAYEtRNA24	ABAYEtRNA24		0.44	1.36	0.03
ABAYEtRNA30	ABAYEtRNA30		0.57	1.48	0.04
ABAYEtRNA5	ABAYEtRNA5		0.39	1.31	0.02
ABAYEtRNA6	ABAYEtRNA6		0.49	1.40	0.00

ABAYEtRNA7	ABAYEtRNA7		0.62	1.54	0.00
ABAYEtRNA72	ABAYEtRNA72		0.86	1.82	0.01
