# Supplementary material for: The Acinetobacter baumannii Two-Component System AdeRS Regulates Genes Required for Multidrug Efflux, Biofilm Formation, and Virulence in a Strain-Specific Manner
Source: mBio. 2016 Apr 19;7(2):e00430-16. doi: 10.1128/mBio.00430-16 (PMC4850262; doi:10.1128/mBio.00430-16)
Supplement: Table S3 — Gene expression data from the complete transcriptome analysis of A. baumannii S1 by RNA-Seq, showing differentially expressed genes (P < 0.05) in AYEΔadeAB compared with S1. [file mbo002162774st3.pdf]

Table S3 Gene expression data from the complete transcriptome analysis of *A. baumannii* S1 by RNA-Seq, showing differentially expressed genes (P < 0.05) in AYEΔ*adeAB* compared with S1.

ABAYE Gene ID	Annotation	Gene Name	log2 Fold Change	Fold Change	P.Value
ABAYE0005	putative of Cytochrome b(562) (CybC)		-0.25	0.84	0.05
ABAYE0024	conserved hypothetical protein		-0.11	0.93	0.01
ABAYE0073	histidine utilization repressor	hutC	0.22	1.16	0.00
ABAYE0080	conserved hypothetical protein%3B putative signal peptide		-0.31	0.81	0.01
ABAYE0129	putative flavoprotein monooxygenase acting on aromatic compound		0.54	1.45	0.01
ABAYE0138	thiol:disulfide interchange protein precursor	dsbD	-0.14	0.91	0.02
ABAYE0145	putative ferric siderophore receptor protein		-0.28	0.82	0.04
ABAYE0148	UDP-N-acetylglucosamine:N-acetylmuramyl-(pentapeptide) pyrophosphoryl-undecaprenol N-acetylglucosamine transferase	murG	-0.18	0.88	0.02
ABAYE0165	putative ferredoxin		-0.90	0.54	0.01
ABAYE0181	putative transcriptional regulator (Lrp-like)		2.30	4.92	0.00
ABAYE0198	conserved hypothetical protein%3B putative membrane protein		0.32	1.25	0.03
ABAYE0207	gamma-aminobutyrate permease	gabP	0.95	1.93	0.00
ABAYE0209	4-aminobutyrate aminotransferase%2C PLP-dependent	gabT	1.20	2.30	0.00

ABAYE0210	NADP+-dependent succinate semialdehyde dehydrogenase	gabD	0.99	1.99	0.02
ABAYE0229	conserved hypothetical protein		0.52	1.43	0.02
ABAYE0240	water channel (aquaporin Z) (MIP family)	aqpZ	0.32	1.25	0.00
ABAYE0247	putative membrane protein		-0.37	0.77	0.05
ABAYE0250	phosphoribosylformimino-5-aminoimidazole carboxamide isomerase	hisA	-0.32	0.80	0.01
ABAYE0257	putative acetyl-CoA hydrolase/transferase		-0.26	0.84	0.02
ABAYE0262	putative sulfate permease		0.28	1.21	0.03
ABAYE0290	putative membrane protein (ComM)		-0.79	0.58	0.01
ABAYE0291	putative membrane protein (ComN)		-0.94	0.52	0.02
ABAYE0292	putative membrane protein (ComO)		-0.95	0.52	0.02
ABAYE0293	putative lipoprotein (ComL)		-0.96	0.51	0.01
ABAYE0294	putative outer membrane protein (ComQ)		-1.10	0.47	0.02
ABAYE0297	conserved hypothetical protein		-0.10	0.93	0.04
ABAYE0317	putative type IV fimbrial biogenesis protein		-0.94	0.52	0.03
ABAYE0318	putative competence factor involved in DNA binding and uptake (ComC)		-0.76	0.59	0.03
ABAYE0320	pilin like competence factor	comF	-0.62	0.65	0.04
ABAYE0381	1-deoxyxylulose-5-phosphate synthase	dxs	-0.10	0.93	0.02
ABAYE0413	50S ribosomal protein L22	rplV	0.10	1.07	0.02

ABAYE0506	conserved hypothetical protein		-0.81	0.57	0.02
ABAYE0519	conserved hypothetical protein		0.28	1.21	0.01
ABAYE0523	DNA repair protein%2C associated with replication forks	radC	-0.28	0.82	0.03
ABAYE0587	putative glycosyl transferase		1.90	3.73	0.00
ABAYE0611	conserved hypothetical protein		0.24	1.18	0.01
ABAYE0614	acetylCoA carboxylase%2C beta subunit	accD	-0.17	0.89	0.05
ABAYE0632	putative glucose-sensitive porin (OprB-like)		0.18	1.13	0.04
ABAYE0666	conserved hypothetical protein%3B putative exported protein		-0.52	0.70	0.01
ABAYE0669	twitching motility protein	pilI	-0.87	0.55	0.03
ABAYE0670	type IV pilus biogenesis protein	pilJ	-0.98	0.51	0.03
ABAYE0671	putative sensor histidine kinase/response regulator%3B putative protein of chemotactic signal transduction system (ChA-like)		-1.20	0.44	0.02
ABAYE0674	putative Oxygen-independent coproporphyrinogen III oxidase		1.80	3.48	0.00
ABAYE0750	conserved hypothetical protein%3B putative membrane protein		-0.17	0.89	0.04
ABAYE0773	citrate synthase	gltA	-0.06	0.96	0.04
ABAYE0790	conserved hypothetical protein		0.17	1.13	0.04
ABAYE0796	putative methyltransferase		0.19	1.14	0.02
ABAYE0816	glycerol kinase	glpk	-0.26	0.84	0.02

ABAYE0844	hypothetical protein		1.00	2.00	0.05
ABAYE0869	conserved hypothetical protein%3B putative membrane protein		0.46	1.38	0.04
ABAYE1045	conserved hypothetical protein%3B putative exported protein		0.14	1.10	0.02
ABAYE1048	lipoprotein precursor	nlpD	0.23	1.17	0.05
ABAYE1055	conserved hypothetical protein		-0.66	0.63	0.02
ABAYE1056	conserved hypothetical protein		-0.27	0.83	0.01
ABAYE1109	conserved hypothetical protein%3B putative signal peptide		-0.21	0.86	0.00
ABAYE1129	conserved hypothetical protein%3B putative hydrolase		2.10	4.29	0.00
ABAYE1148	conserved hypothetical protein%3B putative signal pepetide		0.37	1.29	0.04
ABAYE1153	30S ribosomal protein S2	rpsB	-0.10	0.93	0.04
ABAYE1191	putative secretion pathway ATPase		-0.68	0.62	0.04
ABAYE1192	conserved hypothetical protein%3B putative exported protein		0.23	1.17	0.01
ABAYE1210	Ribonuclease Z (RNase Z) (tRNA 3 endonuclease)	rnz	0.22	1.16	0.04
ABAYE1285	conserved hypothetical protein		-0.36	0.78	0.01
ABAYE1286	30S ribosomal protein S21	rpsU	-0.39	0.76	0.01
ABAYE1331	putative transcriptional regulator (TetR family)		2.10	4.29	0.01
ABAYE1334	paraquat-inducible protein A (part 1)	pqiA-1	-0.10	0.93	0.03

ABAYE1338	conserved hypothetical protein%3B putative transthyretin domain		2.80	6.96	0.00
ABAYE1339	conserved hypothetical protein%3B putative signal peptide		1.80	3.48	0.02
ABAYE1442	16S rRNA pseudouridylate 516 synthase	rsuA	-0.09	0.94	0.00
ABAYE1479	hypothetical protein		2.30	4.92	0.00
ABAYE1485	putative transcriptional regulator (TetR family)		2.00	4.00	0.03
ABAYE1501	conserved hypothetical protein		2.20	4.59	0.01
ABAYE1511	putative transcriptional regulator (LysR family)		0.39	1.31	0.03
ABAYE1544	nitrite reductase%2C large subunit%2C nucleotide-and Fe/S-cluster binding	nasD	0.40	1.32	0.00
ABAYE1604	conserved hypothetical protein		0.19	1.14	0.03
ABAYE1611	conserved hypothetical protein%3B putative signal peptide		2.00	4.00	0.00
ABAYE1633	conserved hypothetical protein		0.18	1.13	0.02
ABAYE1665	conserved hypothetical protein		0.28	1.21	0.02
ABAYE1679	gamma-carboxymuconolactone decarboxylase (CMD)	pcaC	0.72	1.65	0.01
ABAYE1685	quinate/shikimate dehydrogenase [Pyrroloquinoline-quinone] (NAD(P)-independent quinate dehydrogenase)	quiA	0.29	1.22	0.01
ABAYE1700	putative Amidase		1.20	2.30	0.04
ABAYE1702	conserved hypothetical protein		1.60	3.03	0.00
ABAYE1703	conserved hypothetical protein		1.50	2.83	0.04

ABAYE1707	putative Ferredoxin (Oxidoreductase FAD/NAD(P)-binding region)%3B putative aromatic oxidoreductase		0.38	1.30	0.05
ABAYE1711	conserved hypothetical protein%3B putative signal peptide		0.81	1.75	0.02
ABAYE1714	3-oxoadipate enol-lactonase II (Beta-ketoadipate enol-lactone hydrolase II)	catD	0.44	1.36	0.01
ABAYE1715	beta-ketoadipyl CoA thiolase	catF	0.13	1.09	0.04
ABAYE1724	putative dihydroxyacid dehydratase (ilvD-like)		0.47	1.39	0.01
ABAYE1726	conserved hypothetical protein		0.65	1.57	0.00
ABAYE1729	conserved hypothetical protein		2.40	5.28	0.00
ABAYE1734	putative transcriptional regulator (MerR family)		0.58	1.49	0.02
ABAYE1756	putative transcriptional regulator (TetR family)		0.71	1.64	0.00
ABAYE1780	putative NADP-dependent aldehyde dehydrogenase (AldH-like)		0.31	1.24	0.04
ABAYE1816	conserved hypothetical protein%3B putative hydrolase of the alpha/beta superfamily		0.39	1.31	0.03
ABAYE1822	RND protein	adeB	-1.20	0.44	0.00
ABAYE1833	hypothetical protein		2.80	6.96	0.00
ABAYE1856	putative fimbrial protein precursor (Pilin)		0.75	1.68	0.03
ABAYE1863	conserved hypothetical protein%3B putative catalase		-1.30	0.41	0.00
ABAYE1864	hypothetical protein		-6.00	0.02	0.00
ABAYE1866	hypothetical protein		-8.70	0.00	0.00

ABAYE1870	fragment of putative phospholipase D protein (part 1)		-3.30	0.10	0.01
ABAYE1871	fragment of putative phospholipase D protein (part 2)		-7.50	0.01	0.00
ABAYE1874	putative G:T/U mismatch-specific DNA glycosylase		-8.40	0.00	0.00
ABAYE1875	hypothetical protein		-6.40	0.01	0.00
ABAYE1876	hypothetical protein		-5.80	0.02	0.00
ABAYE1877	Zn-dependent dipeptidase	acdP	-8.30	0.00	0.00
ABAYE1878	coenzyme PQQ synthesis protein E (Coenzyme PQQ synthesis protein III)	pqqE	-9.10	0.00	0.00
ABAYE1879	coenzyme PQQ synthesis protein D (Coenzyme PQQ synthesis protein II)	pqqD	-7.50	0.01	0.00
ABAYE1880	coenzyme PQQ synthesis protein C (Coenzyme PQQ synthesis protein I)	pqqC	-9.60	0.00	0.00
ABAYE1881	coenzyme PQQ synthesis protein B (Coenzyme PQQ synthesis protein V)	pqqB	-9.80	0.00	0.00
ABAYE1882	coenzyme PQQ synthesis protein A (Coenzyme PQQ synthesis protein IV)	pqqA	-6.20	0.01	0.00
ABAYE1884	fragment of polyphosphate kinase (Polyphosphoric acid kinase) (ATP-polyphosphate phosphotransferase) (part 2)	ppk	-6.50	0.01	0.00
ABAYE1885	fragment of polyphosphate kinase (Polyphosphoric acid kinase) (ATP-polyphosphate phosphotransferase) (part 1)	ppk	-10.00	0.00	0.00
ABAYE1886	conserved hypothetical protein%3B putative 5`-nucleotidase domain		-6.30	0.01	0.00
ABAYE1887	putative Ferric uptake regulator		-2.90	0.13	0.02
ABAYE1888	putative Isochorismatase (Isochorismate lyase) (2%2C3 dihydro-2%2C3 dihydroxybenzoate synthase)		-7.10	0.01	0.00

ABAYE1889	putative 2%2C3-Dihydro-2%2C3-dihydroxybenzoate dehydrogenase		-7.10	0.01	0.00
ABAYE1890	hypothetical protein		-2.80	0.14	0.01
ABAYE1892	molybdate transport protein (ABC superfamily%2C atp_bind)	modC	-6.50	0.01	0.00
ABAYE1893	molybdate transport protein (ABC superfamily%2C membrane)	modB	-7.50	0.01	0.00
ABAYE1894	molybdate transport protein (ABC superfamily%2C peri_bind)	modA	-8.00	0.00	0.00
ABAYE1895	putative repressor of the modABC operon for molybdenum transport (ModE)		-2.40	0.19	0.00
ABAYE1896	anthranilate dioxygenase large subunit	antA	-4.90	0.03	0.00
ABAYE1897	anthranilate dioxygenase small subunit	antB	-4.40	0.05	0.00
ABAYE1898	anthranilate dioxygenase reductase	antC	-6.00	0.02	0.00
ABAYE1900	putative transcriptional regulator		-7.30	0.01	0.00
ABAYE1901	conserved hypothetical protein%3B putative exported protein		-3.70	0.08	0.00
ABAYE1902	conserved hypothetical protein		-3.00	0.13	0.00
ABAYE1903	conserved hypothetical protein		-4.00	0.06	0.01
ABAYE1904	putative oxidoreductase%2C short-chain dehydrogenase/reductase family		-4.60	0.04	0.00
ABAYE1905	fragment of putative carboxymethylenebutenolidase (Dienelactone hydrolase) (DLH) (part 2)		-2.70	0.15	0.01
ABAYE1906	fragment of putative carboxymethylenebutenolidase (Dienelactone hydrolase) (DLH) (part 1)		-4.40	0.05	0.00
ABAYE1907	putative Permease of the major facilitator superfamily		-5.00	0.03	0.00

ABAYE1908	putative transcriptional regulator		-7.20	0.01	0.00
ABAYE1909	3-hydroxybutyrate dehydrogenase	bdhA	-7.50	0.01	0.00
ABAYE1910	putative D-beta-hydroxybutyrate permease		-7.80	0.00	0.00
ABAYE1911	hypothetical protein		-3.60	0.08	0.01
ABAYE1912	putative transcriptional regulator (LysR family)		-3.20	0.11	0.00
ABAYE1926	putative transport protein (ABC superfamily%2C atp_bind)%3B putative nitrate ABC transporter%2C ATP-binding protein		0.92	1.89	0.05
ABAYE1928	putative transport protein (ABC superfamily%2C peri_bind)%3B putative nitrate ABC transporter%2C periplasmic		0.45	1.37	0.02
ABAYE1951	conserved hypothetical protein		-0.40	0.76	0.05
ABAYE1971	putative poly(R)-hydroxyalkanoic acid synthase		0.27	1.21	0.03
ABAYE1972	putative methyltransferase		0.33	1.26	0.01
ABAYE1981	conserved hypothetical protein		0.34	1.27	0.03
ABAYE1985	conserved hypothetical protein%3B putative membrane protein		1.10	2.14	0.02
ABAYE1987	conserved hypothetical protein%3B putative membrane protein		1.70	3.25	0.04
ABAYE2012	putative acyl-CoA dehydrogenase		0.17	1.13	0.01
ABAYE2014	terminal alkane-1-monooxygenase	alkM	0.48	1.39	0.00
ABAYE2018	putative poly(hydroxyalcanoate) granule associated protein		0.24	1.18	0.04
ABAYE2024	co-chaperone protein (Hsc20)%2C believed to be involved in assembly of Fe-S	hscB	0.23	1.17	0.02

	clusters				
ABAYE2032	conserved hypothetical protein		0.25	1.19	0.01
ABAYE2043	putative metallopeptidase		0.29	1.22	0.02
ABAYE2050	putative biopolymer transport protein (EXBD-like)		0.40	1.32	0.04
ABAYE2051	putative biopolymer transport protein (EXBD-like)		0.66	1.58	0.01
ABAYE2074	type 4 fimbrial biogenesis protein	pilZ	-0.20	0.87	0.02
ABAYE2091	conserved hypothetical protein		0.23	1.17	0.02
ABAYE2103	glycine cleavage complex protein H%2C carrier of aminomethyl moiety via covalently bound lipoyl cofactor	gcvH	-0.27	0.83	0.02
ABAYE2120	conserved hypothetical protein		1.30	2.46	0.04
ABAYE2140	hypothetical protein%3B putative exported protein		2.80	6.96	0.00
ABAYE2152	putative transcriptional regulator (TetR family)		-0.27	0.83	0.01
ABAYE2157	conserved hypothetical protein%3B putative exported protein		0.32	1.25	0.00
ABAYE2177	putative membrane protein		0.40	1.32	0.05
ABAYE2178	conserved hypothetical protein		0.29	1.22	0.02
ABAYE2195	putative chromate transport protein		0.68	1.60	0.03
ABAYE2198	putative arsenite efflux transporter		0.58	1.49	0.01
ABAYE2199	putative transcriptional regulator (ArsR family)		2.70	6.50	0.00

ABAYE2200	Arsenate reductase		0.63	1.55	0.05
ABAYE2205	putative transcriptional regulator		0.29	1.22	0.02
ABAYE2207	putative permease		0.20	1.15	0.03
ABAYE2223	putative bifunctional protein [Includes: putative transcriptional regulator (GntR family)%3B putative amino transferase]		0.29	1.22	0.00
ABAYE2229	malonate decarboxylase%2C gamma subunit	mdcE	0.37	1.29	0.03
ABAYE2236	conserved hypothetical protein%3B putative acetyltransferase		0.28	1.21	0.01
ABAYE2277	hypothetical protein%3B putative exported protein		1.70	3.25	0.01
ABAYE2291	conserved hypothetical protein%3B putative Biotin carboxylase		0.81	1.75	0.05
ABAYE2292	putative Hydroxymethylglutaryl-CoA lyase (HMG-CoA lyase) (HL) (3-hydroxy-3-methylglutarate-CoA lyase)(MvaB-like)		0.88	1.84	0.03
ABAYE2302	transport protein in catabolism of dicarboxylic acids (MFS superfamily)	dcaK	0.62	1.54	0.05
ABAYE2306	dehydrogenase	dcaH	0.44	1.36	0.03
ABAYE2328	putative gamma-carboxymuconolactone decarboxylase		0.42	1.34	0.03
ABAYE2438	Acetyl-/propionyl-coenzyme A carboxylase alpha chain [Includes: Biotin carboxylase %3B Biotin carboxyl carrier protein (BCCP)]	bccA	1.10	2.14	0.01
ABAYE2439	putative allophanate hydrolase subunit 1 and 2		1.10	2.14	0.01
ABAYE2440	conserved hypothetical protein		1.10	2.14	0.01
ABAYE2441	putative lactam utilization protein		1.10	2.14	0.01

ABAYE2442	conserved hypothetical protein%3B putative membrane protein		1.10	2.14	0.00
ABAYE2466	putative transcriptional regulator (TetR family)		0.62	1.54	0.02
ABAYE2531	hypothetical protein		2.00	4.00	0.01
ABAYE2543	putative exodeoxyribonuclease VII small subunit (Exonuclease VII small subunit)		0.32	1.25	0.00
ABAYE2546	conserved hypothetical protein		0.45	1.37	0.02
ABAYE2553	transcriptional regulator of benzoate degradation (LysR family)	benM	-0.29	0.82	0.04
ABAYE2562	putative 3-oxoacyl-[acyl-carrier-protein] synthase III		-0.16	0.90	0.02
ABAYE2579	conserved hypothetical protein		0.72	1.65	0.01
ABAYE2591	conserved hypothetical protein%3B putative membrane protein		-0.66	0.63	0.01
ABAYE2613	putative short-chain dehydrogenase		0.56	1.47	0.04
ABAYE2634	conserved hypothetical protein		1.10	2.14	0.00
ABAYE2663	putative bifunctional protein [Includes: lytic murein transglycosylase C%2C membrane-bound (MltD)%3B cell wall hydrolase]		-0.04	0.97	0.02
ABAYE2667	esterase	aesT	-0.24	0.85	0.04
ABAYE2669	conserved hypothetical protein		-0.48	0.72	0.01
ABAYE2674	putative Cation efflux system protein		-0.29	0.82	0.04
ABAYE2689	conserved hypothetical protein		1.90	3.73	0.00
ABAYE2700	hypothetical protein%3B putative exported protein		2.20	4.59	0.00

ABAYE2764	glutamate dehydrogenase%2C NADP-specific	gdhA	-0.21	0.86	0.03
ABAYE2821	conserved hypothetical protein		2.10	4.29	0.00
ABAYE2823	putative transketolase C-terminal section		0.33	1.26	0.04
ABAYE2832	putative Glutaminase		-0.21	0.86	0.05
ABAYE2836	putative acetolactate synthase (IlvB-like)		0.54	1.45	0.02
ABAYE2839	putative short-chain dehydrogenase		0.43	1.35	0.00
ABAYE2847	conserved hypothetical protein		0.45	1.37	0.01
ABAYE2849	putative transcriptional regulator (PcaU-like)		0.24	1.18	0.02
ABAYE2915	ATP-dependent dsDNA exonuclease (Suppression of recBC)	sbcD	-0.20	0.87	0.04
ABAYE2918	twitching motility protein	pilT	-0.87	0.55	0.03
ABAYE2919	twitching motility protein	pilU	-0.90	0.54	0.01
ABAYE2941	leucyl%2C phenylalanyl-tRNA-protein transferase	aat	0.11	1.08	0.03
ABAYE2952	conserved hypothetical protein%3B putative membrane protein		-0.28	0.82	0.01
ABAYE3036	putative multidrug resistance efflux pump		0.16	1.12	0.01
ABAYE3045	hypothetical protein		0.70	1.62	0.02
ABAYE3052	NADH dehydrogenase I chain J	nuoJ	0.20	1.15	0.02
ABAYE3053	NADH dehydrogenase I chain I%2C 2Fe-2S ferredoxin-related	nuoI	0.27	1.21	0.00
ABAYE3054	NADH dehydrogenase I chain H	nuoH	0.22	1.16	0.00

ABAYE3055	NADH dehydrogenase I chain G	nuoG	0.14	1.10	0.02
ABAYE3056	NADH dehydrogenase I chain F	nuoF	0.23	1.17	0.02
ABAYE3057	NADH dehydrogenase I chain E	nuoE	0.23	1.17	0.02
ABAYE3085	putative Transcriptional Regulator (AraC family)		2.50	5.66	0.00
ABAYE3090	putative substrate-binding protein (ABC superfamily%2C peri-bind)		0.43	1.35	0.04
ABAYE3091	putative transcriptional regulator (LysR family)		0.24	1.18	0.02
ABAYE3123	putative pilus assembly protein (FilF)		1.90	3.73	0.00
ABAYE3143	putative transcriptional regulator (MarR family)		0.27	1.21	0.04
ABAYE3162	hypothetical protein%3B putative exported protein		1.20	2.30	0.01
ABAYE3167	putative glutathione S-transferase		-0.21	0.86	0.03
ABAYE3191	pyridine nucleotide transhydrogenase%2C beta subunit	pntB	1.10	2.14	0.00
ABAYE3192	pyridine nucleotide transhydrogenase (proton pump)%2C alpha subunit (part2)	pntA-2	1.20	2.30	0.00
ABAYE3193	pyridine nucleotide transhydrogenase (proton pump)%2C alpha subunit (part1)	pntA-1	1.00	2.00	0.00
ABAYE3218	hypothetical protein		0.81	1.75	0.02
ABAYE3282	acetate kinase (propionate kinase)	ack	-0.31	0.81	0.04
ABAYE3286	conserved hypothetical protein%3B putative exported protein		-0.37	0.77	0.03
ABAYE3304	fragment of putative phospholipase D protein (part 1)		-4.20	0.05	0.00

ABAYE3305	fragment of putative phospholipase D protein (part 2)		-7.80	0.00	0.00
ABAYE3333	conserved hypothetical protein%3B putative membrane protein		-0.13	0.91	0.04
ABAYE3334	conserved hypothetical protein%3B putative signal peptide		-0.31	0.81	0.00
ABAYE3347	conserved hypothetical protein%3B putative membrane protein		-0.20	0.87	0.04
ABAYE3394	conserved hypothetical protein		-0.33	0.80	0.00
ABAYE3403	conserved hypothetical protein%3B putative membrane protein		-0.25	0.84	0.01
ABAYE3445	type 4 fimbrial assembly protein	pilC	-0.85	0.55	0.01
ABAYE3446	type 4 prepilin-like proteins leader peptide processing enzyme (Protein secretion protein XCPA)[Includes: Leader peptidase (Prepilin peptidase)%3B N-methyltransferase]	pilD	-0.53	0.69	0.01
ABAYE3447	dephosphocoenzyme A kinase	coaE	-0.33	0.80	0.04
ABAYE3517	conserved hypothetical protein%3B putative exported protein		0.24	1.18	0.03
ABAYE3535	type 4 fimbriae expression regulatory protein	pilR	-0.67	0.63	0.03
ABAYE3553	putative sulfate transporter (MFS superfamily)		2.00	4.00	0.01
ABAYE3658	Protein arsC (Arsenate reductase) (Arsenical pump modifier) (Low molecular weight protein-tyrosine-phosphatase)	arsC	2.80	6.96	0.00
ABAYE3659	arsenite efflux transporter	arsB	0.53	1.44	0.02
ABAYE3662	putative monooxygenase		2.50	5.66	0.01
ABAYE3694	conserved hypothetical protein%3B putative flavodoxin		0.34	1.27	0.00

ABAYE3697	putative acetyltransferase		-0.29	0.82	0.02
ABAYE3700	putative transcription regulator protein (TetR family)		0.65	1.57	0.03
ABAYE3704	conserved hypothetical protein%3B putative exported protein		0.22	1.16	0.01
ABAYE3706	conserved hypothetical protein%3B putative exported protein		-0.80	0.57	0.03
ABAYE3731	NAD-linked malate dehydrogenase%2C Rossman fold	sfcA	-0.25	0.84	0.00
ABAYE3751	putative bifunctional protein [Includes: NAD-dependent epimerase/dehydratase%3B Carboxylesterase]		0.31	1.24	0.04
ABAYE3752	conserved hypothetical protein%3B putative porin		0.62	1.54	0.03
ABAYE3754	putative Non-ribosomal peptide synthetase		0.85	1.80	0.03
ABAYE3756	putative acyl-CoA dehydrogenase		0.88	1.84	0.05
ABAYE3758	putative Autoinducer-binding transcriptional regulator (LuxR family)		-0.28	0.82	0.04
ABAYE3760	conserved hypothetical protein		0.59	1.51	0.04
ABAYE3764	putative Enoyl-CoA hydratase		0.39	1.31	0.01
ABAYE3765	putative acyl-CoA dehydrogenase		0.44	1.36	0.01
ABAYE3766	putative acetyl-coA synthetase/AMP-(fatty) acid ligase		0.88	1.84	0.03
ABAYE3767	3-hydroxyisobutyrate dehydrogenase	mmsB	1.00	2.00	0.05
ABAYE3781	conserved hypothetical protein		0.19	1.14	0.03
ABAYE3794	putative transcriptional regulator (GntR family)		-0.52	0.70	0.03

ABAYE3815	putative UDP-glucose/GDP-mannose dehydrogenase		2.30	4.92	0.00
ABAYE3838	conserved hypothetical protein%3B putative exported protein		0.73	1.66	0.01
ABAYE3893	fragment of putative phospholipase D protein (part 1)		-4.10	0.06	0.00
ABAYE3894	fragment of putative phospholipase D protein (part 2)		-7.70	0.00	0.00
ABAYEtRNA13	ABAYEtRNA13		2.20	4.59	0.00
ABAYEtRNA21	ABAYEtRNA21		2.10	4.29	0.00
ABAYEtRNA26	ABAYEtRNA26		2.00	4.00	0.00
ABAYEtRNA57	ABAYEtRNA57		1.20	2.30	0.01
ABAYEtRNA59	ABAYEtRNA59		1.20	2.30	0.04
ABAYEtRNA60	ABAYEtRNA60		1.30	2.46	0.00
ABAYEtRNA62	ABAYEtRNA62		1.90	3.73	0.00
ABAYEtRNA63	ABAYEtRNA63		2.40	5.28	0.00
ABAYEtRNA64	ABAYEtRNA64		1.80	3.48	0.02
ABAYEtRNA65	ABAYEtRNA65		1.80	3.48	0.01
ABAYEtRNA71	ABAYEtRNA71		1.20	2.30	0.01
ABAYEtRNA72	ABAYEtRNA72		2.40	5.28	0.00
ABAYEtRNA8	ABAYEtRNA8		1.80	3.48	0.02
